# Peptidines: glycine-amidine-based oligomers for solution- and solid-phase synthesis†
†Electronic supplementary information (ESI) available: Experimental procedures, characterization data of new compounds. CCDC 1430515. For ESI and crystallographic data in CIF or other electronic format see DOI: 10.1039/c5sc03882k


**DOI:** 10.1039/c5sc03882k

**Published:** 2016-02-16

**Authors:** Julian Vastl, Rendy Kartika, Kichul Park, Art E. Cho, David A. Spiegel

**Affiliations:** a Department of Chemistry , Yale University , 225 Prospect Street , New Haven , CT 06511 , USA . Email: David.Spiegel@yale.edu; b Department of Chemistry , Louisinanna State University , 337 Chemistry and Materials Building , Baton Rouge , LA 70803 , USA; c Department of Bioinformatics , Korea University Sejong Campus , 2511 Sejong-ro , Sejong City 399-770 , Korea; d Department of Pharmacology , Yale University , 333 Cedar Street , New Haven , CT 06520 , USA

## Abstract

Efforts to emulate biological oligomers have given rise to a host of useful technologies, ranging from solid-phase peptide and nucleic acid synthesis to various peptidomimetic platforms. Herein we introduce a novel class of peptide-like oligomers called “peptidines” wherein each carbonyl O-atom within poly-*N*-alkyl glycine oligomers is replaced with a functionalized N-atom. Compared to peptoids or peptides, the presence of this amidine *N*-substituent in peptidines effectively doubles the number of diversification sites per monomeric unit, and can decrease their overall conformational flexibility. We have developed iterative solution- and solid-phase protocols for the straightforward assembly of peptidines containing diverse backbone and amidine substituents, derived from readily available primary and secondary amines. We have also performed crystallographic and computational studies, which demonstrate a strong preference for the *trans* (*E*) amidine geometry. Given their straightforward synthetic preparation and high functional group density, peptidines have the potential to serve as useful tools for library generation, peptide mimicry, and the identification of biologically active small molecules.

## Abstract 2

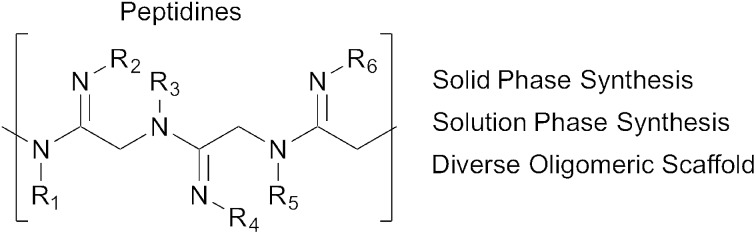
We introduce a modular synthetic procedure to produce a new class of synthetic oligomers called peptidines composed of repeating di-substituted glycine-derived amidines.

## Introduction

Oligomer-based synthesis is central to all known life processes. In particular, the structural and functional variety found in proteins is derived from the assembly of only 20 amino acid building blocks. Efforts to emulate this diversity have led to a range of oligomer-based peptidomimetic strategies, including oligopeptides, peptoids, peptidosulfonamides, sulfonylpeptides, polypyrroles and others.^1^–^4^ In turn, these strategies have given rise to numerous exciting applications, including combinatorial library synthesis,^2^,^5^ solid-supported screening,^6^–^8^ and biomarker discovery,^9^–^11^ making the development of novel oligomer-based approaches to achieve structurally-defined compound libraries a highly desirable endeavour.^12^,^13^

**Fig. 1:**
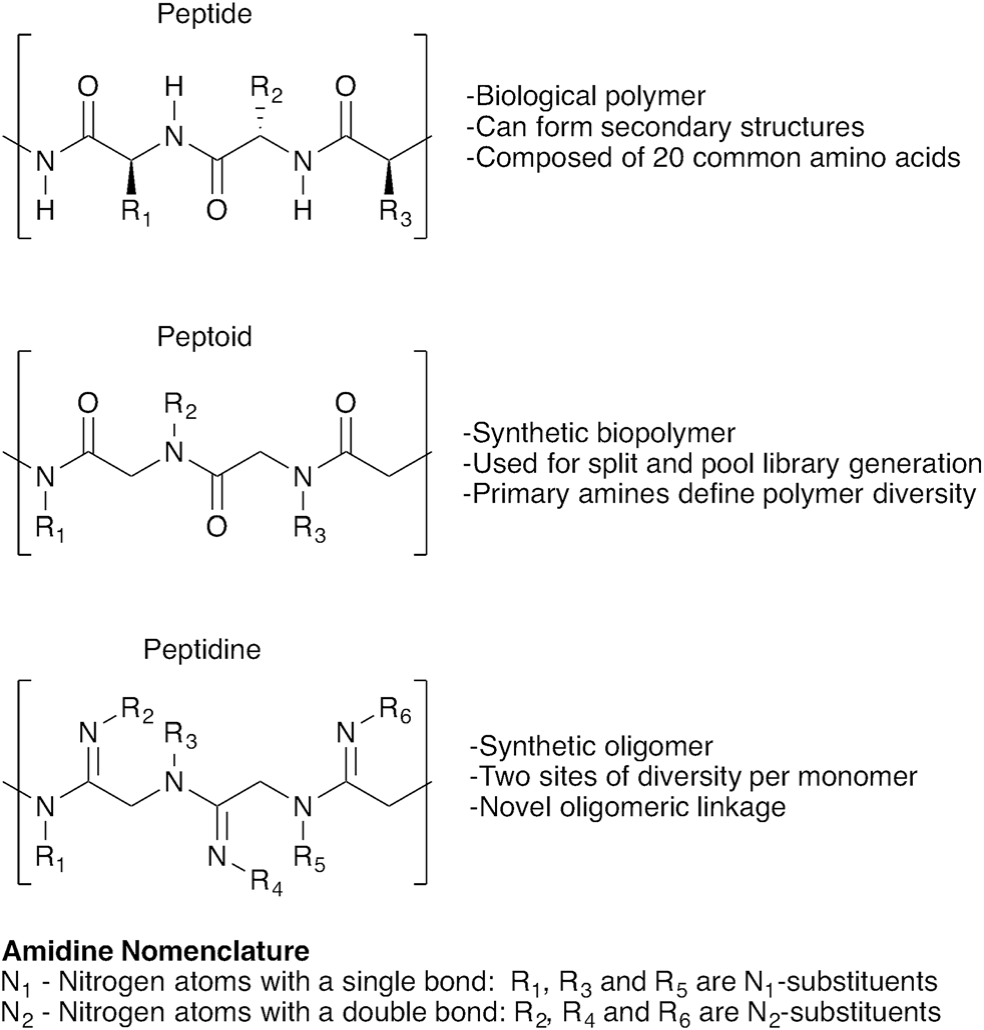
A structural comparison of peptides, peptoids and peptidines.

Herein we introduce a new class of oligomeric scaffolds that we term “peptidines”. Peptidines are oligomers composed of repeating di-substituted glycine-derived amidines (Fig. 1). Although similar to peptides and peptoids, the peptidine scaffold accommodates two substituents per monomeric unit by replacing C=O, with C=NR thus doubling the accessible diversity for a given oligomer length. By varying the size and electronics of amidine *N*^1^ (backbone) and *N*^2^ (amidino) substituents, one can also modulate *N*-lone pair basicity, and backbone geometry.

Thus, we have developed a concise peptidine synthesis protocol that allows both *N*^1^ and *N*^2^ substituents to derive modularly from the large pool of commercially available primary amines. Using this route, we have been able to produce peptidines ranging in size from 2- to 4-mers, appended with sterically and electronically diverse substituents at both *N*^1^ and *N*^2^ positions. These syntheses proceed in short order, and with excellent yields in both solution and solid phases. Crystallographic and computational studies have demonstrated that amidines present within the peptidine scaffold prefer the *trans*-(*E*) geometry of the *N*^1^ substituent with respect to the *N*^2^ nitrogen. Peptidines therefore adopt discrete conformations as a function of both H-bonding effects and non-bonding interactions. In light of their facile preparation and high potential for chemical conformational diversification, we envision that peptidines will provide a useful scaffold for the preparation of novel and diverse structures with a range of chemical and biological applications.

## Results and discussion

**Scheme 1:**
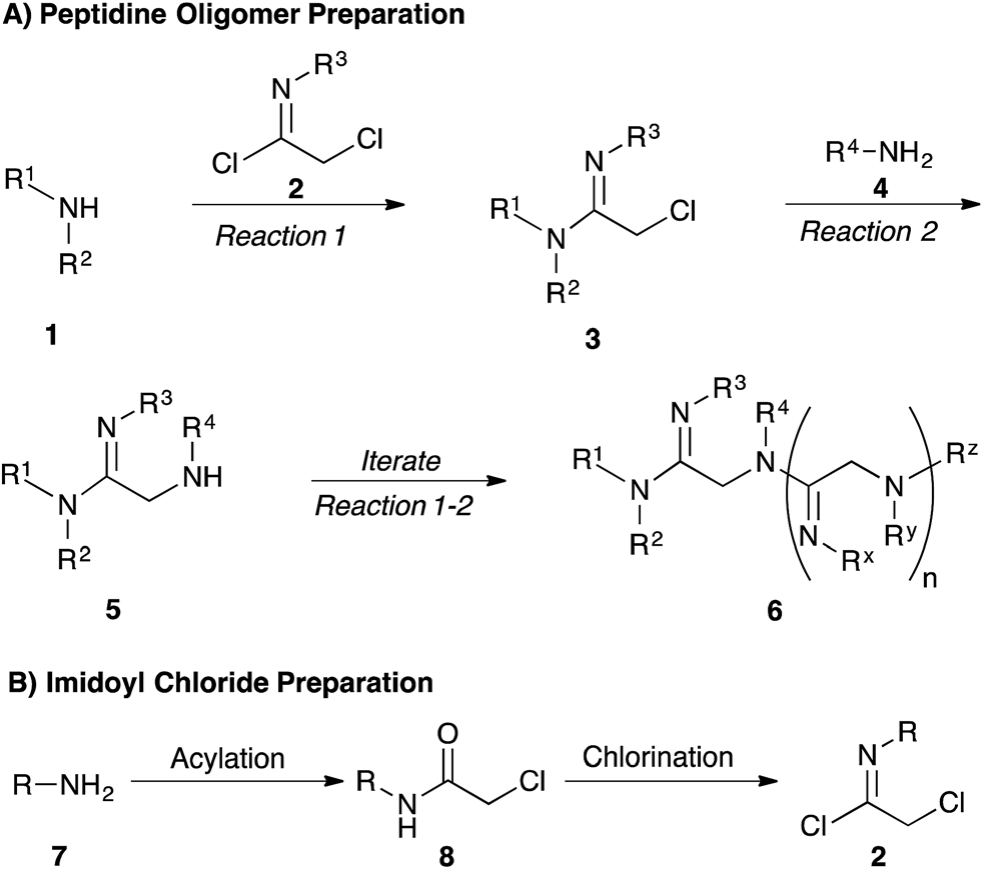
Modular synthesis of peptidines.

Our strategy for preparing peptidines is outlined in Scheme 1. As shown, this protocol is composed of two separate, iteratively-applied synthetic transformations: first is an amidination step (Scheme 1A, reaction 1), wherein we amidinate a secondary amine (**1**) with an imidoyl chloride (**2**) to produce an α-chloro amidine (**3**); second is an amination step (reaction 2), wherein the chloride atom in **3** is displaced by a primary amine (**4**) to form an α-amino amidine (**5**). Iteration of reactions 1 and 2 affords peptidine oligomers (**6**). In turn, the α-chloro imidoyl chlorides (**2**) used in reaction 1 are generated from commercially available primary amines (**7**), which are acylated to produce chloroamides (**8**), and then chlorinated to form imidoyl chlorides (**2**) (Scheme 1B).

We first focused on converting α-chloro-amides (**8a–i**) to the corresponding imidoyl chlorides (**2a–i**, Table 1, reaction 1). This transformation was accomplished with substantial generality using PCl_5_ in refluxing benzene.^14^,^15^ Although the poor hydrolytic stability of intermediate imidoyl chlorides precluded traditional work-up and purification protocols, ^1^H-NMR analysis of crude reaction mixtures indicated ≥95% purity, alleviating the need for further purification. These intermediates were stable at room temperature for months without observable decomposition upon storage under moisture-free conditions as 1 M stock solutions in DCM.

**Table 1:** Modular synthesis of α-amino-amidines^*a*^

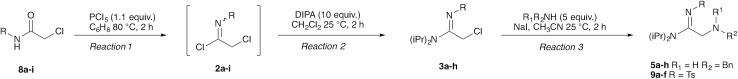								
Entry	Amide	R	Product	Yield **3a–h**^*b*^ (2-step)	R^1^	R^2^	Product	Yield **5a–h**/**9a–f**^*c*^
1	**8a**	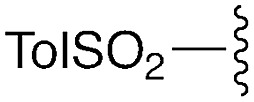	**3a**	93%	H	Bn	**5a**	96%
2	**8b**	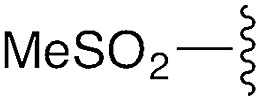	**3b**	86%	H	Bn	**5b**	95%
3	**8c**	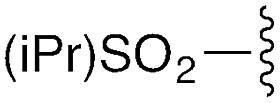	**3c**	88%	H	Bn	**5c**	92%
4	**8d**	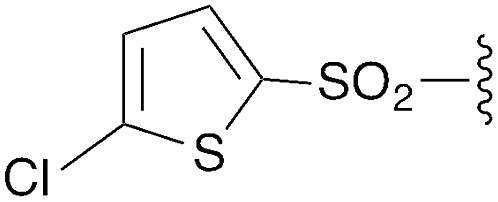	**3d**	88%	H	Bn	**5d**	99%
5	**8e**	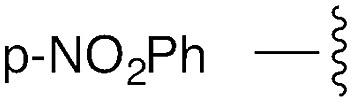	**3e**	72%	H	Bn	**5e**	90%
6^*d*^	**8f**	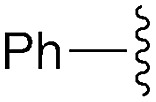	**3f**	70%	H	Bn	**5f** ^*e*^	91%
7	**8g**	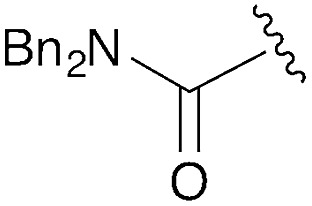	**3g**	83%	H	Bn	**5g**	96%
8	**8h**	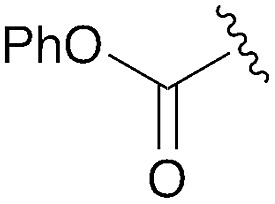	**3h**	79%	H	Bn	**5h** ^*f*^	92%
9	**8i**	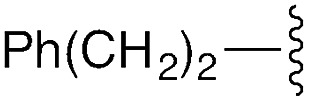	**3i**	—^*g*^	—	—	—	—
10	**8a**	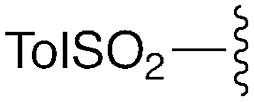	—	—	H	Cy	**9a**	96%
11^*h*^	**8a**	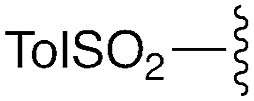	—	—	H	Ph	**9b**	90%
12	**8a**	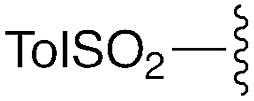	—	—	Et	Et	**9c**	99%
13	**8a**	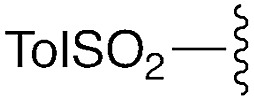	—	—	–(CH_2_)_4_–		**9d**	92%
14	**8a**	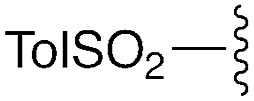	—	—	H	CHPh_2_	**9e**	72%
15^*i*^	**8a**	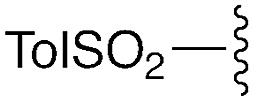	—	—	H	^*t*^Bu	**9f**	0%

^*a*^DIPA = diisopropyl amine. Cy = cyclohexyl.

^*b*^Isolated yield from **8a–h**.

^*c*^Isolated yield from **3a–h**.

^*d*^Reaction 1 was run at 25 °C; reaction 2 was run for 14 hours.

^*e*^Product was isolated as a hydrochloride salt. See ESI for details.

^*f*^Reaction 3 product rapidly cyclizes to form 1-benzyl-4-(diisopropylamino)-1,5-dihydro-2*H*-imidazol-2-one (ESI Fig. 2).

^*g*^We were unable to isolate **3i** due to difficulties in purification, as described in the text. The corresponding *N*^1^-diethylamidine (**37**) was prepared in 78% yield, as detailed in ESI Fig. 1†.

^*h*^Reaction 3 was carried out in the absence of NaI at 80 °C for 12 hours.

^*i*^No product was observed by LCMS.

We next focused on amine amidination with imidoyl chlorides (Table 1, reaction 2). Thus, treatment of intermediates **2a–i** with diisopropylamine (intended to serve as a solution-phase model for sterically-hindered, resin-bound amine) successfully afforded sulfonyl- (**3a–d**), aryl (**3e–f**), carbamoyl-, and urea-derived (**3g–h**) amidines. These yields were universally high (>70%) throughout a range of *N*^2^-substituents and as expected, reactions with sulfonamides proceeded faster (14 h *versus* 2 h), and in higher yields, than aryl and acyl derivatives. Furthermore, despite the possibility for reaction at the alkyl chloride position, we observed complete chemoselectivity for displacement at the acylimino carbon for all substrates examined, with no evidence of double addition. Similar chemoselectivities have been observed previously in condensation reactions between bis-electrophiles such as α-chloro-acid chlorides and secondary amines.^16^ Although alkyl imidoyl chloride **2i** did appear to provide the corresponding α-chloro-amidine (**3i**) following diisopropylamine treatment, attempts to purify this compound were hampered by the presence of inseparable amounts of diisopropylammonium chloride and diisopropylamine. However, by carrying out the chlorination reaction at room temperature to avoid decomposition (likely *via* polymerization), followed by trapping with diethylamine, we were able to access alkyl amidine **37** (ESI Fig. 1†). Despite this one example, we found *N*^2^-alkyl-substituted intermediates to be highly unstable, leading to difficulties in isolation and purification. We therefore elected not to pursue this electron-rich substrate class further.

Having demonstrated the ability to produce a wide array of α-chloro amidines (**3a–h**), we next focused our efforts on displacing the pendant chloride with amine nucleophiles (Table 1, reaction 3). We conducted these reactions using benzylamine as a model primary amine nucleophile and used an excess of this reagent to mimic the conditions that we would be using to produce these oligomers on solid phase. We found that treatment of α-chloro amidines with both primary and secondary amines in the presence of iodide afforded the corresponding α-amino amidines (**5a–h**) in excellent yields.^17^ Reactions employing benzylamine as the nucleophilic component proceeded smoothly with electron poor sulfonamides (Table 1, entries 1–4) and *p*-nitrophenyl (entry 5) derivatives to give α-amino amidines (**5a–e**) in near quantitative yields. Interestingly, the phenyl derivative **5f** was found to decompose rapidly as the free base, however addition of hydrochloric acid in ether to this compound immediately after silica gel purification facilitated its isolation as the stable HCl salt. Furthermore, the reaction of **3h** with benzylamine produced cyclic amidine **5h** (ESI Fig. 2†) in nearly quantitative yield, with no acyclic product observed by LC/MS.

We next analyzed how varying the structure of the amine nucleophile would affect the chloride displacement process using substrate **3a** as the α-chloro amidine component (Table 1 entries 10–15). Overall, this reaction proceeded in greater than 90% yield using cyclohexylamine and aniline as nucleophiles to yield **9a** and **9b** respectively, although the latter substrate required heating. Similarly high yields were observed for secondary amine nucleophiles, such as diethylamine and pyrrolidine, affording **9c** and **9d** respectively. Decreases in yield were observed for the sterically bulky amines shown in entries 14 and 15. Benzhydrylamine provided **9e** in 72% yield and *t*-butylamine was incapable of reacting with **3a**, and afforded none of the desired product (**9f**). Taken together, these results confirm that the peptidine core can be constructed through a three-step procedure involving: (1) amide chlorination, (2) amidination of a secondary amine, and (3) α-halide displacement with a primary or secondary amine.

**Scheme 2:**
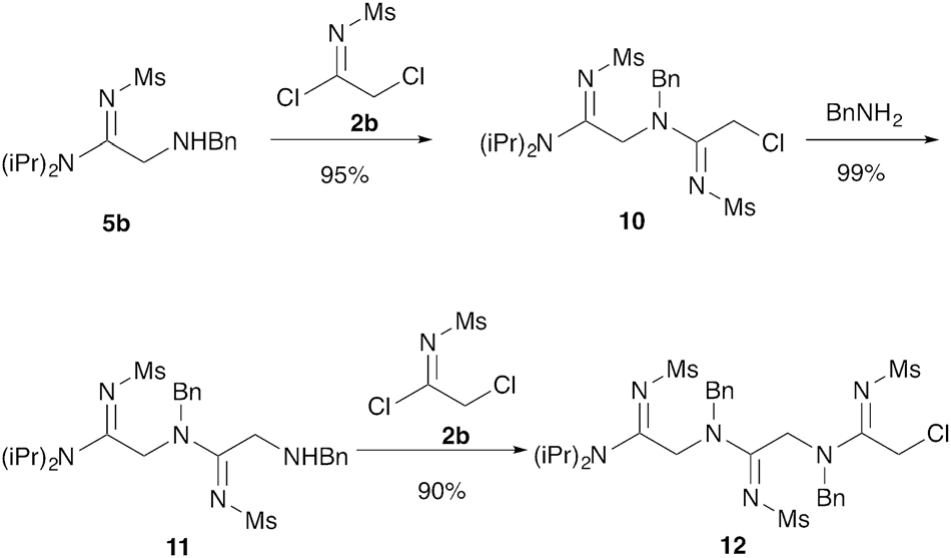
Elongation to form trimer peptidines. Treatment with **2b** (1.5 equiv.) was carried out in CH_3_CN at 25 °C with NMM (3 equiv.) for 3 hours. Animation was carried out with benzylamine (5 equiv.) sodium iodide (5 equiv.) in CH_3_CN at 25 °C for 2 hours.

Our next goal was to iterate the above three-step sequence to access longer oligomeric peptidines. We therefore chose to target a simple 3-mer composed of identical repeating monomeric units (Scheme 2). Starting from α-chloro amidine **5b**, and using imidoyl chloride **2b** and benzylamine as nucleophile, we were able to access 2-mers **10** and **11**, and 3-mer **12** in only three steps and 80% overall yield by simply repeating chloride displacement and amidination steps. Notably, none of these transformations proceeded in lower than 90% yield. Furthermore, using this protocol, we have been able to prepare **12** in quantities greater than 1 g demonstrating the scalability of this procedure.

Efforts to advance 3-mer **12** into longer oligomers gave rise to several notable findings. For example, treatment of **12** with benzylamine led exclusively to cyclic product **13** (Table 2, entry 1), as characterized by NMR and LCMS. Because formation of **12** proceeds without evidence of cyclic byproducts, we hypothesize that the diisopropyl groups in **11** protect the C-terminal amidine from internal nucleophilic attack by the appended secondary amine. Although **13** is incapable of further elongation, this observation demonstrates that the peptidine platform is capable of giving rise to nonlinear molecular architectures. Replacement of benzylamine with (*S*)-methylbenzylamine afforded no cyclic product upon reaction with **12**, but instead exclusively provided the expected linear product **14** (Table 2, entry 2) in 99% yield. We hypothesize that the additional methyl group in (*S*)-methylbenzylamine provides sufficient steric encumbrance to prevent internal cyclization. Switching the nucleophile to Bn_2_NH provided linear 3-mer **15**. We also succeeded in producing a linear 4-mer peptidine (**16**) *via* our two-step elongation protocol (Table 2, entry 4); however unlike other acylation reactions, formation of **16** required heating to 60 °C to reach completion, most likely due to steric hindrance surrounding the amine terminus in the starting material (**14**). These results demonstrate that by regulating the steric environment around backbone *N*^1^ substituents, we can access both linear and cyclic peptidines.

**Table 2:** Tetramer synthesis and cyclization

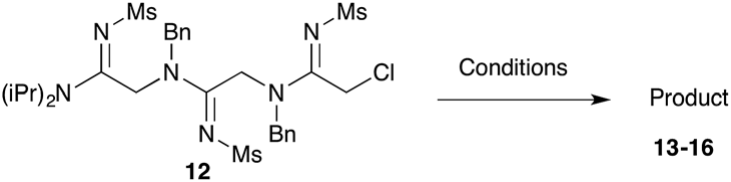				
Entry	Reactant	Condition(s)	Product	Yield
1^*a*^ ^,^^*b*^	**12**	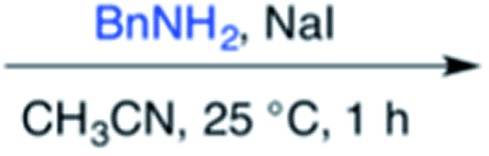	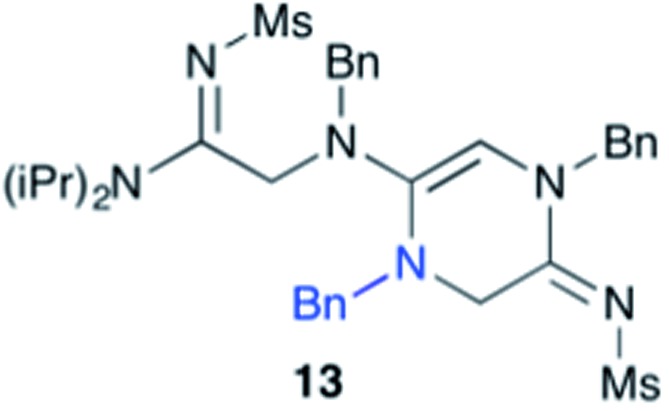	79%
2^*b*^	**12**	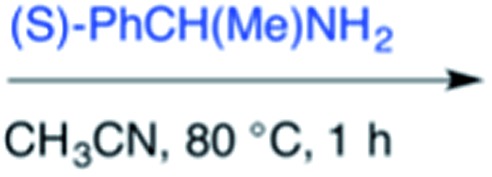	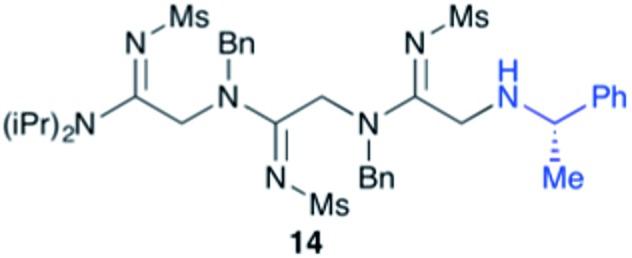	99%
3^*b*^	**12**	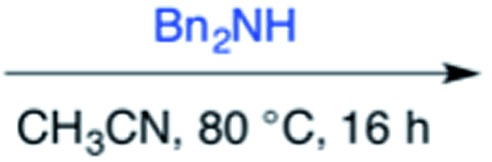	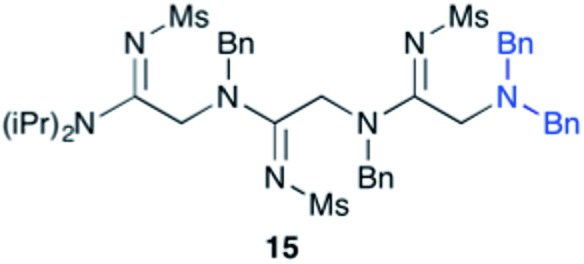	99%
4	**12**	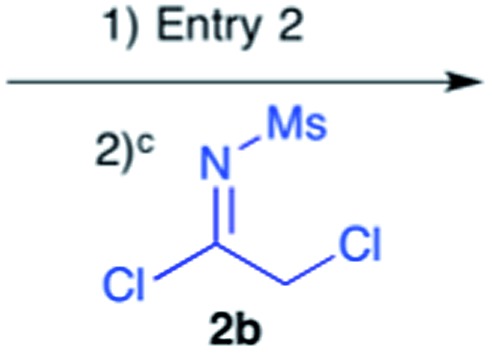	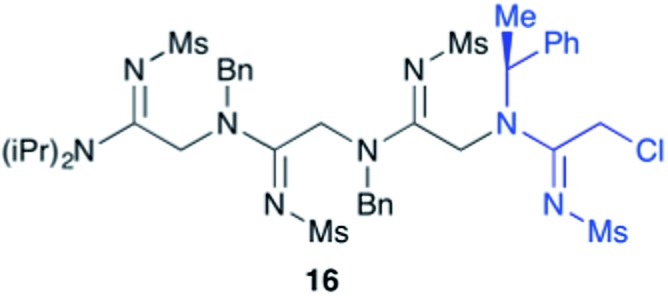	82%^*d*^

^*a*^5 equivalents of NaI were used.

^*b*^5 equivalents of amine were used.

^*c*^1.5 equivalents of imidoyl chloride and 3 equivalents of NMM were added to the corresponding amine in CH_3_CN at 60 °C for 3 hours.

^*d*^Two-step yield from **12**.

**Fig. 2:**
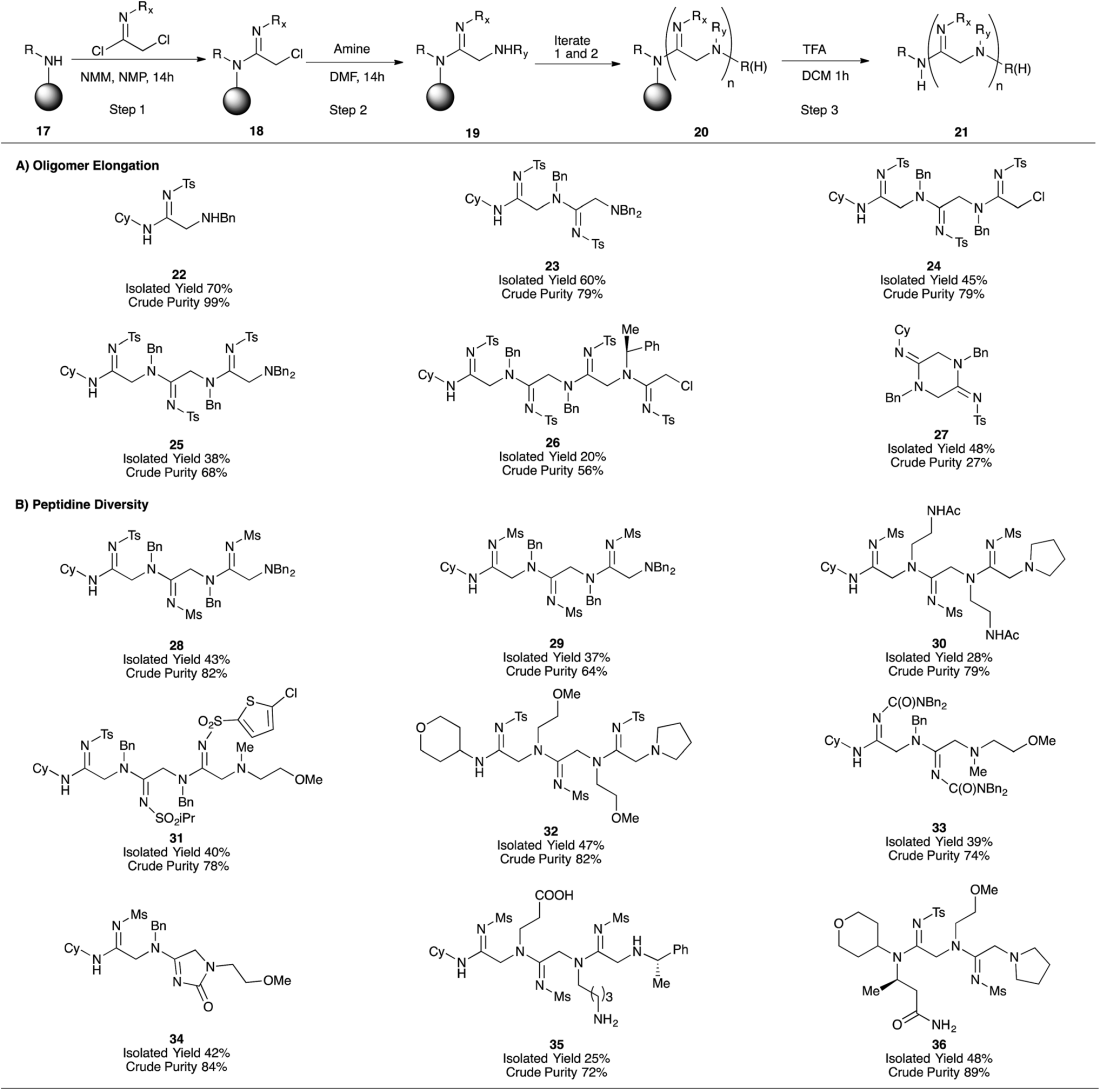
Solid phase synthesis of peptidine oligomers. Crude purity assessed by integration of the crude LC/MS spectrum at 254 nm (peptidine maximum absorbance). Values represent product peak area over total peak area. Rink MBHA resin (peptides international, 0.62 mmol g^–1^ or 0.51 mmol g^–1^) was used for synthesis.

Given the suitability of oligomer-based synthetic strategies for the preparation of one-bead-one-compound libraries,^18^ our next effort was focused on adapting our platform to the solid-phase. After developing a protocol for converting commercially-available Rink resin (**38**) to the corresponding cyclohexylamine derivative (**39**, ESI Fig. 3A†),^18^ we applied our iterative acylation-amination sequence (**17** → **21**) to the synthesis of peptidines containing between one and four monomeric units (Fig. 2). After optimizing the coupling of tosyl imidoyl chloride **3a** to the resin bead (ESI Fig. 3B†), we were then able to access linear peptidines **22–26** in good to excellent crude purities. Microcleavage of each resin-bound intermediate during the production of these peptidines followed by LC/MS analysis demonstrated that each synthetic step was capable of fully consuming each resin-bound starting material (for both amination and amidination steps).

Interestingly, the steric bulk of the resin-bound amine plays an important role in solid-phase syntheses by preventing on-resin cyclization and cleavage. Whereas peptidines containing N-terminal secondary amines tend to undergo spontaneous cyclo-deamination (*e.g.***12** → **13**, Table 2), analogous solid-supported peptidines do not, likely due to steric effects of the cyclohexyl and Rink-benzhydryl functional groups. This feature proved fortuitous in enabling the synthesis of 3- and 4-mers **24–26**. Upon resin cleavage, however, linear intermediates underwent rapid cyclization. Compound **27** is an example of such a situation, and was initially observed as a mixture with its uncyclized counterpart **43** (ESI Fig. 4†), accounting for the relatively low crude purity of this compound. Further cyclization occurred during purification upon exposure to silica, leading to an acceptable isolated yield.

After establishing the solid-phase elongation protocol, we explored the scope of *N*^1^ and *N*^2^ amidine substituents (Fig. 2B). In general, peptidines exclusively containing *N*^2^-sulfonyl substituents were produced in high purities and moderate isolated yields, even with widely varying *N*^1^-substituents (**28–32**, Fig. 2B). Notably, the synthesis of **32** was carried out on large scale (1 g of Rink resin), and provided 228 mg of pure product, demonstrating the scalability of our solid-phase platform. *N*^2^-carbamoyl and carbonyl substituents could also be incorporated into our solid-phase platform (**33** and **34**, respectively); however, as observed in analogous solution phase experiments, *N*^2^-carbamate derivative **34** readily underwent intramolecular cyclization to give an imidazolone substructure, similar to compound **5h**. Amines containing acid-labile side-chain protecting groups, similar to those employed in Fmoc SPPS (*e.g.*, Boc, ^*t*^Bu), were readily incorporated into peptidines, and unmasked after resin cleavage to give carboxylate and amine functional groups (as demonstrated by the synthesis of **35**). Employing homo-β-alanine as the C-terminal group allowed us to access 2-mer **36** in good yield and purity (Fig. 2B). Significant efforts to incorporate *N*^2^-aryl substituents into solid-phase oligomers proved unsuccessful, and yielded either unreacted, resin-bound starting material or complex mixtures. Taken together, these results indicate that a diverse range of *N*^1^ substituents, and electron-withdrawing *N*^2^ substituents are compatible with the solid-phase peptidine synthesis platform.

Given the unique structures of peptidines we next sought to evaluate their chemical and spectral properties. First, we observed that numerous peptidines (**11–12**, **14–15**, **23–26**, and **28–35**) tended to exhibit broadened peaks in ^1^H and ^13^C NMR spectra during routine characterization. This phenomenon was not surprising, as other oligomeric scaffolds, such as peptoids, can be very flexible and often exist in multiple different conformations at room temperature.^19^ Indeed, variable temperature NMR experiments performed on compound **12** (*d*_6_-DMSO, 25–125 °C) demonstrated peak sharpening with increasing temperatures (ESI Fig. 5†). These data – coupled with analytical HPLC results (ESI Appendix†) – support both the chemical purity of peptidines, while also indicating some degree of conformational flexibility at room temperature.

**Fig. 3:**
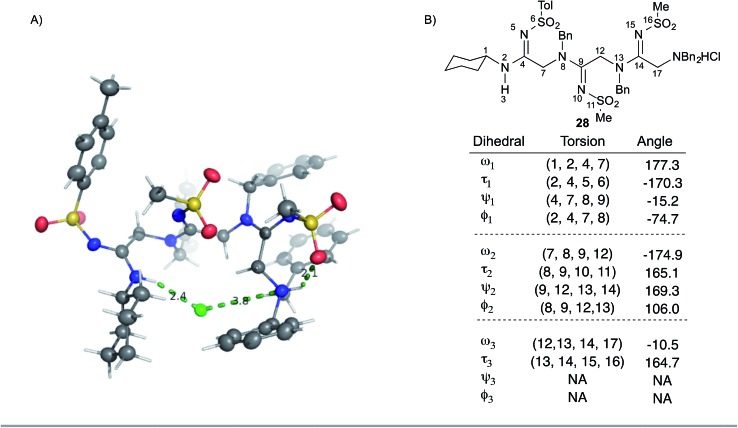
Peptidine structure and angle preferences. (A) Crystal structure of **28** in ORTEP model at the 50% confidence interval. Grey represents carbon, blue represents nitrogen, yellow represents sulfur, red represents oxygen, green represents chlorine, white represents hydrogen. Dashed lines represent hydrogen bonds or ionic interactions observed in crystal. (B) List of peptidine dihedral angles taken from the crystal structure of **28**. Atoms 6, 11, and 16 referenced to the sulfur atom.

We sought to gain additional insights into peptidine structure using X-ray crystallography. To this end, we obtained a crystal structure of **28** as the hydrochloride salt. As shown in Fig. 3A, **28** exists in a turn-like conformation, wherein the chloride counter-ion (green) interacts with atoms at both termini of the molecule. Additionally, a hydrogen bond between the terminal ammonium N–H group and the adjacent sulfonamide oxygen atom (Fig. 3A, right most dotted green line, 2.1 Å) locks the N-terminal residue into a H-bonded seven-membered ring. Examining **28** from an alternate perspective reveals that all *N*^2^-substituents are located opposite to the chloride ion (ESI Fig. 6†) while the *N*^1^-substituents project perpendicularly with respect to the peptidine backbone. This data suggests that the unique arrangement of H-bond donors and acceptors within peptidines exerts a significant impact on conformation and may impart distinct structural features that could prove useful for the design of peptidomimetics in future studies.

To gain further insight into peptidine structure, we analyzed crystallographic data in terms of standard peptide dihedral angles phi (*Φ*), psi (*ψ*), and omega (*ω*) (Fig. 3B). Additionally we also defined a new dihedral angle, called tau (*τ*), referring to the position of the *N*^2^-substituent relative to the *N*^1^-nitrogen (Fig. 3B). Perhaps most notably, all three *τ* angles exist in a *trans* conformation (*τ* = 180° ± 15°), despite each being in a different steric environment. This observation is consistent with previous literature reports,^20^–^22^ as well as computational studies (see below). The *ω* angles in **28**, on the other hand, proved slightly more variable, demonstrating a preference for either *cis* or *trans* conformations (*ω* = –10.5°, –174.9°, 177.3°), reminiscent of disubstituted amides found in peptides and peptoids.^23^ Due to these structural constraints (*ω* ≈ 180° or 0°; *τ* ≈ 180°), there is a high degree of co-planarity surrounding each amidine in **28** (Fig. 3A and B). The most flexible dihedrals in **28** were *Φ* and *ψ*, once again reminiscent of peptides and peptoids. Although in the case of **28**, we believe these angles are driven to accommodate the organizing N–H–Cl interactions, we are currently conducting investigations on the intrinsic preferences of these angles as a function of *N*^1^ and *N*^2^ substituent properties.

To understand the tendency of the amidine *N*^2^-substituent to prefer the *trans* geometry (*τ* = 180° ± 15°), we performed quantum chemical energy calculations (Table 3). Previous analysis of amidine geometries suggest that the *trans* geometry is favored in *N*^2^-alkyl and *N*^2^-aryl substituted amidines,^24^ with several examples of substituents preferring the *cis* geometry.^25^ However, to our knowledge, no calculations have been carried out to evaluate the conformational preferences of strongly electron withdrawing substituents at the *N*^2^ position such as sulfonyl or carbonyl groups. In evaluating the geometric preferences of the *N*^2^-substituent we utilized a simple monomer amidine as a model system. Thus, we employed 2D Sketcher and MacroModel software packages using the OPLS 2005 force field to construct and optimize six model structures including sulfonyl-, carbomethoxy-, carbamoyl-, and aryl-functionalized *N*,*N*-dimethylacetamidine derivatives (Table 3, entries 1–6). For each compound, we set initial *τ* angles to 0° and 180°, and performed geometry optimizations using the program Jaguar.^26^ Density functional theory (DFT) with the B3LYP functional and 6-31G** basis set were used in this process. Energies from minimized structures are presented in Fig. 3C. As expected, for each amidine tested, calculations predict a significant energetic preference for the *trans* geometry, ranging from 2–12 kcal mol^–1^. These results are consistent with crystallographic data for compound **28**, and suggest that, in general, the peptidines reported herein are all likely to exhibit a substantial preference for the *trans* geometry. Interestingly, other authors have observed that amidines containing electron-donating *N*^2^ substituents such as alkoxy substituents readily exist as *cis*/*trans* mixtures with preferences for the *cis* geometry.^27^,^28^ Although our synthesis platform is currently incompatible with such electron-rich groups on *N*^2^, the possibility exists that the *τ* geometry can be modulated through substituent effects. Additionally, we tested the hydrolytic stability of several compounds in PBS and observed minimal hydrolysis suggesting that peptidines may be suitable for use in biological contexts (ESI Fig. 7†).

**Table 3:** Calculations on tau angle preference

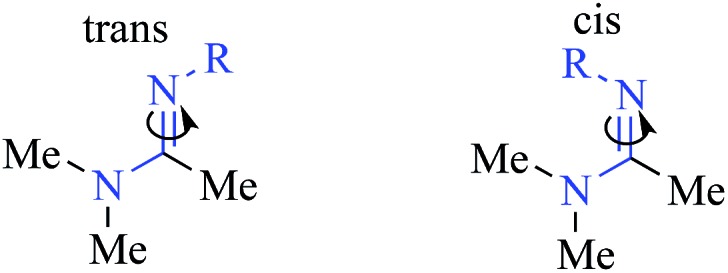			
Entry	R	Minimized *t* geometry (degrees)	ΔEnergy *trans*–*cis* (kcal mol^–1^)
1	S(O)_2_Tol	*trans*: –177.9	–11.6
		*cis*: –8.9	
2	S(O)_2_Me	*trans*: 178.2	–4.4
		*cis*: –16.2	
3	C(O)OMe	*trans*: 179.1	–2.0
		*cis*: 27.9	
4	C(O)NBn_2_	*trans*: –171.3	–4.1
		*cis*: –27.4	
5	Ph	*trans*: –179.4	–5.2
		*cis*: 17.4	
6	*p*-NO_2_Ph	*trans*: 177.0	–4.7
		*cis*: –20.8	

## Conclusions

Herein we have introduced a novel class of glycine-amidine-based oligomers, which we term “peptidines” readily afforded through modular synthetic approaches using both solid and solution phase chemistry. These synthetic protocols are high yielding, readily scalable, and enable straightforward installation of a variety of substituents onto backbone (*N*^1^) and amidine (*N*^2^) nitrogen atoms. Also, we have obtained a crystal structure of 3-mer **28**, which demonstrates all amidine motifs to adopt a *trans* geometry about the *τ* angle. This geometry conforms to structural constraints predicted by computational studies, and suggests that peptidines have the potential to project functionality in an ordered array. Furthermore, because peptidines possess two sites of diversity per monomeric unit (as opposed to only one present in peptides and peptoids), they may prove useful for library generation as greater diversity could be generated with shorter length oligomers. Peptidines therefore have significant potential to serve as useful tools for small molecule synthesis, peptidomimicry, and library generation.^29^–^31^ Further studies to explore peptidine structure and stability, and to apply this platform to the synthesis of a wide variety of functional molecules, are currently ongoing.

## Supplementary Material

Supplementary information

Crystal structure data
