# Supplementary material for: Peptidines: glycine-amidine-based oligomers for solution- and solid-phase synthesis
Source: Chem Sci. 2016 Feb 16;7(5):3317–24. doi: 10.1039/c5sc03882k (PMC6006957; doi:10.1039/c5sc03882k)
Supplement: Supplementary file 1 [file SC-007-C5SC03882K-s001.pdf]

Electronic Supplementary Material (ESI) for Chemical Science.
This journal is © The Royal Society of Chemistry 2016

Peptidines: Glycine-amidine-based oligomers for solution- and solid-phase synthesis

Julian Vastl, Rendy Kartika, Kichul Park, Art E. Cho, David A. Spiegel*
Department of Chemistry, Yale University, 225 Prospect Street, New Haven, CT 06520

Supporting Information

INDEX

1. General Procedures S-2
2. List of Abbreviations S-3
3. Amidine Nomenclature S-4
4. Supporting, Figures and Tables S-5
5. Solution-Phase Procedures S-8
6. Solid-Phase Procedures S-22
7. Calculations S-32
8. Crystallography S-33
9. References S-34
10 Catalogue of ^{1}H ^{13}C and Analytical HPLC traces S-35

1. General Procedure:

Starting materials were used as received unless otherwise noted. All reactions were performed in an inert, dry atmosphere of nitrogen in oven-dried glassware using anhydrous solvents unless noted otherwise. Reagent grade solvents were used for extractions and flash chromatography. Reaction progress was checked by analytical thin-layer chromatography (TLC, Merck silica gel 60 F-254 plates). The plates were then monitored with UV illumination. Purification was carried out on a Teledyne Isco CombiFlash Rf 200 using both silica gel (230-400 mesh) or C18. The solvent compositions reported for all chromatographic separations are on a volume/volume (v/v) basis. Infrared (IR) spectra were recorded on a Thermo Nicolet 6700 FT-IR Spectrometer. Proton and Carbon nuclear magnetic resonance (^{1}H or ^{13}C NMR) spectra were recorded on 400, 500, 600 or 800 MHz magnets from both Bruker and Varian. Chemical shifts are expressed in parts per million (δ scale) downfield from tetramethylsilane and are referenced to residual proton in the NMR solvent. Data are presented as follows: chemical shift, multiplicity (s = singlet, d = doublet, t = triplet, sept = septuplet, m = multiplet and/or multiple resonances), coupling constants in Hertz (Hz), and integration. Unless otherwise noted, ^{1}H NMR spectra are reported in parts per million (ppm) on the δ scale relative to $CDCl_3$ (δ 7.26) and ^{13}C NMR spectra are reported in parts per million (ppm) on the δ scale relative to $CDCl_3$ (δ 77.00). LC-MS analyses were performed on a Waters UPLC/MS instrument equipped with a RP-C18 column (1.7 μm particle size, 2.1x50 mm), dual atmospheric pressure chemical ionization (API)/electrospray(ESI) mass spectrometry detector, and photodiode array detector. Preparatory HPLC was performed with a SunFire Prep C18 OBD 10 μm 19x150 mm reverse-phase column as the stationary phase. Water and Acetonitrile both buffered with either 0.1% formic acid or 0.1% trifluoroacetic acid. The HPLC fractions were combined and lyophilized to give the corresponding peptidines as a freebase or salt depending on the presence of basic functional groups. Analytical HPLC was performed with a SunFire C18 5 μm 4.6x150 mm reverse-phase column as the stationary phase. Water and acetonitrile both buffered with 0.1% formic acid were used as the mobile phase.

2. List of Abbreviations:

Ac = Acetyl
Bn = Benzyl
Boc = tert-Butyloxylcarbonyl
Cy = Cyclohexyl
DCM = Dichloromethane
DIPA = Diisopropylamine
DMAP = Dimethylaminopyridine
DMF = Dimethylformamide
DMSO = dimethylsulfoxide
Et = Ethyl
FA = Formic acid
Fmoc = Fluorenylmethyloxycarbonyl
iPr = Isopropyl
IR = Infrared Radiation
LC/MS = Liquid chromatography/Mass Spectrometry
Me = Methyl
Ms = Mesyl
NMM = N-Methyl morpholine
NMP = N-Methyl pyrolidinone
NMR = Nuclear Magnetic Resonance
Ph = Phenyl
tBu = tert Butyl
SPPS = Solid Phase Peptide Synthesis
TFA = Trifluoracetic acid
THF = Tetrahydrofuran
TMB = trimethylbenzene
Tol = Toluyl
Ts = Tosyl

3. Amidine Nomenclature:

We use IUPAC numbering when referring to substituents off the amidine bonds[12]:

N^1 refers to the singly-bonded nitrogen in the amidine

N^2 Refers to the doubly-bonded nitrogen in the amidine

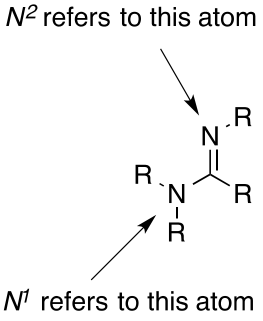

4. Supporting Figures and Tables:

SI Figure 1: N^2-Alkyl amidine synthesis

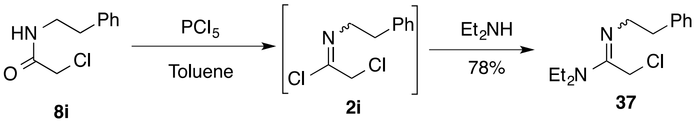

SI Figure 2: Cyclization of N^2-Carbamate Amidines

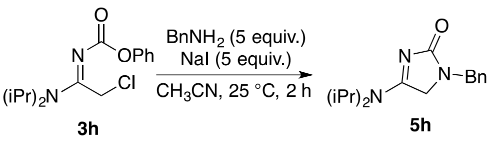

SI Figure 3: Resin Optimization

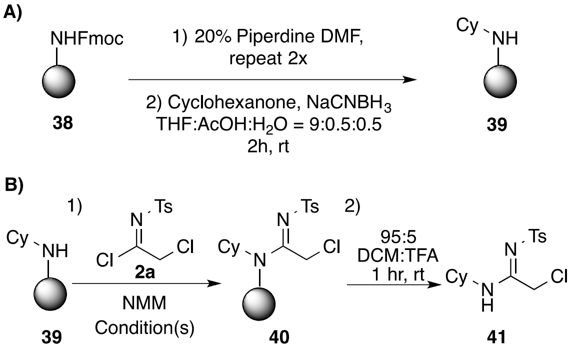

Entry	Condition	Isolated Yield
1	DCM, 3h	25%
2	NMP, 3h	70%
3[a]	DMAP, NMP 3h	-
4	NMP, O/N	90%

Reactions were run using 200 mg of resin and 4 mL of 0.2 M imidoyl chloride, 0.6 M of NMM. [a]20 mg of DMAP added to reaction vessel. A mixture of products was observed afterresin cleavage by LCMS and **41** was not isolated for this entry.

SI Figure 4: Cyclization to form 27.

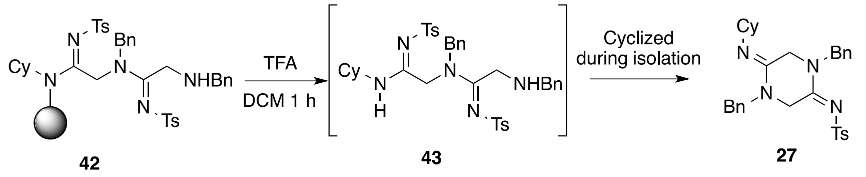

SI Figure 5: Variable Temperature NMR of 11 in *d6*-DMSO

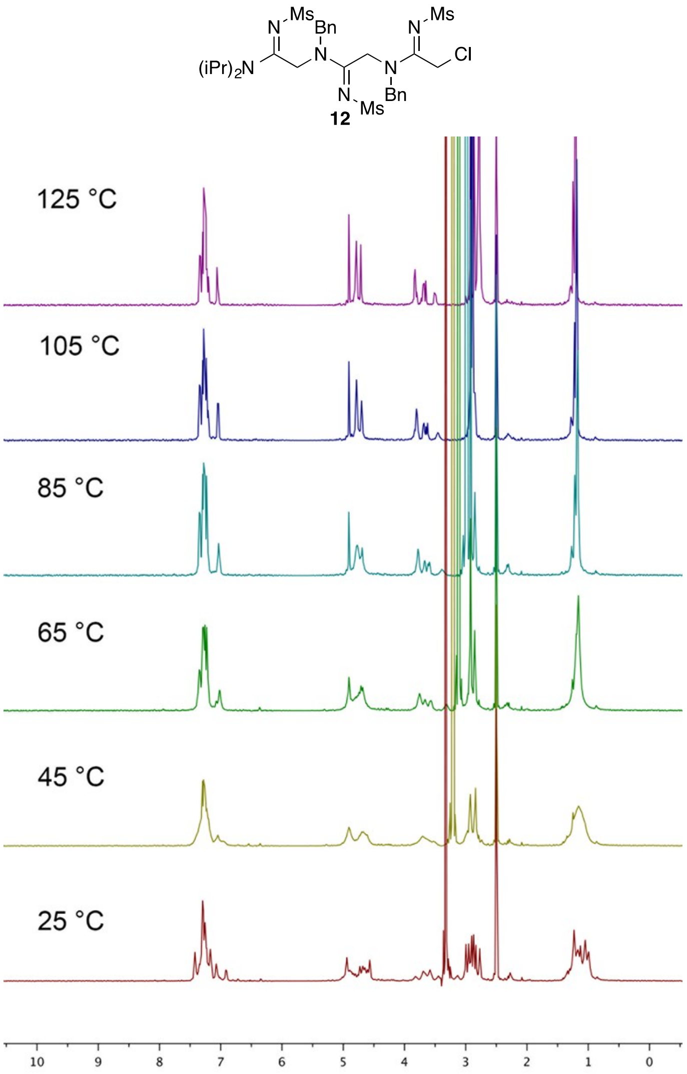

SI Figure 6: View of 28 Looking Down the Peptidine Axis.

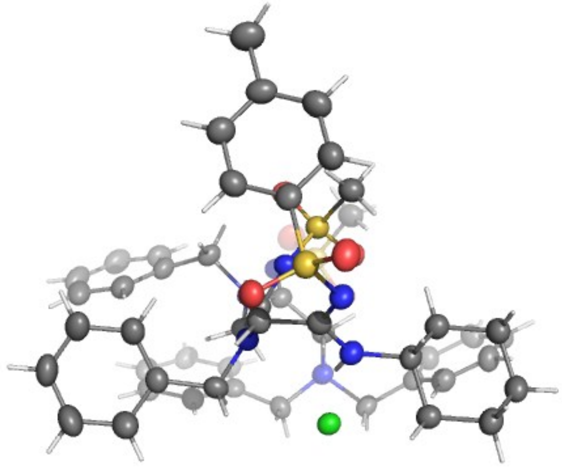

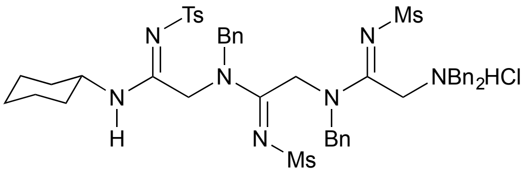

28

SI Figure 6: Crystal structure of **28** looking down peptidine axis with the C-terminus coming out of the page. The C-terminal cyclohexane is in the lower right corner. The chemical structure of **28** is shown on the right for clarity.

SI Figure 7: Stability of Peptidines in PBS

Entry	Compound	Observed Hydrolysis	Halflife
1	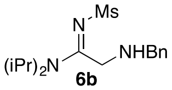 **6b**	No	No decomp observed (14 d)
2	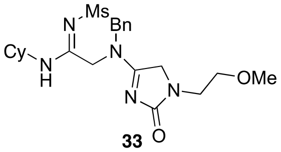 **33**	No	No decomp observed (14 d)
3	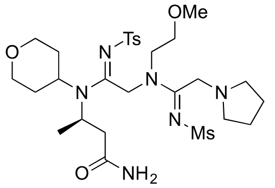 **35**	Yes	4.5 days

SI Figure 7: Peptidines were added from DMSO stocks into PBS (final conc. 100 uM) at 25° C with p-methylbenzhydrol as internal standard. Decomposition half-lives were measured by regular analysis by LCMS of the buffer followed by integrating the 254 nm channel of peptidine compared to internal standard. Samples were evaluated up to 14 days.

5. Solution Phase Procedures

Imidoyl chlorides, **2a-f,** used in Table 1, were synthesized immediately before use; procedures regarding the production and reactions these compounds can be found below (See procedures for 2a-f).

For the rest of this manuscript, imidoyl chlorides (2a-f) were prepared on large scale and stored as stock solutions. Stock solutions were prepared on 5-50 mmol scale stock stored in a bomb in a desiccator. Imidoyl chloride stock solutions were ~1M (0.85-1.05 M depending on batch) and ranged from 85-99% purity by ^{1}H NMR integration. The major byproduct was unreacted starting material for the spectra that were < 95% pure, which, in small quantities has no observable effect on aminidation procedures.

General Procedure for Imidoyl Chloride Stock Solution Preparation:
PCl_5 (11.45 g, 55 mmol, 1.1 equivalents) was added to 200 mL of anhydrous benzene containing α-Chloroacetamides **8a-h** (50 mmol) and was refluxed for 14 hours. The solution was allowed to cool, the solvent was removed by rotary evaporation at 40 °C to give several mL of a thick oil. The solution was allowed to sit on high vacuum for 24 hours to remove any volatile byproducts. Anhydrous DCM (44 mL for 50 mmol imidoyl chloride scale) was then added and the new stock solution was cannulated to a flame-dried bomb. 100 uL of the final solution was transferred to a 5 mL flame-dried flask and the solvent was removed by rotary evaporation. 5 uL of trimethylbenzene was added as an internal standard followed by ^{1}H NMR to calculate the final concentration of the imidoyl chloride. Stock solutions varied from 0.85-1.05 M in DCM. The bombs were kept in a dry-box stored over $CaSO_4$ and maintained purity for months. Production of **2a** is produced identically to the above procedure; production of **2b** is identical to above procedure except it is a white crystalline solid; production of **2c** and **2e** was prepared on 20 mmol scale; production of **2d** was carried out on the 5 mmol scale; production of **2f**, **2g**, and **2h** were carried out on the 10 mmol scale.

Procedure for the preparation of 2-chloro-*N*-tosylacetamide (8a):

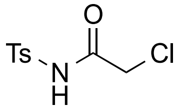

Chloroacetyl chloride (72 mL, 900 mmol) was added to *p*-toluenesulfonamide (51.4 g, 300 mmol) and allowed to reflux overnight (14 h) with vigorous stirring. After allowing the solution to cool to room temperature, 200 mL of hexanes was added and the reaction, while being capped to prevent the condensation of water, was transferred to a -20 °C freezer and after an hour precipitates were observed. The solution was then filtered, washed with hexanes (2 x 200 mL) and pentane (2 x 200 mL) to give 66.2 g of **2a** as a white crystalline solid in 89% yield. **^{1}H NMR** (500 MHz, Chloroform-*d*) δ 9.05 (s, 1H), 8.00-7.94 (m, 2H), 7.39-7.31 (m, 2H), 4.02 (s, 2H), 2.45 (s, 3H); **^{13}C NMR** (126 MHz, Chloroform-*d*) δ 163.99, 145.75, 134.62, 129.69, 128.62, 77.25, 77.00, 76.75, 42.20, 21.70; **IR** 1716, 1447, 1350, 1170, 1087, 860, 660, 550 cm^{-1}; **HRMS** Exact Mass ($C_9H_{10}ClNO_3S$ + H): calculated = 247.0070, measured = 247.0145.

Procedure for the preparation of 2-chloro-*N*-(methylsulfonyl)acetamide (8b):

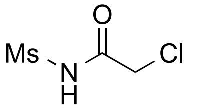

Chloroacetyl chloride (75 mL, 943 mmol) was added methanesulfonamide (30 g, 315 mmol) and allowed to reflux overnight (14 h) with vigorous stirring. The reaction, while being capped to prevent the condensation of water, was then transferred to a -20° C freezer for 30 minutes causing the product to precipitate. The reaction was filtered, washed with toluene (2 x 200 mL) and hexane (2 x 200 mL). This filtration process was carried out as fast as possible to prevent contamination with atmospheric water. The residual solid was then exposed to high vacuum for 4 hours to remove any residual hydrogen chloride to give 49.9 g of a white crystalline solid in 92% yield. Due to the hydroscopic nature of **8b** it was stored in a sealed container over calcium sulfate. **^{1}H NMR** (400 MHz, Chloroform-*d*) δ 8.70 (s, 1H), 4.15 (s, 2H), 3.35 (s, 3H); **^{13}C NMR** (126 MHz, Chloroform-*d*) δ 165.32, 42.40, 41.78; **IR** 3230, 3174, 1716, 1477, 1443, 1404, 1334, 1176, 1129, 997, 980, 884, 790, 554, 512 cm^{-1}; **HRMS** Exact Mass ($C_3H_6ClNO_3S$ + H): calculated = 171.9830, measured = 171.9882.

Procedure for the preparation of 2-chloro-*N*-(isopropylsulfonyl)acetamide (8c):

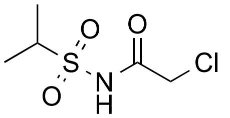

Chloroacetyl chloride (11.9 mL, 150 mmol) was added to isopropylsulfonamide (6.15 g, 50 mmol) and allowed to reflux overnight (14 h) with vigorous stirring. After allowing the solution to cool to room temperature, and an off-white precipitate was observed. The solution was then filtered, and the solid was washed extensively with hexanes (6 x 100 mL) providing a white crystalline solid. This material was exposed to high vacuum for 4 hours in order to remove any residual hydrogen chloride to provide 9.33 g of **8c** in 93.5% yield. **^{1}H NMR** (400 MHz, Acetone-d_6) δ 4.30 (s, 2H), 3.72 (h, *J* = 6.9 Hz, 1H), 1.36 (d, *J* = 6.9 Hz, 6H); **^{13}C NMR** (101 MHz, Acetone-d_6) δ 166.32, 54.29, 43.44, 16.00; **IR** = 1720, 1446, 1402, 1335, 887, 517 cm^{-1}; **HRMS** Exact Mass ($C_5H_{10}ClNO_3S$ + H): calculated = 200.0143, measured = 200.0192.

Procedure for the preparation of 2-chloro-*N*-((5-chlorothiophen-2-yl)sulfonyl)acetamide (8d):

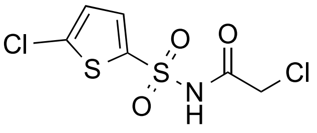

8d was prepared with modification of a previously published method[1]. 5-Chlorothiophene-2-sulfonamide (5 g, 25.3 mmol) was added to isopropyl acetate (56 mL), triethylamine (3.8 mL, 63.3 mmol) and 4-dimethylaminopyridine (20.8 mg 0.253 mmol). This solution was heated to 55° C and a solution of chloroacetylchloride (2.21 mL 27.8 mmol) in toluene (18.5 mL) was added drop-wise over an hour. The reaction was allowed to stir for another hour followed by the addition of water (30 mL) and 1 M HCl (30 mL). The reaction was then cooled, the organic layer was then separated and the aqueous phase was washed 2x 75 mL with isopropyl acetate. The combined organic layers were dried over sodium sulfate and concentrated by vacuum to provide 5.47 g of **8d** in 79% yield.**^{1}H NMR** (400 MHz, Acetone-d_6) δ 7.72 (d, *J* = 4.1 Hz, 1H), 7.21 (d, *J* = 4.1 Hz, 1H), 4.30 (s, 2H); **^{13}C NMR** (101 MHz, Acetone-d_6) δ 165.67, 139.08, 138.38, 135.58, 128.40, 43.47; **IR** = 1703, 1407, 1366, 1148, 1091, 1040, 996, 870, 798, 609, 562, 535 cm^{-1}; **HRMS** Exact Mass ($C_6H_5Cl_2NO_3S$ + H): calculated = 273.9161, measured = 273.9178.

Procedure for the preparation of 2-chloro-*N*-phenylacetamide (8e):

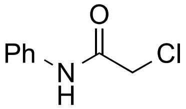

8e was prepared by the procedure in the following reference. Proton spectra matched published values.[2]

Procedure for the preparation of 2-chloro-*N*-(4-nitrophenyl)acetamide (8f):

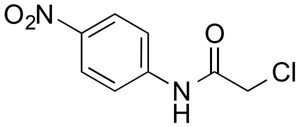

8f was prepared by the procedure in the following reference. Proton spectra matched published values. [3]

Procedure for the preparation of phenyl (2-chloroacetyl)carbamate (8g):

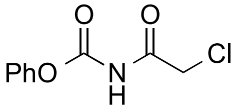

Oxalyl chloride (4.3 mL, 50 mmol) was added to a stirring suspension of chloroacetamide (4.68g, 50 mmol) in anhydrous dichloroethane (200 mL, dried over 4Å molecular sieves overnight). The reaction was then heated to a vigorous reflux for 2 hours. The reaction was then cooled to room temperature, followed by the addition of phenol (4.7 g, 50 mmol). The reaction was then heated to 50 °C and allowed to stir overnight (14 h). The solvent was removed by vacuum and the residual white solid was recrystallized in 30-40 mL of boiling toluene. The white solid was washed with 3 x 150 mL of pentane and dried under high vacuum to provide 8.8 g of **8g** in 82% yield. **^{1}H NMR** (500 MHz, Acetone-d_6) δ 10.24 (s, 1H), 7.44 (d, J = 8.1 Hz, 2H), 7.29 (d, J = 7.6 Hz, 1H), 7.21 (d, J = 7.9 Hz, 2H), 4.66 (s, 2H); **^{13}C NMR** (126 MHz, Acetone-d_6) δ 166.86, 150.50, 150.28, 129.49, 126.13, 121.59, 44.16, 44.14; **IR** = 1770, 1527, 1482, 1220, 1149, 976, 817, 687, 539 cm^{-1}; **HRMS** Exact Mass ($C_9H_8ClNO_3$ + H): calculated = 214.0193, measured = 214.0187.

Procedure for the preparation of 2-chloro-*N*-(dibenzylcarbamoyl)acetamide (8h):

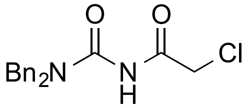

Oxalyl chloride (10.4 mL, 120 mmol) was added to a stirring suspension of chloroacetamide (11.2 g 120 mmol) in anhydrous dichloroethane (450 mL, dried over 4Å molecular sieves overnight). The reaction was then heated to reflux overnight (14 h). The reaction was cooled to room temperature, followed by the addition of dibenzylamine (23.2 mL, 120 mmol). The reaction was stirred for 2 hours at room temperature and was then transferred to a separatory funnel, and the organic phase was washed with 300 mL of 1 M HCl twice. The organic phase was then dried over sodium sulfate and removed under vacuum to provide 32.1 g of **8h** in 84.7% yield. **^{1}H NMR** (400 MHz, Chloroform-d) δ 8.01 (s, 1H), 7.44-7.31 (m, 5H), 5.21 (s, 2H), 4.47 (s, 2H); **^{13}C NMR** (100 MHz, Chloroform-d) δ 166.46, 150.95, 134.43, 128.91, 128.74, 128.57, 68.47, 43.55; **IR** = 3219, 1716, 1662, 1527, 1427, 1235, 1207, 1138, 963, 755, 739, 503 cm^{-1}; **HRMS** Exact Mass ($C_{17}H_{18}ClN_2O_2$ + H): calculated = 317.1052, measured = 317.0973.

Procedure for the preparation of 2-chloro-*N*-phenethylacetamide (8i):

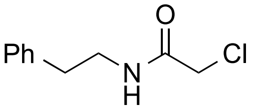

8i was prepared by the procedure in the following reference. Proton spectra matched published values. [3]

Procedure for the preparation of (*E*)-2-chloro-*N*,*N*-diisopropyl-*N*' tosylacetimidamide (3a):

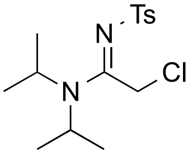

2a (247 mg, 1 mmol) was dissolved in benzene (4 mL) and PCl_5 (228 mg, 1.1 mmol) was added. The reaction was refluxed under nitrogen for 1 hour. The solvent was then removed by rotary evaporation, producing a pale-yellow oil, which was then exposed to high vacuum for 30 minutes to remove any volatile byproducts. Dichloromethane (10 mL) was then added, the reaction was cooled to 0 °C by an ice bath, followed by the drop-wise addition of diisopropylamine (1.4 mL, 10 mmol). The ice bath was then removed and the reaction was allowed to stir for 2 hours at room temperature. The solvent was removed by vacuum and the residual material was directly loaded onto a silica gel column and purified using an automated purification system (Teledyne Isco, Inc.) on a normal phase column using a 0-40% gradient of ethyl acetate in hexanes to provide 307 mg of **5a** in 93% yield. **^{1}H NMR** (500 MHz, Chloroform-*d*) δ 7.81 (d, *J* = 8.2 Hz, 2H), 7.25 (d, *J* = 8.4 Hz, 2H), 4.72 (s, 2H), 4.17 (sept, *J* = 6.6 Hz, 1H), 3.55 sept, *J* = 6.8 Hz, 1H), 2.39 (s, 3H), 1.31 (d, *J* = 6.8 Hz, 6H), 1.26 (d, *J* = 6.6 Hz, 6H); **^{13}C NMR** (126 MHz, Chloroform-*d*) δ 158.40, 141.96, 140.69, 129.08, 126.16, 50.77, 48.40, 36.40, 21.42, 20.37, 19.54; **IR** = 1553, 1440, 1473, 1261, 1132 1078, 938, 786, 723, 548 cm^{-1}; **HRMS** Exact Mass ($C_{15}H_{23}ClN_2O_2S$+ H): calculated = 331.1242, measured = 331.1266.

Procedure for the preparation (*E*)-2-chloro-*N*,*N*-diisopropyl-*N*'-(methylsulfonyl)-acetimidamide (3b):

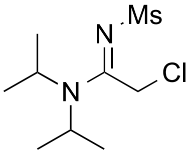

3b was synthesized under analogous identically to **3a**. The crude material was purified using an automated purification system (Teledyne Isco, Inc.) on a normal phase column using a 0-5% gradient in methanol in dichloromethane to provide 219 mg of **3b** a 86% yield. **^{1}H NMR** (400 MHz, Chloroform-*d*) δ 4.60 (s, 2H), 4.15 (sept, *J* = 6.6 Hz, 1H), 3.60 sept, *J* = 6.7 Hz, 1H), 3.01 (d, *J* = 0.5 Hz, 3H), 1.43 (d, *J* = 6.8 Hz, 6H), 1.26 (d, *J* = 6.6 Hz, 6H); **^{13}C NMR** (100 MHz, Chloroform-*d*) δ 158.79, 50.67, 48.26, 43.27, 36.80, 20.31, 19.55; **IR** = 1559, 1374, 1269, 1113, 1054, 942, 805, 516 cm^{-1}; **HRMS** Exact Mass ($C_9H_{19}ClN_2O_2S$ + H): calculated = 255.0929, measured = 255.0942.

Procedure for the preparation of (*E*)-2-chloro-*N*,*N*-diisopropyl-*N*'-(isopropylsulfonyl)-acetimidamide (3c):

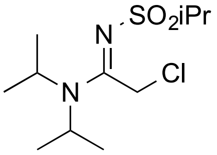

3c was synthesized and purified under analogous conditions to **3a** to give 222 mg of **3c** in 88% yield. **^{1}H NMR** (500 MHz, Chloroform-*d*) δ 4.61 (s, 2H), 4.14 (sept, *J* = 6.6 Hz, 1H), 3.62-3.53 (m, 1H), 3.10 (sept, *J* = 6.8 Hz, 1H), 1.39 (d, *J* = 6.9 Hz, 12H), 1.35 (d, *J* = 6.8 Hz, 6H), 1.22 (d, *J* = 6.6 Hz, 6H); **^{13}C NMR** (126 MHz, Chloroform-*d*) δ 159.78, 54.27, 50.56, 47.91,

37.06, 20.25, 19.51, 16.39; **IR** = 1557, 1451, 137, 1289, 1248, 1108, 1058, 943, 795, 735 cm^{-1}; **HRMS** Exact Mass ($C_{11}H_{23}ClN_2O_2S$ + H): calculated = 283.1242, measured = 283.1247.

Procedure for the preparation of (*E*)-2-chloro-*N*'-((5-chlorothiophen-2-yl)sulfonyl)-*N*,*N*-diisopropylacetimidamide (3d):

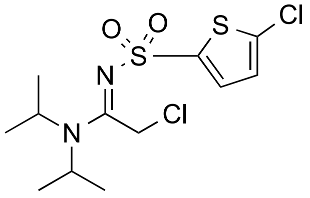

3d was synthesized and purified under conditions analogous conditions to **3a** to give 313 mg of **3d** in 88% yield.**^{1}H NMR** (400 MHz, Chloroform-*d*) δ 7.41 (d, *J* = 4.0 Hz, 1H), 6.85 (d, *J* = 4.0 Hz, 1H), 4.67 (s, 2H), 4.19 (sept, *J* = 6.6 Hz, 1H), 3.70-3.58 (m, 1H), 1.42 (d, *J* = 6.8 Hz, 6H), 1.30 (d, *J* = 6.6 Hz, 6H); **^{13}C NMR** (100 MHz, Chloroform-*d*) δ 158.74, 143.02, 135.42, 129.09, 129.08, 129.06, 125.82, 51.18, 48.79, 36.36, 20.29, 19.56, 19.54; **IR** = 1555, 1416, 1375, 1295, 1129, 1080, 989, 792, 617, 538 = cm^{-1}; **HRMS** Exact Mass ($C_{12}H_{18}Cl_2N_2O_2S_2$ + H): calculated = 357.0260, measured = 357.0271.

Procedure for the preparation of (*E*)-2-chloro-*N*,*N*-diisopropyl-*N*'-(4-nitrophenyl)-acetimidamide (3e):

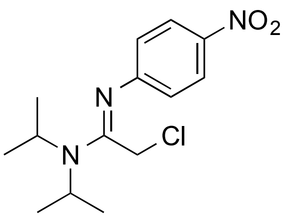

3e was synthesized under analogous conditions to **3a** with the following changes. Reaction was run at 2 mmol scale and was purified using an automated purification system (Teledyne Isco, Inc.) on a normal phase column using a 0-10% gradient of methanol in dichloromethane to provide 430 mg of **3e** a 72% yield. **^{1}H NMR** (500 MHz, Methylene Chloride-*d*$_2$) δ 8.07-7.99 (m, 2H), 6.77-6.70 (m, 2H), 3.86 (s, 3H), 3.81-3.74 (m, 2H), 1.29 (d, *J* = 6.9 Hz, 12H); **^{13}C NMR** (126 MHz, CD_2Cl_2) δ 157.61, 151.71, 142.39, 125.57, 122.47, 36.66, 20.73; **IR** = 1610, 1575, 1499, 1329, 1277, 1142, 1106, 855, 762 cm^{-1}; **HRMS** Exact Mass (C14H20ClN3O2+ H): calculated = 298.1317, measured = 298.1323.

Procedure for the preparation of (*E*)-2-chloro-*N*,*N*-diisopropyl-*N*'-phenylacetimidamide (3f):

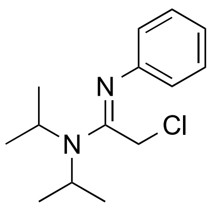

3f was synthesized under analogous conditions to **3a** with the following changes. Chlorination with PCl_5 was run at room temperature for 3 hours, and the treatment with diisopropylamine was allowed to stir overnight (14 h). **3f** was purified using an automated purification system (Teledyne Isco, Inc.) on a normal phase column using a 0-40% gradient of ethyl acetate in hexanes to provide 176 mg of **3f** in 70% yield. **^{1}H NMR** (400 MHz, Chloroform-*d*) δ 7.34-7.22 (m, 2H), 7.01-6.91 (m, 1H), 6.84-6.76 (m, 2H), 3.96 (s, 2H), 3.89-3.76 (m, 2H), 1.41 (d, *J* = 6.8 Hz, 12H); **^{13}C NMR** (101 MHz, Chloroform-*d*) δ 150.78, 150.61, 128.81, 121.77, 121.24, 53.40, 47.61, 35.38, 20.58; **IR** = 1609, 1588, 1456, 1442, 1342, 1250, 1142, 1043 805, 768, 697, 599, 493 cm^{-1}; **HRMS** Exact Mass ($C_{14}H_{21}ClN_2$ + H): calculated = 253.1467, measured = 253.1472.

Procedure for the preparation of phenyl (*E*)-(2-chloro-1-(diisopropylamino)-ethylidene)carbamate (3g):

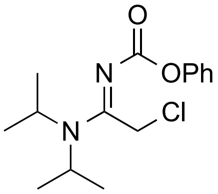

3g was synthesized under similar conditions to **3a** with the following changes. Chlorination was allowed to stir overnight (14 hours) before removal of solvent. The crude product was purified using an automated purification system (Teledyne Isco, Inc.) on a normal phase column using a 0-50% gradient of ethyl acetate in hexanes to provide 233 mg of **3g** in 79 % yield. **^{1}H NMR** (501 MHz, Chloroform-*d*) δ 7.44 (t, *J* = 7.9 Hz, 2H), 7.28-7.21 (m, 3H), 4.62 (s, 2H), 4.25-4.19 (m, 1H), 3.83-3.75 (m, 1H), 1.39 (d, *J* = 8.9 Hz, 12H); **^{13}C NMR** (126 MHz, Chloroform-*d*) δ 161.63, 159.46, 152.21, 128.88, 124.67, 121.77, 50.10, 48.13, 35.50, 20.53, 19.26; **IR** = 1678, 1560, 1482, 1445, 1374, 1246, 1188, 1161, 1153, 1051, 980, 761, 692, 576, cm^{-1}; **HRMS** Exact Mass ($C_{15}H_{21}ClN_2O_2$ + H): calculated = 297.1364, measured = 297.1354.

Procedure for the preparation of (*E*)-2-chloro-*N'*-(dibenzylcarbamoyl)-*N*,*N*-diisopropyl-acetimidamide (3h):

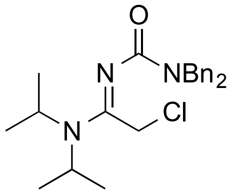

3h was synthesized under identical conditions as **3g** to give 297 mg of **3h** in 83% yield. **^{1}H NMR** (500 MHz, Chloroform-*d*) δ 7.37-7.22 (m, 10H), 4.57 (s, 4H), 4.52 (s, 2H), 4.11-4.07 (m, 1H), 3.76-3.48 (m, 1H), 1.33-1.28 (m, 12H); **^{13}C NMR** (126 MHz, Chloroform-*d*) δ 163.39, 158.27, 138.48, 138.33, 128.39, 128.35, 127.92, 127.42, 126.91, 126.88, 50.07, 48.20, 35.85, 20.57; **IR** = 1592, 1579, 1453, 1346, 1197, 1150, 1096, 742, 699, 613 cm^{-1}; **HRMS** Exact Mass ($C_{23}H_{30}ClN_3O$ + H): calculated = 400.2151, measured = 400.2145.

Procedure for the preparation of (*E*)-2-(benzylamino)-*N*,*N*-diisopropyl-*N'*-tosyl-acetimidamide (5a):

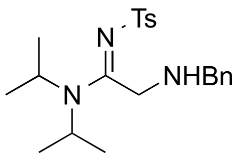

3a (165 mg, 0.5 mmol) was dissolved in 5 mL of a 0.5 M sodium iodide solution in acetonitrile. To this, benzyl amine (273 uL, 2.5 mmol) was added and the reaction was allowed to stir for 2 hours. The solution was removed by rotary evaporation, and the crude mixture placed onto a silica gel column. The product was purified using an automated organic purification system (Teledyne Isco, Inc.) on a normal phase column using a 0-10% gradient of methanol in dichloromethane to provide 385 mg of **5a** in 96% yield. **^{1}H NMR** (400 MHz, Chloroform-*d*) δ 7.84-7.76 (m, 2H), 7.39-7.21 (m, 7H), 4.04 (sept, *J* = 6.7 Hz, 1H), 3.82 (s, 2H), 3.75 (s, 2H), 3.50 (m, 1H), 2.40 (s, 3H), 1.31 (d, *J* = 6.8 Hz, 6H), 1.11 (d, *J* = 6.6 Hz, 6H); **^{13}C NMR** (101 MHz, Chloroform-*d*) δ 163.62, 141.61, 141.48, 139.28, 129.06, 128.70, 128.38, 128.26, 127.25, 126.00, 54.12, 49.99, 49.41, 47.89, 21.45, 20.31, 19.71; **IR** = 1524, 1447, 1370, 1267, 1134, 1082, 1051, 807, 701, 548 cm^{-1}; **HRMS** Exact Mass ($C_{22}H_{31}N_3O_2S$+ H): calculated = 402.2210, measured = 402.2221.

Procedure for the preparation of (*E*)-2-(benzylamino)-*N*,*N*-diisopropyl-*N'*-(methylsulfonyl)acetimidamide (5b):

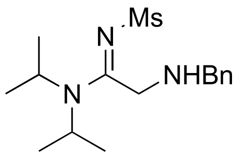

3b (165 mg, 1.6 mmol) was dissolved in 16 mL of a 0.5 M sodium iodide solution in acetonitrile. To this, benzyl amine (921 uL, 8.35 mmol) was added and the reaction was allowed to stir for 2 hours. The solution was removed by rotary evaporation, and the crude mixture was placed onto a silica gel column. The product was purified using an automated purification system (Teledyne Isco, Inc.) on a normal phase column using a 0-5% gradient of methanol in dichloromethane to provide 540 mg of **5b** in 95% yield. **^{1}H NMR** (400 MHz, Chloroform-*d*) δ 7.38-7.20 (m, 5H), 3.99 (hept, *J* = 13.2, 6.7 Hz, 1H), 3.80 (s, 2H), 3.71 (s, 2H), 3.56-3.51 (m, 1H), 3.00 (s, 2H), 1.41 (d, *J* = 6.8 Hz, 6H), 1.10 (d, *J* = 6.6 Hz, 6H); **^{13}C NMR** (126 MHz, Chloroform-*d*) δ 163.82, 139.31, 128.65, 128.23, 127.21, 54.08, 49.78, 49.55, 47.73, 43.59, 20.33, 19.92; **IR** = 1551, 1370, 1268, 1123, 1054, 966, 811, 515 cm^{-1}; **HRMS** Exact Mass ($C_{16}H_{27}N_3O_2S$+ H): calculated = 326.1897, measured = 326.1907.

Procedure for the preparation of (*E*)-2-(benzylamino)-*N*,*N*-diisopropyl-*N*'-(isopropylsulfonyl)acetimidamide (5c):

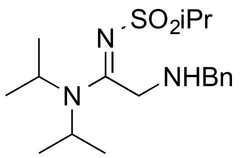

3c (58 mg, 0.20 mmol) was dissolved in 2 mL of a 0.5 M sodium iodide solution in acetonitrile. To this, benzyl amine (111 uL, 1 mmol) was added and the reaction was allowed to stir for 2 hours. The solution was removed by rotary evaporation, and the crude mixture placed onto a silica gel column. The product was purified using an automated organic purification system (Teledyne Isco, Inc.) on a normal phase column using a gradient of 0-60% ethyl acetate in hexanes to provide 66 mg of **5c** in 92% yield. **^{1}H NMR** (500 MHz, Chloroform-*d*) δ 7.37-7.21 (m, 5H), 4.01 (hept, *J* = 6.7 Hz, 1H), 3.81 (s, 2H), 3.74 (s, 2H), 3.58-3.52 (m, 1H), 3.13 (hept, *J* = 6.8 Hz, 1H), 2.53-2.49 (m, 1H), 1.41 (m, 12H), 1.10 (d, *J* = 6.6 Hz, 6H); **^{13}C NMR** (126 MHz, Chloroform-*d*) δ 164.85, 139.28, 128.65, 128.14, 127.12, 54.37, 54.04, 49.69, 47.43, 20.28, 19.93, 16.61; **IR** = 1549, 1370, 1249, 1106, 1056, 804, 758, 704 cm^{-1}; **HRMS** Exact Mass ($C_{18}H_{31}N_3O_2S$ + H): calculated = 354.2210, measured = 354.2216.

Procedure for the preparation of (*E*)-2-(benzylamino)-*N*'-((5-chlorothiophen-2-yl)sulfonyl)-*N*,*N*-diisopropylacetimidamide (5d):

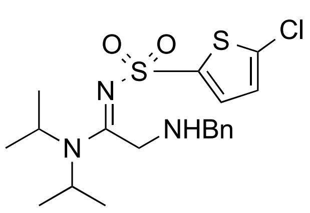

3d (53 mg, 0.20 mmol) was dissolved in 1.4 mL of a 0.5 M sodium iodide solution in acetonitrile. To this, benzyl amine (76 uL, 0.7 mmol) was added and the reaction was allowed to stir for 2 hours. The solution was removed by rotary evaporation, and the crude mixture was loaded onto a silica gel column. The product was purified using an automated purification system (Teledyne Isco, Inc.) on a normal phase column using a 0-40% gradient of ethyl acetate in hexanes to provide 63 mg of **5d** in 99% yield. **^{1}H NMR** (400 MHz, Chloroform-*d*) δ 7.31-7.15 (m, 6H), 6.77 (d, *J* = 4.0 Hz, 1H), 3.97 (sept, *J* = 6.7 Hz, 1H), 3.75 (s, 2H), 3.66 (s, 2H), 3.49 (m, 1H), 1.32 (d, *J* = 6.8 Hz, 6H), 1.05 (d, *J* = 6.6 Hz, 6H); **^{13}C NMR** (101 MHz, Chloroform-*d*) δ 163.76, 143.89, 139.00, 135.02, 129.65, 128.71, 128.48, 128.32, 127.36, 125.80, 54.07, 50.40, 49.56, 48.27, 20.26, 19.73; **IR** = 1548, 1453, 1417, 1372, 1280, 1124, 1078, 1053, 988, 801, 626, 537 cm^{-1}; **HRMS** Exact Mass ($C_{19}H_{26}ClN_3O_2S_2$ + H): calculated = 428.1228, measured = 428.1224.

Procedure for the preparation of (*E*)-2-(benzylamino)-*N*,*N*-diisopropyl-*N*'-(4-nitrophenyl)acetimidamide (5e):

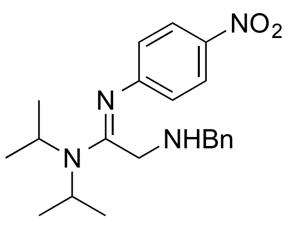

3e (420 mg, 1.4 mmol) was dissolved in 14 mL of a 0.5 M sodium iodide solution in acetonitrile. To this, benzyl amine (780 uL, 7.1 mmol) was added and the reaction was allowed to stir for 2 hours. The solution was removed by rotary evaporation, and the crude mixture was redissolved in ~5 mL of a 1:1:0.01 water/acetonitrile/TFA solution for reverse phase purification. The product was purified using an automated purification system (Teledyne Isco, Inc.) on a reverse phase C-18 column using a 0-75% gradient of acetonitrile in water containing 0.1% TFA. The product eluted at ~65% acetonitrile. The fractions were combined, rotovaped, and azeotroped with ethanol and redissolved in 20 mL of dichloromethane. The dichloromethane solution was washed once with a saturated sodium carbonate solution, then dried over sodium sulfate, the solvent was removed to provide 470 mg of **5e** in 90% yield. **^{1}H NMR** (400 MHz, Chloroform-*d*) δ 8.04-7.93 (m, 2H), 7.30-7.15 (m, 3H), 7.12-7.03 (m, 2H), 6.58-6.47 (m, 2H), 4.62 (s, 2H), 4.10 (s, 2H), 3.74 (hept, *J* = 7.0 Hz, 2H), 1.24 (d, *J* = 6.8 Hz, 12H); **^{13}C NMR** (101 MHz, Chloroform-*d*) δ 161.38, 159.40, 153.11, 138.86, 135.40, 129.07, 127.81, 126.59, 126.04, 111.74, 54.85, 53.89, 47.69, 20.60; **IR** = 1659, 1593, 1495, 1307, 1112, 730, 650 cm^{-1}; **HRMS** Exact Mass ($C_{21}H_{28}N_4O_2$ + H): calculated = 369.2286, measured = 369.2294.

Procedure for the preparation of (*E*)-2-(benzylamino)-*N*,*N*-diisopropyl-*N*'-phenylacetimidamide hydrochloride (5f):

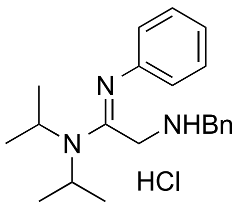

3f (50 mg, 0.15 mmol) was dissolved in 1.5 mL of a 0.5 M sodium iodide solution in acetonitrile. To this, benzyl amine (85 uL, 0.77 mmol) was added and the reaction was allowed to stir for 2 hours. The solution was removed by rotary evaporation, and the residual solid was immediately resolvated in ~2-3 mL of dichloromethane and loaded onto a silica gel column. The crude mixture was then purified using an automated purification system (Teledyne Isco, Inc.) on a normal phase column using a 0-30% gradient of ethyl acetate in hexanes. The fractions containing product were immediately combined and 2 M HCl in ether (~200 uL, 0.30 mmol, 2 equiv.) was added causing a white precipitate to form. The residual solvent was removed to provide 65 mg of **5f** in 91% yield assuming a single HCl salt. Previous attempts to isolate and purify this compound without salting the amine lead complex decomposition before we were able to obtain characterization-quality NMR spectra. **^{1}H NMR** (500 MHz, Methanol-*d*$_4$) δ 7.70-7.63 (m, 2H), 7.62-7.56 (m, 3H), 7.43-7.28 (m, 5H), 4.42 (s, 2H), 4.10 (s, 2H), 3.92 (s, 2H), 1.59 (s, 6H), 1.49 (s, 6H); **^{13}C NMR** (126 MHz, Methanol-*d*$_4$) δ 158.02, 136.94, 136.92, 131.92, 131.89, 131.77, 131.49, 131.21, 130.42, 129.70, 57.95, 54.20, 50.82, 43.06, 20.51, 18.66; **IR** = 3421, 3411, 1627, 1592, 1457, 1135, 697 cm^{-1}; **HRMS** Exact Mass ($C_{21}H_{29}N_3$ + H): calculated = 324.2434, measured = 324.2432.

Procedure for the preparation of 1-benzyl-4-(diisopropylamino)-1,5-dihydro-2*H*-imidazol-2-one (3g):

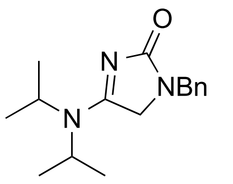

3g (50 mg, 0.17 mmol) was dissolved in 1.7 mL of a 0.5 M sodium iodide solution in acetonitrile. To this, benzyl amine (92 uL, 0.85 mmol) was added and the reaction was allowed to stir for 2 hours. The solution was removed by rotary evaporation, and the crude mixture placed onto a silica gel column. The product was purified using an automated purification system (Teledyne Isco, Inc.) on a normal phase column using a 0-100% gradient of ethyl acetate in hexanes to provide 42 mg of **5g** in 92% yield. **^{1}H NMR** (400 MHz, Chloroform-*d*) δ 7.38-7.22 (m, 5H), 4.57 (s, 2H), 3.85 (s, 2H), 3.75 (sept, *J* = 6.6 Hz, 1H), 3.46 (h, 1H), 1.49 (d, *J* = 6.9 Hz, 6H), 1.20 (d, *J* = 6.7 Hz, 6H); **^{13}C NMR** (125 MHz Chloroform-*d*) δ 172.40, 169.38, 137.55, 128.69, 128.16, 127.48, 50.93, 50.66, 47.79, 46.55, 20.40, 19.66; **IR** = 1691, 1570, 1440, 1359 1132, 1110, 778, 704 cm^{-1}; **HRMS** Exact Mass ($C_{16}H_{23}N_3O$ + H): calculated = 274.1914, measured = 274.1923.

Procedure for the preparation of (*E*)-2-(benzylamino)-*N*'-(dibenzylcarbamoyl)-*N*,*N*-diisopropylacetimidamide (5h):

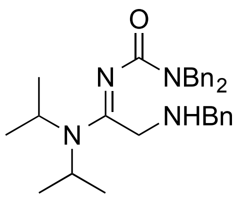

3h (87 mg, 0.22 mmol) was dissolved in 2.2 mL of a 0.5 M sodium iodide solution in acetonitrile. To this, benzyl amine (119 uL, 1.1 mmol) was added and the reaction was allowed to stir for 2 hours. The solution was removed by rotary evaporation, and the crude mixture placed onto a silica gel column. The product was purified using an automated purification system (Teledyne Isco, Inc.) on a normal phase column using a 0-50% gradient of ethyl acetate in hexanes to provide 99 mg of **5h** in 97% yield. **^{1}H NMR** (501 MHz, Chloroform-*d*) δ 7.39-7.18 (m, 14H), 4.56 (s, 2H), 4.50 (s, 2H), 3.82 (s, 1H), 3.81 (s, 1H), 3.50 (s, 1H), 3.38 (s, 2H), 1.36-1.03 (m, 12H); **^{13}C NMR** (126 MHz, Chloroform-*d*) δ 172.42, 169.41, 164.20, 139.78, 137.57, 128.72, 128.55, 128.42, 128.32, 128.25, 128.18, 128.15, 127.51, 127.07, 54.08, 53.00, 50.96, 50.67, 47.83, 46.59, 20.42, 19.68; **IR** = 1698 1573 1442, 1360 1245, 1198, 1112, 745, 700 cm^{-1}; **HRMS** Exact Mass ($C_{30}H_{38}N_4O$ + H): calculated = 471.3119, measured = 471.3132.

Procedure for the preparation of (*E*)-2-(cyclohexylamino)-*N*,*N*-diisopropyl-*N*'-tosylacetimidamide (9a):

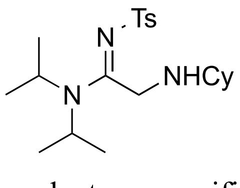

3a (50 mg, 0.15 mmol) was dissolved in 1.5 mL of 0.5 M sodium iodide in acetonitrile and cyclohexylamine (78 uL, 0.76 mmol) was then added and the reaction was allowed to stir at room temperature for 2 hours. The solvent was removed by vacuum and the reaction was redissolved in ~1 mL of dichloromethane and loaded onto a silica gel loading column. The product was purified using an automated purification system (Teledyne Isco, Inc.) on a normal phase column using a 0-50% gradient of ethyl acetate in hexanes to provide 59 mg of **9a** in 98% yield. **^{1}H NMR** (500 MHz, Chloroform-*d*) δ 7.80 (d, 2H), 7.24 (d, *J* = 8.0 Hz, 2H), 4.38 (sept, *J* = 6.6 Hz, 1H), 3.81 (s, 2H), 3.60-3.44 (m, 1H), 2.48-2.41 (m, 1H), 2.39 (s, 3H), 1.89-1.81 (m, 2H), 1.76-1.66 (m, 2H), 1.62-1.55 (m, 1H), 1.35-1.03 (m, 18H); **^{13}C NMR** (126 MHz, Chloroform-*d*) δ 164.30, 141.61, 141.50, 129.01, 125.97, 57.24, 50.09, 48.02, 47.84, 33.02, 26.06, 24.75, 21.40, 20.42, 19.74; **IR =** 2927, 1544, 1447, 1371, 1270, 1135, 1083, 811, 548 cm^{-1}; **HRMS** Exact Mass ($C_{21}H_{35}N_3O_2S$ + H): calculated = 394.2523, measured = 394.2549.

Procedure for the preparation of (*E*)-*N*,*N*-diisopropyl-2-(phenylamino)-*N*'-tosylacetimidamide (9b):

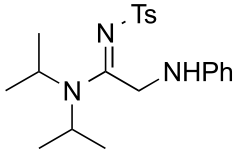

9b was synthesized and purified analogously to **9a** with the following changes. The reaction was heated to 80 °C in a microwave for 8 hours to provide 53 mg of **9b** in 90% yield. **^{1}H NMR** (400 MHz, Chloroform-*d*) δ 7.86-7.78 (m, 2H), 7.29-7.16 (m, 4H), 6.86-6.77 (m, 1H), 6.72-6.64 (m, 2H), 4.37 (s, 2H), 4.15 (m, 1H), 3.61 (s, 1H), 2.40 (s, 3H), 1.37 (d, *J* = 6.5 Hz, 6H), 1.20 (d, *J* = 6.6 Hz, 6H); **^{13}C NMR** (126 MHz, $CDCl_3$) δ 162.69, 147.10, 141.99, 141.34, 129.44, 129.29, 126.17, 119.31, 114.24, 50.70, 48.39, 45.25, 21.58, 20.69, 19.88; **IR** = 1603, 1548, 1372 1269, 1137, 1083, 1051, 548 cm^{-1}; **HRMS** Exact Mass ($C_{21}H_{30}N_3O_2S$ + H): calculated = 388.2054, measured = 388.2044.

Procedure for the preparation of (*E*)-2-(diethylamino)-*N*,*N*-diisopropyl-*N*'-tosylacetimidamide (9c):

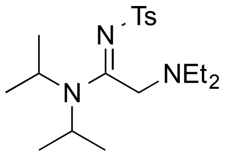

9c was synthesized and purified analogously to **9a** to provide 51 mg of **9c** in 92% yield**. ^{1}H NMR** (500 MHz, Chloroform-*d*) δ 7.79 (d, *J* = 8.1 Hz, 2H), 7.22 (d, *J* = 7.9 Hz, 2H), 4.64 (sept, *J* = 6.7 Hz, 1H), 3.83 (s, 2H), 3.46 (sept, *J* = 6.8 Hz, 1H), 2.58 (q, *J* = 7.1 Hz, 4H), 2.38 (s, 3H), 1.29 (d, *J* = 6.9 Hz, 3H), 1.17 (d, *J* = 6.6 Hz, 6H), 1.03 (t, *J* = 7.1 Hz, 6H); **^{13}C NMR** (126 MHz, Chloroform-*d*) δ 163.68, 141.95, 141.29, 128.89, 125.96, 55.20, 50.43, 47.83, 46.82, 21.37, 20.29, 19.65, 19.14, 11.97; **IR** = 1546, 1369, 1267. 1132, 1080, 1050, 811, 728, 548 cm^{-1}; **HRMS** Exact Mass ($C_{19}H_{33}N_3O_2S$ + H): calculated = 368.2368, measured = 368.2379.

Procedure for the preparation of (*E*)-*N*,*N*-diisopropyl-2-(pyrrolidin-1-yl)-*N*'-tosylacetimidamide (9d):

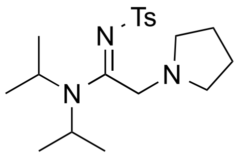

9d was synthesized and purified analogously to **9a** to provide 51 mg of **9d** a 92% yield. **^{1}H NMR** (500 MHz, Chloroform-*d*) δ 7.79 (d, *J* = 7.9 Hz, 2H), 7.22 (d, *J* = 7.9 Hz, 2H), 4.52 (hept, *J* = 6.6 Hz, 1H), 3.91 (s, 2H), 3.47 (sept, *J* = 6.8 Hz, 1H), 2.67-2.58 (m, 4H), 2.37 (s, 3H), 1.72 (q, *J* = 3.2 Hz, 4H), 1.29 (d, *J* = 6.8 Hz, 6H), 1.16 (d, *J* = 6.6 Hz, 6H); **^{13}C NMR** (126 MHz, Chloroform-*d*) δ 162.94, 141.97, 141.26, 128.88, 125.95, 55.26, 52.73, 50.91, 47.84, 23.73, 21.36, 20.37, 19.68; **IR** = 1545, 1372, 1273, 1138, 1985, 1051, 810, 666, 549 cm^{-1}; **HRMS** Exact Mass ($C_{19}H_{31}N_3O_2S$ + H): calculated = 366.2210, measured = 366.2236.

Procedure for the preparation of (*E*)-2-(benzhydrylamino)-*N*,*N*-diisopropyl-*N*'-tosylacetimidamide (9e):

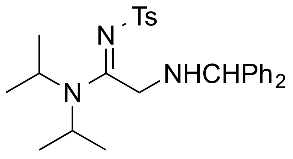

9e was synthesized and purified analogously to **9a** to provide 65 mg of **9e** in 72% yield. **^{1}H NMR** (400 MHz, Chloroform-*d*) δ 7.83-7.75 (m, 2H), 7.45-7.36 (m, 4H), 7.36-7.18 (m, 8H), 4.83 (s, 1H), 4.04 (sept, *J* = 6.7 Hz, 1H), 3.68 (s, 2H), 3.56-3.44 (m, 1H), 2.63 (s, 1H), 2.34 (s, 3H), 1.33 (d, *J* = 6.8 Hz, 6H), 1.12 (d, *J* = 6.6 Hz, 6H); **^{13}C NMR** (101 MHz,

$CDCl_3$) δ 163.44, 142.67, 141.61, 141.37, 128.99, 128.39, 128.37, 128.26, 127.50, 127.45, 127.31, 125.94, 67.95, 49.97, 49.03, 47.90, 21.34, 20.20, 19.66; **IR** = 1547, 1493, 1270, 1138, 1084, 705, 548 cm^{-1}; **HRMS** Exact Mass ($C_{28}H_{35}N_3O_2S$ + H): calculated = 478.2523, measured = 478.2523.

Procedure for the preparation of (*E*)-*N*-benzyl-2-chloro-*N*-((*E*)-2 (diisopropylamino) -2-((methylsulfonyl)imino)ethyl)-*N*'-(methylsulfonyl)acetimidamide (10):

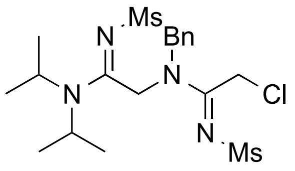

5b (425 mg 1.3 mmol) was dissolved in 13 mL of DCM containing N-methylmorpholine (428 uL, 3.9 mmol) followed by the addition of **2b** (370 mg, 1.95 mmol). The reaction was allowed to stir for 3 hours, then the solvent was removed by rotary evaporation. The crude material was loaded onto silica gel with ~1 mL of dichloromethane and was purified using an automated purification system (Teledyne Isco, Inc.) on a normal phase column using a 0-40% gradient of ethyl acetate in dichloromethane to provide 592 mg of **10** in 95% yield. **^{1}H NMR** (400 MHz, Chloroform-*d*) δ 7.44-7.24 (m, 3H), 7.23-7.15 (m, 2H), 4.95 (s, 2H), 4.77 (s, 2H), 4.60 (s, 2H), 4.02-3.90 (m, 1H), 3.64-3.59 (m, 1H), 3.07-2.97 (m, 3H), 2.94 (s, 3H), 1.40 (s, 6H), 1.23-1.10 (m, 6H); **^{13}C NMR** (101 MHz, Chloroform- *d*) δ 162.87, 159.16, 134.87, 129.13, 128.96, 128.43, 128.21, 126.45, 53.61, 51.69, 50.70, 48.46, 43.76, 43.35, 43.12, 34.40, 20.65, 20.40, 19.88, 19.76; **IR** = 1550, 1451, 1359, 1110, 956, 849, 779, 731, 698, 558, 516 cm^{-1}; **HRMS** Exact Mass ($C_{19}H_{31}ClN_4O_4S_2$ + H): calculated = 479.1548, measured = 479.1550.

Procedure for the preparation of (*E*)-*N*-benzyl-2-(benzylamino)-*N*-((*E*)-2-(diisopropylamino)-2-((methylsulfonyl)imino)ethyl)-*N*'-(methylsulfonyl)acetimidamide (11):

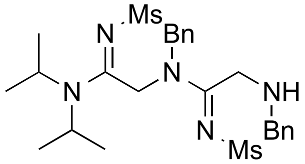

10 (400 mg, 0.835 mmol) was added to a 0.5 M solution of sodium iodide in acetonitrile (8.4 mL, 4.2 mmol NaI) followed by the addition of benzyl amine (460 uL, 4.175 mmol). The reaction was allowed to stir for 3 hours and the solvent was removed under vacuum. The crude material was loaded onto silica gel with dichloromethane and was purified using an automated purification system (Teledyne Isco, Inc.) on a normal phase column using a 0-5% gradient of methanol in dichloromethane to provide 457 mg of **11** in 99% yield. **^{1}H NMR** (500 MHz, Chloroform-*d*) δ 7.39-7.21 (m, 8H), 7.03 (d, *J* = 7.3 Hz, 2H), 4.86-4.71 (m, 4H), 4.08 -4.01 (m, 1H), 3.77 (s, 2H), 3.73 (s, 2H), 3.64 (s, 1H), 3.05 (s, 3H), 2.96 (s, 3H), 1.49-1.37 (m, 6H), 1.26 – 1.17 (m, 6H); **^{13}C NMR** (126 MHz, Chloroform-*d*) δ 167.59, 159.85, 139.03, 135.60, 128.76, 128.41, 128.29, 127.69, 127.18, 126.54, 54.19, 51.64, 50.63, 48.54, 48.36, 47.20, 43.76, 43.19, 43.04, 29.57, 20.52, 19.90; **IR** = 1554, 1272, 1115, 964, 819, 781, 734, 700, 518 cm^{-1}; **HRMS** Exact Mass ($C_{26}H_{39}N_5O_4S_2$ + H): calculated = 550.2516, measured = 550.2532.

Procedure for the preparation of (*E*)-*N*-benzyl-*N*-((*E*)-2-(benzyl((*E*)-2-(diisopropylamino)-2-((methylsulfonyl)imino)ethyl)amino)-2-((methylsulfonyl)imino)ethyl)-2-chloro-*N*'-(methylsulfonyl)acetimidamide (12):

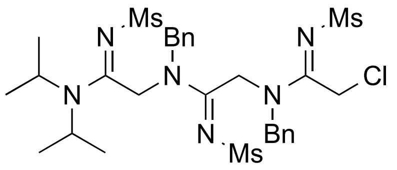

11 (250 mg, 0.455 mmol) was dissolved in 4.5 mL of DCM containing N-methylmorpholine (150 uL, 1.36 mmol) followed by the addition of **2b** (130 mg, 0.682 mmol). The reaction was allowed to stir for 3 hours, then the solvent was removed by rotary evaporation. The crude material was loaded onto silica gel with dichloromethane and was purified using an automated purification system (Teledyne Isco, Inc.) on a normal phase column using a 0-10% gradient of methanol in dichloromethane to provide 280 mg of **12** in 90% yield. ^{1}H **NMR** (500 MHz, Chloroform-*d*) δ 7.40-7.12 (m, 10H), 5.12-4.77 (m, 6H), 4.76-4.46 (m, 4H), 3.86-3.70 (m, 1H), 3.57-3.43 (m, 1H), 3.13-2.80 (m, 9H), 1.30-1.22 (m, 6H), 1.04-0.93 (m, 6H); **^{13}C NMR** (126 MHz, Chloroform-*d*) δ 164.14, 162.99, 162.74, 159.13, 135.27, 135.04, 134.73, 129.57, 129.13, 128.67, 128.47, 128.22, 127.98, 127.61, 126.74, 126.36, 52.95, 52.57, 49.07, 48.60, 48.20, 43.90, 43.85, 43.09, 43.03, 35.46, 34.61, 20.45, 19.85; **IR** = 1556, 1276, 1118, 966, 780, 563, 519 cm^{-1}; **HRMS** Exact Mass ($C_{29}H_{43}ClN_6O_6S_3$ + H): calculated = 703.2167, measured = 703.2155.

Procedure for the preparation of (*E*)-2-(benzyl((*E*)-1,4-dibenzyl-5-((methylsulfonyl)imino)-1,4,5,6-tetrahydropyrazin-2-yl)amino)-*N*,*N*-diisopropyl-*N*'-(methylsulfonyl)acetimidamide (13):

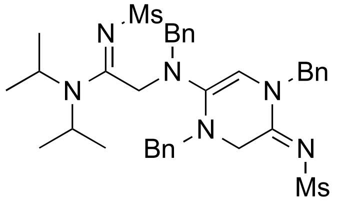

12 (30 mg, 0.043 mmol) was added to was added to a 0.5 M solution of sodium iodide in acetonitrile (0.860 mL, 0.415 mmol NaI) followed by the addition of benzylamine (26.8 uL, 0.215 mmol). The reaction was heated slightly with a heat gun to allow the material to dissolve and was allowed to stir. After 1 hour TLC showed consumption of starting material and the solvent was removed under vacuum. The crude material was loaded onto silica gel with dichloromethane and was purified using an automated organic purification system (Teledyne Isco, Inc.) on a normal phase column using a 0-30% gradient of ethyl acetate in dichloromethane to provide 23 mg of **12** in 79% yield. The compound was immediately characterized, as it is prone to decomposition. **^{1}H NMR** (500 MHz, Acetonitrile-*d*$_3$) δ 7.42-7.22 (m, 9H), 7.22-7.08 (m, 5H), 7.02-6.97 (m, 2H), 5.55 (s, 1H), 4.56 (s, 2H), 4.52 (s, 2H), 4.30 (s, 2H), 4.09-3.97 (m, 5H), 3.66 (hept, *J* = 6.6 Hz, 1H), 2.92 (s, 3H), 2.46 (s, 3H), 1.42 (d, *J* = 6.7 Hz, 6H), 1.07 (d, *J* = 6.6 Hz, 6H); **^{13}C NMR** (126 MHz, Acetonitrile-*d*$_3$) δ 162.47, 155.69, 142.73, 139.16, 139.14, 137.45, 130.15, 129.92, 129.87, 129.82, 129.80, 129.76, 129.65, 129.59, 129.07, 128.90, 128.77, 128.66, 118.69, 118.46, 101.77, 55.71, 54.21, 53.79, 53.28, 52.30, 51.77, 49.18, 48.32, 44.84, 43.94, 43.44, 21.31, 20.67; **IR** = 1548, 1453, 1265, 1109, 964, 817, 734, 700, 517 cm^{-1}; **HRMS** Exact Mass ($C_{35}H_{46}N_6O_4S_2$ + H): calculated = 679.3114, measured = 679.3095.

Procedure for the preparation of (*E*)-*N*-benzyl-*N*-((*E*)-2-(benzyl((*E*)-2-(diisopropylamino)-2-((methylsulfonyl)imino)ethyl)amino)-2-((methylsulfonyl)imino)ethyl)-*N*'-(methylsulfonyl)-2-(((*S*)-1-phenylethyl)amino)acetimidamide (14):

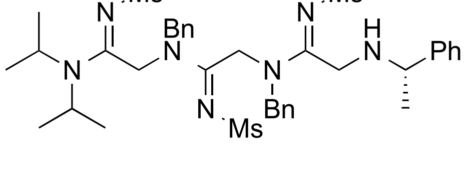

12 (69.6 mg, 0.1 mmol) was dissolved in acetonitrile followed by the addition of (S)-Methylbenzylamine (62 uL, 0.5 mmol). The reaction was heated to 80° C for 1 h in the microwave. The crude material was loaded onto silica gel with dichloromethane and was purified using an

automated purification system (Teledyne Isco, Inc.) on a normal phase column using a 0-10% gradient of methanol in dichloromethane to provide 78 mg of **14** in 99% yield. **^{1}H NMR** (400 MHz, Chloroform-*d*) δ 7.56-6.67 (m, 15H), 5.13-4.56 (m, 8H), 4.0-3.57 (m, 4H), 3.53-3.17 (m, 1H), 3.10-2.71 (m, 10H), 1.74-0.70 (m, 15H). **^{13}C NMR** (101 MHz Chloroform-*d*) δ 163.58, 159.46, 159.20, 144.13, 143.66, 143.12, 135.48, 129.05, 128.98, 128.91, 128.87, 128.76, 128.64, 128.43, 128.23, 127.93, 127.81, 127.74, 127.30, 126.92, 126.79, 126.54, 125.90, 109.98, 59.10, 56.10, 53.76, 52.79, 51.21, 50.77, 48.65, 47.86, 46.51, 43.97, 43.83, 43.10, 42.41, 29.68, 23.76, 20.55, 19.87, 14.10; **IR** = 1550, 1452, 1274, 1114, 965, 855, 778, 733, 700, 558, 517 cm^{-1}; **HRMS** Mass ($C_{37}H_{53}N_7O_6S_3$ + H): calculated = 788.3292, measured = 788.3275; $[\alpha]_D^{25}$ = -9.0° (c 0.01, CH_2Cl_2).

Procedure for the preparation of (*E*)-*N*-benzyl-*N*-((*E*)-2-(benzyl((*E*)-2-(diisopropylamino)-2-((methylsulfonyl)imino)ethyl)amino)-2-((methylsulfonyl)imino)ethyl)-2-(dibenzylamino)-*N*'-(methylsulfonyl)acetimidamide (15):

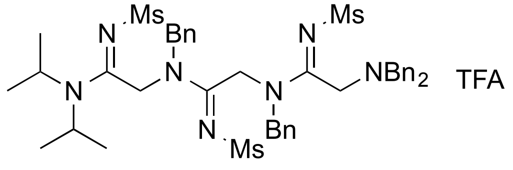

14

12 (50 mg 0.071 mmol) was dissolved in acetonitrile and dibenzylamine was added (68 uL, 0.355 mmol), the reaction was then placed in a microwave at 80° C for 16 hours. The crude reaction was directly loaded onto a C18 pre-packed loading column and used with an automated purification system (Teledyne Isco, Inc.) on a C18 reverse phase column using a 0-100% gradient of acetonitrile in water with a 0.1% TFA buffer. The fractions were combined and the solvent was then doubly azeotroped with anhydrous ethanol and dried on high vacuum to provide 69.5 mg of **15** in 99% yield assuming a single TFA salt. **^{1}H NMR** (400 MHz, Chloroform-*d*) δ 7.62-6.69 (m, 19H), 5.31-4.94 (m, 3H), 4.92-4.53 (m, 4H), 4.44 (s, 1H), 4.20 (s, 1H), 3.99 (s, 1H), 3.82 (s, 3H), 3.69-3.38 (m, 2H), 3.21-2.60 (m, 9H), 1.54-0.71 (m, 12H); **^{13}C NMR** (101 MHz, Chloroform-*d*) δ 162.85, 161.21, 160.44, 160.07, 158.74, 135.02, 134.60, 130.28, 130.01, 129.73, 129.63, 129.41, 129.21, 128.99, 128.76, 128.51, 128.24, 128.10, 127.91, 126.28, 125.78, 117.00, 114.14, 109.95, 58.76, 58.10, 54.77, 52.92, 52.53, 52.48, 51.49, 49.27, 48.56, 44.16, 43.86, 43.62, 43.18, 42.85, 20.30, 19.70; **IR** 1556, 1279 1189, 968 728, 700, 562, 519 cm^{-1}; **HRMS** Exact Mass ($C_{43}H_{57}N_7O_6S_3$ + H): calculated = 864.3605, measured = 864.3620

Procedure for the preparation of (*E*)-*N*-benzyl-*N*-((*E*)-2-(benzyl((*E*)-2-(diisopropylamino)-2-((methylsulfonyl)imino)ethyl)amino)-2-((methylsulfonyl)imino)ethyl)-2-((*E*)-2-chloro-*N*'-(methylsulfonyl)-*N*-((*S*)-1-phenylethyl)acetimidamido)-*N*'-(methylsulfonyl)acetimidamide (16):

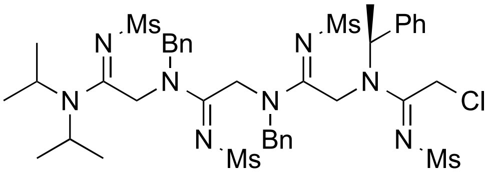

13 (15.5 mg, 0.0197 mmol) was dissolved in 400 uL of acetonitrile and N-Methylmorpholine (6.5 uL, 0.0591 uL) was added followed by the addition of **3b** (5.6 mg, 0.0296 mmol). The reaction was heated to 60° C in the microwave for 90 minutes resulting in a dark black solution. The crude reaction was poured into a seperatory funnel containing 10 mL of DCM and was washed with saturated $NaHCO_3$ (10 ml), saturated NH_4Cl (10 mL), and brine (10 mL). The organic layer was then dried over sodium sulfate and removed by rotary evaporation.

The crude material was loaded onto silica gel with dichloromethane and was purified using an automated purification system (Teledyne Isco, Inc.) on a normal phase column using a 0-100% gradient ethyl acetate in hexane to provide 15.3 mg of **16** in 83% yield. ^{1}**H NMR** (800 MHz, Chloroform-*d*) δ 7.53-6.82 (m, 13H), 6.82-6.42 (m, 2H), 5.79 (s, 2H), 5.61-5.01 (m, 2H), 5.02-3.96 (m, 6H), 3.96-3.31 (m, 3H), 3.29-2.61 (m, 12H), 1.77 (d, *J* = 6.9 Hz, 2H), 1.52-0.76 (m, 15H); ^{13}**C NMR** (200 MHz, Chloroform-*d*) δ 165.45, 163.06, 162.79, 159.03, 138.69, 134.96, 134.58, 129.19, 129.10, 128.57, 128.26, 127.88, 127.53, 127.12, 126.99, 126.78, 126.12, 125.40, 57.33, 54.22, 53.68, 52.08, 51.00, 50.04, 48.67, 47.53, 45.74, 44.39, 44.25, 44.06, 44.02, 43.86, 43.61, 43.26, 43.21, 36.50, 35.43, 29.68, 29.34, 20.66, 20.45, 20.05, 19.70, 19.51, 14.35; **IR** = 1558, 1444, 1275, 1116, 965, 780, 733, 699, 524 cm^{-1}; **HRMS** Exact Mass ($C_{40}H_{57}ClN_8O_8S_4$ + H): calculated = 941.2943, measured = 941.2954; $[\alpha]_D^{25}$ = -50.0° (c 0.01, CH_2Cl_2).

Procedure for the preparation of 2-chloro-*N*,*N*-diethyl-*N*'-phenethylacetimidamide (37):

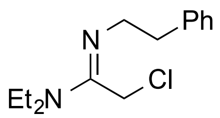

8i (197 mg, 1 mmol) was dissolved in toluene (4 mL) and PCl_5 (228 mg, 1.1 mmol) was added. The reaction was allowed to stir at room temperature overnight (14h). The reaction was then cooled in an ice bath to 0° C followed by the drop-wise addition of diethylamine (1.05 mL, 10 mmol). The reaction was kept at 0° C for 2 h and then the solvent was removed by rotary evaporation and the residual material was directly loaded onto a silica gel column and purified using an automated purification system (Teledyne Isco, Inc.) on a normal phase column using a from 0-15% methanol in dichloromethane resulting in a very broad peak that, after removal of solvent, provide 284 mg of a mixture containing **35.** NMR integration showed a mixture of 0.68 equivalents of diethylamine, and 1.0 equivalent of **35** as an HCl salt and 0.29 equivalents of DCM. Analysis of the integration reveals this mixture contains 197 mg of **37** a 78% NMR-yield. This mixture was then characterized $^{1\cdot}$**H NMR** (400 MHz, Chloroform-*d*) δ 10.85 (s, 1H), 9.68 (s 0.4H), 7.34-7.16 (m, 5H), 3.92 (q, *J* = 7.1 Hz, 2H), 3.78 (q, *J* = 6.5 Hz, 2H), 3.56 (s, 2H), 3.41 (q, *J* = 7.2 Hz, 2H), 3.15 (t, *J* = 6.7 Hz, 2H), 1.30 (t, *J* = 7.1 Hz, 3H), 1.23 (t, 3H); ^{13}**C NMR** (126 MHz, $CDCl_3$) δ 159.02, 137.89, 129.46, 129.10, 127.24, 46.88, 46.52, 45.86, 42.42, 36.33, 32.80, 14.31, 11.60, 11.37; **IR** = 1636, 1497, 1454, 1387, 1355, 1193, 1079, 752, 704 cm^{-1}; **HRMS** Exact Mass (C14H21ClN2 + H^+): calculated = 253.1466, measured = 253.1412.

6. Solid-Phase Procedures:

Reagents:
All solvent and reagents were used as purchased. DMF/DCM were used from anhydrous bottles. Amines were used as purchased. Rink Amide Resin was purchased from Peptides international (0.61 or 0.51 mmol/g loading). All reactions were carried out in either glass or plastic tubes and were rotated under normal atmosphere. NMP and NMM were purchased as >99% purity and were stored in a dry-box over $CaSO_4$ after opening.

Washing:
Upon completion, reactions were washed 2x DCM, then alternating (DCM/DMF) 3x then DCM 2x using ~2-3 mL of solvent per 100 mg of resin. After this, the resin was then blown with dry nitrogen and placed under high vacuum until dry.

Cyclohexyl/Tetrahydropyranyl Resin:
This resin was prepared on large scale (4 g) using previously published methods[4] and was stored in the -20 °C freezer. Yields for all solid phase syntheses were calculated assuming full conversion of the Resin-NH-Fmoc to the Resin-NH-Cyclohexyl. Reductive amination with Tetrahydro-4H-pyran-4-one was carried out under analogous procedure to that of cyclohexanone. Resin loading levels were adjusted (0.61 mmol to 0.63 mmol/g and 0.51 mmol/g to 0.52 mmol/g) due to the change in mass of the resin functionality. The equation used to calculate such changes is found in the supplementary information of this reference[5].

Amidintion:
To 200 mg of resin (0.12 mmol) was added 3 mL of NMP, 200 uL of NMM (~2.15 mmol, 17.6 equiv), followed 800 uL of 1 M imidoyl chloride stock solution in DCM (0.8 mmol, 6.5 equiv) or in the case of the imidoyl chloride **2b** 152 mg of solid (0.8 mmol, 6.5 equiv). The reaction was immediately agitated to ensure even mixing and was placed on a rotator for 12-14 hours under ambient atmosphere. It was observed that the reaction mixture turns dark black and warms over the course of several minutes after the addition of the imidoyl chloride.

Amination:
To 200 mg of resin 4 mL of 1 M amine in dry DMF was added. The reaction was immediately agitated and allowed to rotate for 12 hours under ambient atmosphere.

Cleavage:
Unless stated otherwise, all peptides were cleaved with 3 mL/100 mg of resin of a 1:19 TFA:DCM solution unless otherwise noted for 1 hour in a glass tube (to prevent leaching of plastic contaminants). The resin was then washed with an extra 3 ml/100 mg resin of cleavage cocktail and then methanol. 10 uL of this crude mixture was dissolved in 500 uL of CH_3CN and injected for crude LCMS analysis of cleavage products.

Purification:

All crude reaction cleavage solutions were removed by rotary evaporation. Normal or reverse phase chromatography was used for purification. Reverse phase was carried out with Water/Acetonitrile buffered with either 0.1% TFA or FA see individual compound preparation.

Procedure for the preparation of (*E*)-2-(benzylamino)-*N*-cyclohexyl-*N*'-tosylacetimidamide (22):

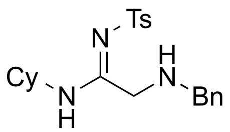

200 mg of cyclohexyl resin with a loading of 0.62 mmol/g was treated under the standard solid phase synthesis procedure. The crude cleavage solution was poured into a separatory funnel containing 10 mL of DCM and was washed with 1 M NaOH (10 mL). The organic layer was dried over sodium sulfate and the crude material was loaded onto silica gel and was purified using an automated purification system (Teledyne Isco, Inc.) on a normal phase column using a 0-60% gradient of ethyl acetate in hexanes to provide 34.7 mg of **22** in 70% yield. **^{1}H NMR** (400 MHz, Chloroform-*d*) δ 7.84 (s, 1H), 7.76 (d, *J* = 7.9 Hz, 2H), 7.34-7.14 (m, 7H), 3.95 (s 2H), 3.86-3.72 (m, 1H), 3.68 (s, 2H), 2.37 (s, 3H), 1.86-1.49 (m, 5H), 1.40-1.05 (m, 5H); **^{13}C NMR** (101 MHz, Chloroform-*d*) δ 163.48, 163.13, 156.85, 143.14, 138.94, 130.10, 130.02, 129.99, 129.84, 129.60, 129.49, 129.44, 129.33, 126.42, 126.18, 52.93, 51.50, 48.19, 30.91, 25.12, 24.27, 21.51; **IR** = 1564, 1452, 1274, 1141, 1085, 814, 748, 698, 669, 616, 555 cm^{-1}; **HRMS** Exact Mass ($C_{22}H_{29}N_3O_2S$ + H): calculated = 400.2053, measured = 400.2042.

Procedure for the preparation of (*E*)-*N*-benzyl-*N*-((*E*)-2-(cyclohexylamino)-2-(tosylimino)ethyl)-2-(dibenzylamino)-*N*'-tosylacetimidamide (23):

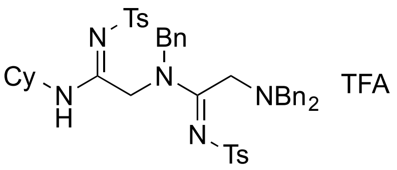

200 mg of cyclohexyl resin with a loading of 0.62 mmol/g was treated under the standard solid phase synthesis procedure except for the last amination procedure. The final amination procedure used 1M dibenzylamine in DMF containing 0.5 M KI. The resin was then cleaved using the standard procedure and the crude cleavage solution was removed by rotary evaporation. The crude material was redissolved in several milliliters of 1:1:0.01 CH-$_3$CN:H_2O:TFA and purified using an automated purification system (Teledyne Isco, Inc.) on a C18 reverse phase column using a gradient of 30-100% acetonitrile in water buffered with 0.1% TFA. The fractions were combined and the crude material was doubly azeotroped with anhydrous ethanol and dried on high vacuum to provide 67 mg of **23** in 60% yield assuming a single TFA salt. **^{1}H NMR** (400 MHz, Chloroform-*d*) δ 8.35-6.21 (m, 23H), 5.21-2.85 (m, 10H), 2.52-2.20 (m, 6H), 2.17-0.40 (m, 10H); **^{13}C NMR** (101 MHz Chloroform-*d*) δ 166.33, 164.06, 160.44, 142.85, 142.69, 142.00, 140.56, 140.04, 139.91, 137.46, 135.19, 134.66, 129.52, 129.48, 129.36, 129.34, 129.31, 129.24, 129.18, 129.14, 129.07, 129.03, 128.96, 128.64, 128.60, 128.50, 128.34, 128.21, 127.76, 127.50, 126.67, 126.40, 126.10, 126.07, 59.35, 59.17, 58.55, 54.88, 54.13, 53.26, 52.69, 51.84, 50.50, 48.01, 33.15, 32.87, 31.23, 30.93, 29.73, 29.70, 25.25, 25.05, 24.75, 24.43, 24.03, 23.83, 22.68, 21.53, 21.50, 21.46, 21.42; **IR** 1545, 1452, 1278, 1144, 1087, 814, 750, 696, 578 cm^{-1}; **HRMS** Exact Mass ($C_{45}H_{51}N_5O_4S_2$ + H): calculated = 790.3455, measured = 790.3459.

Procedure for the preparation of (*E*)-*N*-benzyl-*N*-((*E*)-2-(benzyl((*E*)-2-(cyclohexylamino)-2-(tosylimino)ethyl)amino)-2-(tosylimino)ethyl)-2-chloro-*N*'-tosylacetimidamide (24):

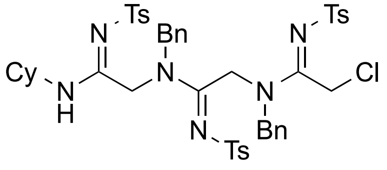

200 mg of cyclohexyl resin with a loading of 0.51 mmol/g was treated under the standard solid phase synthesis procedure. After cleavage the solvent was removed by rotary evaporation and the residual compound was dried under high vacuum. The crude material was loaded onto silica gel and was purified using an automated purification system (Teledyne Isco, Inc.) on a normal phase column using a 0-70% gradient of ethyl acetate in hexanes to provide 43.7 mg of **24** in 45% yield. **^{1}H NMR** (400 MHz, Chloroform-*d*) δ 8.13-7.53 (m, 6H), 7.40-6.84 (m, 14H), 5.45-3.77 (m, 9H), 3.64-3.41 (m, 1H), 3.07-2.85 (m, 1H), 2.47-2.28 (m, 9H), 1.71-0.83 (m, 10H); **^{13}C NMR** (101 MHz, Chloroform-*d*) δ 171.06, 164.60, 163.37, 163.21, 162.60, 162.44, 161.27, 160.35, 160.27, 143.48, 143.03, 142.93, 142.72, 142.52, 142.37, 142.08, 140.11, 139.98, 139.35, 139.11, 135.36, 134.88, 134.11, 134.08, 133.88, 132.92, 129.61, 129.59, 129.44, 129.38, 129.23, 129.13, 129.10, 129.05, 129.03, 128.97, 128.66, 128.38, 128.31, 127.98, 127.88, 126.88, 126.46, 126.14, 126.10, 126.05, 125.82, 60.32, 55.08, 54.59, 54.03, 53.85, 51.97, 50.70, 49.24, 48.75, 48.17, 47.54, 46.36, 45.10, 35.81, 34.58, 34.22, 34.01, 33.76, 33.14, 32.85, 31.86, 31.58, 30.93, 30.30, 29.70, 29.63, 29.59, 29.55, 29.29, 29.25, 29.19, 28.88, 25.03, 24.74, 24.57, 23.90, 23.78, 22.62, 21.56, 21.49, 21.47, 21.43, 21.42, 21.40, 20.99, 14.15, 14.07; **IR** 1555, 1452, 1283, 1144, 1088, 762, 686, 578 cm^{-1}; **HRMS** Exact Mass ($C_{47}H_{53}ClN_6O_6S_3$ + H): calculated = 929.2950, measured = 929.2979.

Procedure for the preparation of (*E*)-*N*-benzyl-*N*-((*E*)-2-(benzyl((*E*)-2-(cyclohexylamino)-2-(tosylimino)ethyl)amino)-2-(tosylimino)ethyl)-2-(dibenzylamino)-*N*'-tosylacetimidamide (25):

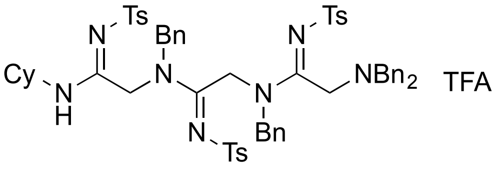

200 mg of cyclohexyl resin with a loading of 0.51 mmol/g was treated under the standard solid phase synthesis procedure. After cleavage the solvent was removed by rotary evaporation and the residual compound was dried under high vacuum. The crude material was loaded onto an automated purification system (Teledyne Isco, Inc.) on a C18 reverse phase column using a 0-100% gradient of acetonitrile in water buffered with 0.1% TFA. The fractions were combined and the crude material was doubly azeotroped with anhydrous ethanol and dried on high vacuum to provide 46.6 mg of **25** in 38% yield assuming a single TFA salt. **^{1}H NMR** (400 MHz, Chloroform-*d*) δ 8.12-6.51 (m, 32H), 5.38-5.03 (m, 2H), 4.7-4.10 (m, 5H), 4.06-3.39 (m, 5H), 3.34-3.23 (m, 1H), 2.58-2.26 (m, 9H), 1.63-0.83 (m, 8H), 0.66-0.49 (m, 2H); **^{13}C NMR** (101 MHz, Chloroform-*d*) δ 166.33, 164.06, 160.44, 142.85, 142.69, 142.00, 140.56, 140.04, 139.91, 137.46, 135.19, 134.66, 129.63, 129.52, 129.48, 129.36, 129.34, 129.31, 129.24, 129.18, 129.14, 129.07, 129.03, 128.97, 128.87, 128.64, 128.60, 128.50, 128.34, 128.21, 127.91, 127.76, 127.50, 126.67, 126.40, 126.29, 126.07, 59.35, 59.17, 58.55, 54.88, 54.13, 53.26, 52.69, 51.84, 50.50, 48.01, 33.15, 32.87, 31.23, 30.93, 29.73, 29.70, 25.25, 25.05, 24.75, 24.43, 24.03, 23.83, 22.68, 21.53, 21.50, 21.46, 21.42; **IR** = 1545, 1452, 1282, 1146, 1088, 697, 578 cm^{-1}; **HRMS** Exact Mass ($C_{61}H_{67}N_7O_6S_3$ + H): calculated = 1090.4388, measured = 1090.4392

Procedure for the preparation of (*E*)-*N*-benzyl-*N*-((*E*)-2-(benzyl((*E*)-2-(cyclohexylamino)-2-(tosylimino)ethyl)amino)-2-(tosylimino)ethyl)-2-((*E*)-2-chloro-*N*-((*S*)-1-phenylethyl)-*N'*-tosylacetimidamido)-*N'*-tosylacetimidamide (26):

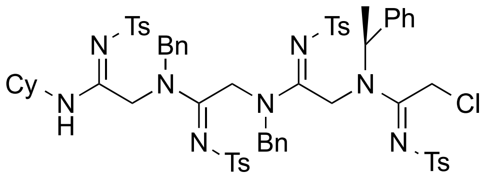

200 mg of cyclohexyl resin with a loading of 0.51 mmol/g was treated under the standard solid phase synthesis procedure. The last amidination coupling was carried out in triplicate each time for 1 hour at 60° C in the microwave achieving ~80% overall conversion to the product by LCMS analysis of the cleavage. The crude cleavage solution was removed by rotary evaporation and the residual compound was then dissolved in 10 mL of DCM and washed with saturated sodium bicarbonate and the residual organic layer was then dried over sodium sulfate. The crude material was loaded onto silica gel and was purified using an automated organic purification system (Teledyne Isco, Inc.) on normal phase column using 0-50% ethyl acetate in hexanes. The fractions were combined and the crude material still contained impurities. The solvent removed by rotary evaporation and the residual material was repurifed by HPLC using a 70-100% gradient of acetonitrile in water buffered with 0.1% TFA. The fractions were combined and lyophilized to give 24.7 mg of **26** in 20% yield. **^{1}H NMR** (800 MHz, Chloroform-*d*) δ 8.27-6.44 (m, 32H), 5.88-2.82 (m, 13H), 2.49-2.23 (m, 2H), 2.18-0.47 (m, 22H); **^{13}C NMR** (201 MHz, Chloroform-*d*) δ163.96, 162.42, 162.10, 161.80, 161.18, 159.87, 158.93, 158.73, 143.36, 143.18, 142.78, 142.60, 142.50, 142.42, 142.15, 142.08, 141.99, 141.93, 140.71, 140.64, 140.45, 140.28, 140.16, 140.12, 139.69, 139.07, 134.55, 133.85, 133.58, 129.81, 129.69, 129.61, 129.48, 129.44, 129.37, 129.23, 129.18, 129.15, 129.12, 129.04, 129.01, 129.00, 128.78, 128.52, 128.01, 127.53, 127.35, 127.07, 126.82, 126.48, 126.43, 126.34, 126.29, 126.25, 126.15, 126.08, 126.03, 125.94, 68.16, 54.54, 54.06, 52.61, 52.25, 50.86, 48.99, 47.83, 47.20, 35.99, 35.46, 34.76, 33.33, 32.99, 31.91, 31.42, 31.26, 30.86, 30.35, 29.68, 29.67, 29.65, 29.63, 29.58, 29.43, 29.34, 29.25, 29.23, 29.07, 29.01, 28.91, 26.70, 25.16, 24.74, 24.72, 24.61, 24.00, 23.74, 22.68, 21.59, 21.53, 21.49, 21.46, 21.45, 21.44, 14.10; **IR** = 1550, 1453, 1283, 1145, 1088, 695, 554 cm^{-1}; **HRMS** Exact Mass ($C_{64}H_{71}ClN_8O_8S_4$ + H): calculated = 1243.4039, measured = 1243.4087; **$[\alpha]_D^{25}$** = -46.0° (c 0.01, CH_2Cl_2).

Procedure for the preparation of *N*-((2*E*,5*E*)-1,4-dibenzyl-5-(cyclohexylimino)piperazin-2-ylidene)-4-methylbenzenesulfonamide (27):

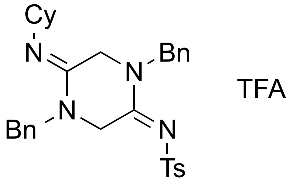

200 mg of cyclohexyl resin with a loading of 0.62 mmol/g was treated under the standard solid phase synthesis procedure. The crude cleavage solution was removed by rotary evaporation and the crude material was loaded onto an automated purification system (Teledyne Isco, Inc.) on a C18 reverse phase column and purified using a 0-100% gradient of acetonitrile in water buffered with 0.1% TFA. The fractions were combined and the material was doubly azeotroped with anhydrous ethanol and dried on high vacuum to provide 31.4 mg of **27** in 48% yield assuming a single TFA salt**.** **^{1}H NMR** (400 MHz, Chloroform-*d*) δ 9.77-9.71 (m, 1H), 7.63 (d, *J* = 8.2 Hz, 2H), 7.42-7.18 (m, 10H), 7.08-7.00 (m, 2H), 5.11 (s, 2H), 5.03 (s, 2H), 4.73 (s, 2H), 4.45 (s, 2H), 2.83-2.78 (m, 1H), 2.41 (s, 4H), 1.67-1.44 (m, 5H), 1.36-1.22 (m, 2H), 1.07-0.88 (m, 3H); **^{13}C NMR** (101 MHz, Chloroform-*d*) δ 159.58, 158.00, 143.27, 138.95, 133.47, 131.99, 129.53, 129.36, 129.29, 129.09, 128.94, 128.54,

128.48, 126.29, 116.63, 113.77, 56.45, 55.08, 51.43, 49.53, 43.93, 32.01, 24.76, 24.20, 21.52; **IR** = 1655, 1576, 1454, 1270, 1142 1086, 982, 893, 813, 732, 690, 553 cm^{-1}; **HRMS** Exact Mass ($C_{31}H_{36}N_4O_2S$ + H): calculated = 529.2632, measured = 529.2684.

Procedure for the preparation of (*E*)-*N*-benzyl-*N*-((*E*)-2-(benzyl((*E*)-2-(cyclohexylamino)-2-(tosylimino)ethyl)amino)-2-((methylsulfonyl)imino)ethyl)-2-(dibenzylamino)-*N*'-(methylsulfonyl)acetimidamide (28):

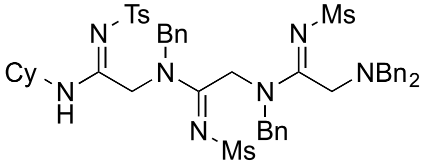

TFA

200 mg of cyclohexyl resin with a loading of 0.62 mmol/g was treated under the standard solid phase synthesis procedure. After cleavage the solvent was removed by rotary evaporation and the residual compound was dried under high vacuum. The crude material was loaded onto an automated purification system (Teledyne Isco, Inc.) on a C18 reverse phase column using a 0-100% gradient of acetonitrile in water buffered with 0.1% TFA. The fractions were combined and the crude material was doubly azeotroped with anhydrous ethanol and dried on high vacuum to provide 59.2 mg of **28** in 43% yield assuming a single TFA salt. **^{1}H NMR** (500 MHz, DMSO-d_6) δ 8.08-6.61 (m, 25H), 5.05-3.43 (m, 14H), 3.07-2.52 (m, 7H), 2.44-2.26 (m, 2H), 1.81-0.75 (m, 10H); **^{13}C NMR** (201 MHz, DMSO-d_6) δ 168.38, 166.82, 164.10, 144.82, 143.91, 140.69, 140.29, 133.16, 132.33, 132.00, 131.68, 131.63, 131.41, 131.33, 131.08, 130.44, 129.86, 128.75, 128.67, 61.59, 56.79, 53.40, 46.70, 46.53, 33.79, 32.07, 27.99, 27.72, 24.03, 23.96; **IR** = 1551, 1438, 1284, 1141, 1032, 515 cm^{-1}; **HRMS** Exact Mass ($C_{49}H_{59}N_7O_6S_3$ + H): calculated = 938.3762, measured = 938.3767

Procedure for the preparation of (*E*)-*N*-benzyl-*N*-((*E*)-2-(benzyl((*E*)-2-(cyclohexylamino)-2-((methylsulfonyl)imino)ethyl)amino)-2-((methylsulfonyl)imino)ethyl)-2-(dibenzylamino)-*N*'-(methylsulfonyl)acetimidamide (29):

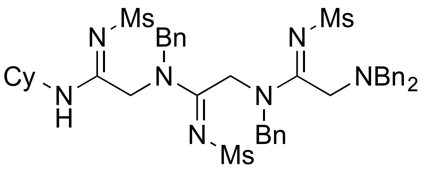

FA

200 mg of cyclohexyl resin with a loading of 0.62 mmol/g was treated under the standard solid phase synthesis procedure. After cleavage, the solution was removed by rotary evaporation and the residual compound was dried under high vacuum. The crude material was loaded onto an automated purification system (Teledyne Isco, Inc.) on a C18 reverse phase column and purified using a 0-100% gradient of acetonitrile in water buffered with 0.1% formic acid. The fractions were combined and the crude material was lyophilized to provide 37 mg of **29** in 37% yield assuming a formic acid salt. **^{1}H NMR** (501 MHz, Chloroform-*d*) δ 7.41-6.76 (m, 20H), 6.07 (s, 1H), 5.18-4.92 (m, 2H), 4.62-4.16 (m, 4H), 3.91 (s, 2H), 3.76-3.48 (m, 5H), 3.15-2.56 (m, 9H), 1.95-0.72 (m, 12H); **^{13}C NMR** (126 MHz, Chloroform-*d*) δ 166.86, 163.69, 160.38, 137.59, 134.99, 134.66, 129.73, 129.46, 129.22, 129.14, 128.94, 128.74, 128.68, 128.63, 128.50, 128.45, 128.39, 128.33, 127.86, 127.55, 127.50, 127.38, 126.72, 125.60, 59.46, 59.31, 54.58, 53.41, 52.89, 52.55, 51.88, 50.73, 48.16, 43.94, 43.78, 43.21, 33.31, 31.34, 29.66, 25.33, 25.14, 24.80, 24.07; **IR** = 1546, 1452, 1279, 1122, 966, 859, 788, 735, 700, 561 cm^{-1}; **HRMS** Exact Mass ($C_{43}H_{55}N_7O_6S_3$ + H): calculated = 862.3449, measured = 862.3420.

Procedure for the preparation of *N*-(2-((*E*)-*N*-((*E*)-2-((2-acetamidoethyl)((*E*)-2-(cyclohexylamino)-2-((methylsulfonyl)imino)ethyl)amino)-2-((methylsulfonyl)imino)ethyl)-*N*'-(methylsulfonyl)-2-(pyrrolidin-1-yl)acetimidamido)ethyl)acetamide (30):

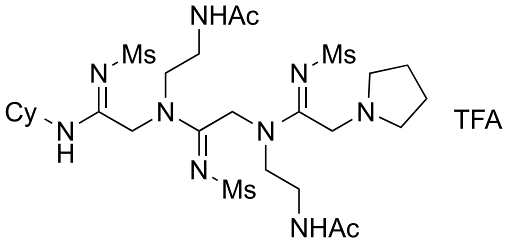

200 mg of cyclohexyl resin with a loading of 0.62 mmol/g was treated under the standard solid phase synthesis procedure. After cleavage, the solvent was removed by rotary evaporation and the residual compound was dried under high vacuum. The crude material was loaded onto an automated organic purification system (Teledyne Isco, Inc.) on a C18 reverse phase column using a 0-100% gradient of acetonitrile in water buffered with 0.1% TFA. The fractions were combined and the crude material was doubly azeotroped with anhydrous ethanol and dried on high vacuum to provide 29.2 mg of **30** in 28% yield assuming a single TFA salt. **^{1}H NMR** (500 MHz, Methanol-d_4) δ 5.17 (s, 1H), 4.78 (s, 2H), 4.67 (s, 2H), 4.03-3.77 (m, 4H), 3.61 (s, 1H), 3.53-3.37 (m, 6H), 3.27-3.23 (m, 1H), 3.14-2.96 (m, 9H), 2.14 (s, 6H), 2.03-1.93 (m, 6H), 1.89-1.82 (m, 3H), 1.81-1.75 (m, 2H), 1.70-1.63 (m, 1H), 1.47-1.27 (m, 7H), 1.23-1.14 (m, 1H); **^{13}C NMR** (126 MHz, Methanol-d_4) δ 174.84, 174.33, 165.00, 162.90, 162.47, 159.82, 57.60, 54.44, 52.61, 51.94, 49.88, 44.46, 43.88, 43.67, 43.62, 39.40, 37.49, 34.18, 32.63, 30.77, 26.49, 26.36, 25.33, 23.64, 23.48, 22.72, 22.58; **IR** = 1671, 1558, 1267, 1201, 1122, 799, 519 = cm^{-1}; **HRMS** Exact Mass ($C_{27}H_{51}N_9O_8S_3$ + H): calculated = 726.3095, measured = 726.3117.

Procedure for the preparation of (*E*)-*N*-benzyl-*N*-((*E*)-2-(benzyl((*E*)-2-(cyclohexylamino)-2-(tosylimino)ethyl)amino)-2-((isopropylsulfonyl)imino)ethyl)-*N*'-((5-chlorothiophen-2-yl)sulfonyl)-2-((2-methoxyethyl)(methyl)amino)acetimidamide (31):

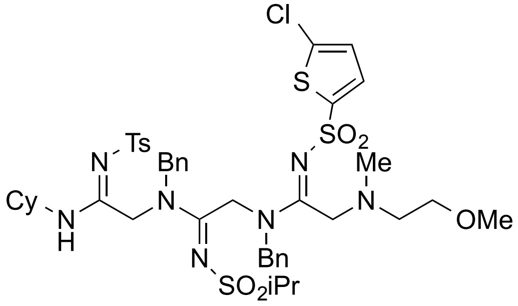

200 mg of cyclohexyl resin with a loading of 0.51 mmol/g was treated under the standard solid phase synthesis procedure. The crude cleavage solution was extracted with 1 M NaOH (10 mL). The aqueous layer was washed two times with DCM and the combined organic layers were dried over sodium sulfate. The crude material was loaded onto an automated purification system (Teledyne Isco, Inc.) on a normal phase column and purified using 0-50% ethyl acetate in hexanes. The fractions were combined and to provide 39 mg of **31** in 40% yield. **^{1}H NMR** (501 MHz, Chloroform-*d*) δ 8.66-8.00 (m, 2H), 7.87-6.45 (m, 16H), 5.38 (s, 2H), 5.22-4.00 (m, 7H), 3.92-3.49 (m, 3H), 3.45-3.14 (m, 5H), 3.08-2.99 (m, 1H), 2.95-2.68 (m, 3H), 2.47-2.26 (m, 3H), 1.86-1.44 (m, 5H), 1.44-0.95 (m, 12H); **^{13}C NMR** (126 MHz, Chloroform-*d*) δ 164.32, 161.42, 161.14, 160.84, 160.52, 160.29, 159.92, 159.04, 142.09, 140.74, 136.96, 134.11, 133.89, 131.35, 130.91, 129.75, 129.63, 129.55, 129.24, 129.13, 129.02, 128.88, 128.81, 128.75, 128.49, 126.88, 126.28, 126.13, 67.82, 65.65, 58.93, 58.83, 57.61, 56.62, 55.79, 55.61, 55.41, 54.86, 54.04, 53.12, 51.78, 49.99, 49.68, 41.13, 31.29, 30.99, 29.63, 25.14, 25.09, 24.72, 21.48, 21.44, 16.54; **IR** = 1536, 1277, 1140, 1117, 1087, 990, 862, 755, 695, 611, cm^{-1}; **HRMS** Exact Mass ($C_{44}H_{58}ClN_7O_7S_4$ + H): calculated = 960.3042, measured = 960.3049.

Procedure for the preparation of (*E*)-*N*-(2-methoxyethyl)-*N*-((*E*)-2-((2-methoxyethyl)((*E*)-2-((tetrahydro-2*H*-pyran-4-yl)amino)-2-(tosylimino)ethyl)amino)-2-((methylsulfonyl)imino)ethyl)-2-(pyrrolidin-1-yl)-*N*'-tosylacetimidamide (32):

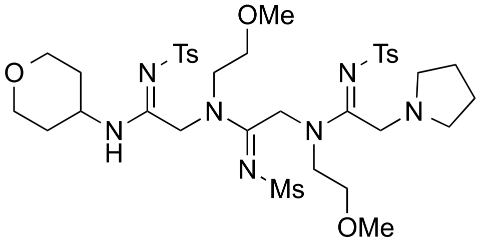

TFA

1 g of tetrahydropyranyl labeled resin with a loading of 0.52 mmol/g was treated under standard the solid phase synthesis procedure. After cleavage the solvent was removed by rotary evaporation and the residual compound was dried under high vacuum. The crude material was loaded onto an automated purification system (Teledyne Isco, Inc.) on a C18 reverse phase column using a 0-100% gradient of acetonitrile in water buffered with 0.1% TFA. The fractions were combined and the crude material was doubly azeotroped with anhydrous ethanol and dried on high vacuum to provide 228 mg of **32** in 47% yield assuming a single TFA salt. ^{1}**H NMR** (500 MHz, Chloroform-*d*) δ 7.77 (d, *J* = 8.0 Hz, 3H), 7.71 (d, *J* = 7.5 Hz, 2H), 7.40 (d, *J* = 7.9 Hz, 2H), 7.27 (d, *J* = 8.8 Hz, 4H), 4.87 (s, 2H), 4.39-4.35 (m, 2H), 4.25 (s, 1H), 4.13-4.06 (m, 2H), 3.96-3.85 (m, 4H), 3.66-3.62 (m, 4H), 3.54 (t, *J* = 4.8 Hz, 3H), 3.51-3.43 (m, 2H), 3.41-3.25 (m, 9H), 2.86-2.79 (m, 3H), 2.48 (s, 3H), 2.41 (s, 3H), 2.23-2.15 (m, 2H), 1.82-1.74 (m, 3H), 1.47-1.35 (m, 2H); ^{13}**C NMR** (126 MHz, Chloroform-*d*) δ 162.63, 161.84, 161.27, 160.97, 158.32, 144.60, 142.45, 140.52, 138.19, 129.89, 129.24, 126.24, 126.13, 119.49, 117.18, 114.88, 68.81, 68.40, 66.47, 59.22, 59.06, 55.78, 51.63, 48.38, 47.99, 43.87, 31.74, 22.76, 21.67, 21.45; **IR** = 1690, 1563, 1278, 1146, 1089, 819, 682 cm^{-1}; **HRMS** Exact Mass ($C_{36}H_{55}N_7O_9S_3$ + H): calculated = 826.3296, measured = 826.3300.

Procedure for the preparation of (*E*)-*N*-benzyl-*N*-((*E*)-2-(cyclohexylamino)-2-((dibenzylcarbamoyl)imino)ethyl)-*N*'-(dibenzylcarbamoyl)-2-((2-methoxyethyl)(methyl)amino)acetimidamide (33):

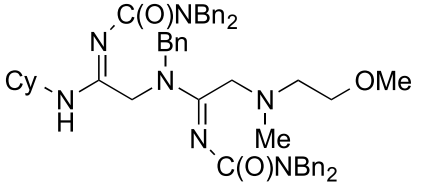

FA

200 mg of cyclohexyl resin with a loading of 0.51 mmol/g was treated under the standard solid phase synthesis procedure. After cleavage, the solution was removed by rotary evaporation and the residual compound was dried under high vacuum. The crude material was loaded onto an automated purification system (Teledyne Isco, Inc.) on a C18 reverse phase column and purified using a 0-100% gradient of acetonitrile in water buffered with 0.1% formic acid. The fractions were combined and the crude material was lyophilized to provide 34 mg of **33** in 39% yield assuming a formic acid salt. ^{1}**H NMR** (400 MHz, Chloroform-*d*) δ 7.81-6.52 (m, 25H), 5.10-4.10 (m, 10H), 4.07-3.71 (m, 1H), 3.50 (t, *J* = 4.7 Hz, 2H), 3.31-3.20 (m, 3H), 3.14-2.81 (m, 2H), 2.67 (t, *J* = 5.0 Hz, 2H), 2.25 (s, 2H), 2.18-2.05 (m, 1H), 1.82 (d, *J* = 12.9 Hz, 2H), 1.75-1.49 (m, 6H), 1.39-1.16 (m, 5H), 1.16-0.91 (m, 3H); ^{13}**C NMR** (126 MHz, Chloroform-*d*) δ 165.23, 163.37, 159.18, 157.10, 139.58, 138.86, 138.29, 138.16, 137.44, 137.13, 132.64, 131.32, 129.41, 129.17, 129.14, 128.78, 128.51, 128.45, 128.40, 128.34, 128.32, 128.23, 128.17, 128.12, 128.03, 128.00, 127.95, 127.72, 127.58, 127.51, 127.43, 127.30, 127.23, 127.20, 127.15, 127.04, 126.78, 126.75, 126.63, 70.34,

58.81, 58.68, 58.40, 56.25, 52.98, 52.51, 51.05, 50.37, 50.18, 49.60, 48.31, 43.88, 42.07, 33.78, 33.65, 31.81, 29.64, 28.41, 25.97, 25.47, 25.14, 24.83, 24.52, 24.29, 19.89; **IR** = 1660, 1594, 1558, 1495, 1451, 1267 1202, 1115, 963, 732, 698 cm^{-1}; **HRMS** Exact Mass ($C_{51}H_{61}N_7O_3$ + H): calculated = 820.4904, measured = 820.4910

Procedure for the preparation of (*E*)-2-(benzyl(1-(2-methoxyethyl)-2-oxo-2,5-dihydro-1*H*-imidazol-4-yl)amino)-*N*-cyclohexyl-*N*'-(methylsulfonyl)acetimidamide (34):

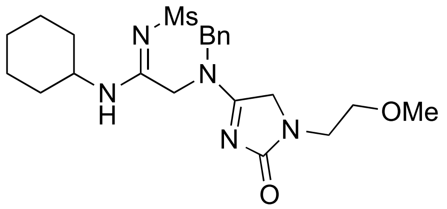

200 mg of cyclohexyl resin with a loading of 0.51 mmol/g was treated under the standard solid phase synthesis procedure. After cleavage, the crude cleavage solution was removed by rotary evaporation and the crude material was loaded onto an automated purification system (Teledyne Isco, Inc.) on a reverse-phase C18 column and purified using a 0-60% gradient of acetonitrile in water using a 0.1% formic acid buffer. The fractions containing the product were combined, however they still contained a residual impurity. These fractions were lyophilized and were subsequently loaded onto an automated purification system (Teledyne Isco, Inc.) on a normal phase column and purified using a 0-5% gradient of MeOH in dichloromethane. The fractions containing the product were combined to give 23.7 mg of **34** in 42% yield. **^{1}H NMR** (500 MHz, Acetonitrile-d_3) δ 7.54-7.16 (m, 5H), 4.83-4.06 (m, 6H), 3.80-3.66 (m, 1H), 3.50-3.42 (m, 4H), 3.33-3.23 (m, 3H), 2.90-2.82 (m, 3H), 2.57-2.01 (m, 1H), 1.87-1.48 (m, 5H), 1.39-1.02 (m, 5H); **^{13}C NMR** (126 MHz, Acetonitrile-d_3) δ 179.04, 178.77, 169.05, 168.28, 163.16, 162.69, 162.22, 161.41, 136.00, 129.96, 129.84, 129.68, 129.59, 129.38, 129.08, 128.65, 128.48, 71.71, 58.74, 54.40, 53.33, 52.55, 52.24, 52.00, 51.31, 49.97, 48.74, 43.59, 43.52, 42.74, 42.65, 42.13, 33.84, 32.04, 26.14, 25.74, 25.25; **IR** = 1544, 1447, 1371, 1270, 1135, 1083, 1051, 811, 660, 548 cm^{-1}; Exact Mass ($C_{22}H_{33}N_5O_4S$ + H): calculated = 464.2327, measured = 464.2337.

Procedure for the preparation of 3-((*E*)-2-((*E*)-*N*-(4-aminobutyl)-2-(((*R*)-1-phenylethyl)amino)-*N*'-tosylacetimidamido)-*N*-((*E*)-2-(cyclohexylamino)-2-(tosylimino)ethyl)-*N*'-(methylsulfonyl)acetimidamido)propanoic acid (35):

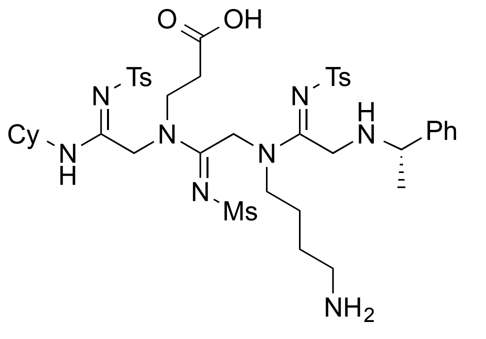

FA

200 mg of cyclohexyl resin with a loading of 0.51 mmol/g was treated under the standard solid phase synthesis procedure. The final amination using (S)-methylbenzyl amine was carried out at 60° C in the microwave for 14 hours. The resin was cleaved using 95:5 DCM:TFA for 1 hour and them the cleavage solution was rotovaped. To this 2 mL of 95:2.5:2.5 TFA/H_2O/TIPS was added for 10 minutes in order to fully deprotect the side chains followed by the addition of 30 mL of water and lyophilization. The crude material was then redissolved in water/acetonitrile 1:1 and was loaded onto an automated purification system (Teledyne Isco, Inc.) on a C18 reverse phase column using a 0-55% gradient of acetonitrile in water buffered with 0.1% formic acid. The fractions were combined and lyophilized to provide 24 mg of **35** in 25% yield assuming a single formate

salt. **^{1}H NMR** (500 MHz, Acetonitrile-*d_3*) δ 9.70-9.44 (m, 1H), 7.99-7.67 (m, 4H), 7.56-7.25 (m, 9H), 4.57-4.07 (m, 2H), 4.05-3.46 (m, 7H), 3.31 (d, *J* = 12.4 Hz, 1H), 3.26-2.93 (m, 3H), 2.85-2.63 (m, 3H), 2.63-2.28 (m, 9H), 2.01-1.61 (m, 12H), 1.55-1.12 (m, 11H); **^{13}C NMR** (126 MHz, Acetonitrile-*d_3*) δ 179.49, 168.39, 165.05, 163.90, 146.00, 144.32, 143.54, 143.36, 141.50, 130.85, 130.66, 130.03, 129.89, 128.58, 128.15, 127.86, 127.34, 111.37, 60.28, 54.38, 52.45, 52.13, 51.64, 50.18, 47.68, 44.83, 43.97, 40.15, 37.34, 32.85, 27.10, 26.69, 26.32, 25.86, 24.68, 22.05, 21.87; **IR** = 1539, 1265, 1141, 1087, 733, 695, 523 cm^{-1}; **HRMS** Exact Mass ($C_{42}H_{60}N_8O_8S_3$ + H): calculated = 901.3769, measured = 901.3756; **$[\alpha]_D^{25}$** = -19.0° (c 0.01, CH-$_3$OH).

Procedure for the preparation of (*R*)-3-((*E*)-2-((*E*)-*N*-(2-methoxyethyl)-*N*'-(methylsulfonyl)-2-(pyrrolidin-1-yl)acetimidamido)-*N*-(tetrahydro-2*H*-pyran-4-yl)-*N*' tosylacetimidamido)butanamide (36):

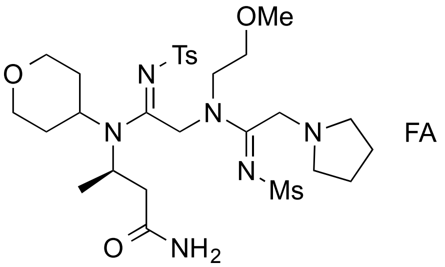

200 mg of rink resin with a substitution of 0.51 mmol/g was deprotected 2x with 20% piperdine in DMF (10 mL) for 20 minutes followed by washing of the resin. Fmoc-L-beta-homoalanine, diisopropylcarbodiimine, hydroxylbenzole-triazole and diisopropylamine were all dissolved to 0.1 M in DMF, and 4 mL of this solution was added to the resin for 1 hour. The coupling was then repeated. The resin was then washing the resin 3x with 10 mL of DMF. The Fmoc group washed with excess DMF and treated with 10 mL of 20% piperdine in DMF 2x and then the resin was washed 3x with DMF followed by 3x with DCM and was dried under high vacuum. Reductive amination with was carried out as described in solid phase procedures. The resin was then treated with standard solid phase peptidine synthesis procedures. The product was cleaved with 10 ml of 95:5 TFA/H_2O for 1 hour. After cleavage, the solution was diluted into 30 mL of water and lyophilized. The crude material was loaded onto an automated purification system (Teledyne Isco, Inc.) on a C-18 reverse phase column and purified using a 0-100% gradient of acetonitrile in water buffered with 0.1% formic acid. The fractions were combined and lyophilized to provide 34 mg of **36** in 48% yield assuming a formic acid salt. **^{1}H NMR** (501 MHz, Chloroform-*d*) δ 7.76-7.70 (m, 2H), 7.30-7.22 (m, 2H), 5.86-5.10 (m, 2H), 4.99-4.89 (m, 1H), 4.82-4.74 (m, 1H), 4.55 (s, 1H), 4.22 (s, 1H), 4.12-3.88 (m, 7H), 3.65 (s, 2H), 3.50-3.41 (m, 1H), 3.35-3.28 (m, 3H), 3.22 (s, 2H), 3.05-2.96 (m, 3H), 2.67 (s, 3H), 2.42-2.35 (m, 4H), 2.09-1.84 (m, 2H), 1.73 (s, 4H), 1.25 (d, *J* = 7.3 Hz, 4H); **^{13}C NMR** (126 MHz, Chloroform-*d*) δ 172.50, 171.82, 161.26, 142.52, 140.80, 129.40, 129.24, 125.94, 125.76, 71.16, 67.78, 67.49, 66.73, 66.55, 58.98, 56.96, 55.74, 53.83, 52.98, 52.44, 50.86, 49.50, 48.41, 43.72, 43.35, 40.74, 40.14, 30.74, 30.34, 29.15, 28.77, 23.62, 21.45, 18.72, 18.57; **IR** = 1600, 1545, 1452, 1278, 1144, 1087, 750, 696, 578 cm^{-1}; **HRMS** Exact Mass ($C_{28}H_{46}N_6O_7S_2$ + H): calculated = 643.2942, measured = 643.2937; **$[\alpha]_D^{25}$** = +11.0° (c 0.01, CH_2Cl_2).

Procedure for the preparation of (*E*)-2-chloro-*N*-cyclohexyl-*N*'-tosylacetimidamide (41):

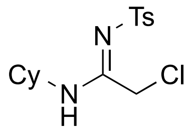

200 mg of cyclohexyl resin with a loading of 0.62 mmol/g was treated under the standard solid phase synthesis procedure. The crude cleavage solution was poured into a separatory funnel containing 10 mL of DCM and was washed

twice with saturated $NaHCO_3$ (10 mL). The combined organic layers were dried over sodium sulfate and the crude material was loaded onto silica gel and was purified using an automated purification system (Teledyne Isco, Inc.) on a normal phase column using a 0-40% gradient of ethylacetate in hexanes to provide 37 mg of **41** in 90% yield. **^{1}H NMR** (400 MHz, Chloroform-*d*) δ 7.84-7.76 (m, 2H), 7.31-7.23 (m, 2H), 6.52 (s, 1H), 4.88 (s, 2H), 3.85 (m 1H), 2.41 (s, 3H), 1.98-1.86 (m, 2H), 1.81-1.54 (m, 3H), 1.45-1.13 (m, 6H); **^{13}C NMR** (101 MHz, Chloroform-*d*) δ 158.99, 142.46, 140.09, 129.26, 126.23, 50.81, 42.29, 31.68, 25.28, 24.30, 21.53, 21.48; **IR** = 1549 1452, 1276 1116, 965 780, 733, 699, 561, 519 cm^{-1}; **HRMS** Exact Mass ($C_{15}H_{21}ClN_2O_2S$ + H): calculated = 329.1085, measured = 329.1094.

7. **Calculations:**

We performed quantum chemical energy calculations in order to determine whether the *trans* 0r *cis* amidine structure is more stable. First, we used 2D Sketcher and MacroModel with OPLS 2005 force field to construct and optimize the entry structures. For each of the compounds, we set the dihedral angles to 0° and 180° for *cis*- and *trans*- initial structures, respectively (see below). We optimized *Cis*- and *Trans*- initial structures of the peptidines with Jaguar. DFT with B3LYP functional and 6-31G** basis set were used. The parts of the molecules other than R-group were constrained. We determined which structure is more stable by comparing the energies after the optimizations.

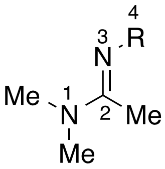

trans
Dihedral (1,2,3,4) = 180

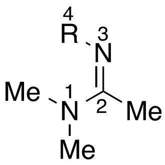

cis
Dihedral (1,2,3,4) = 0

8. Crystallography:

To 10 mg of **28** as the TFA salt DCM (10 mL) was added followed by and 2M HCl in diethyl ether (200 uL) and the solution was rotovaped. This process was repeated 3x. The residual material was dissolved in acetonitrile/water and 1 mL of 1 M HCl was added, this solution was frozen and lyophilized removing any remaining TFA. Crystallization in 1:1 DCM/Hexanes provided crystals amendable to diffraction. We were not able to crystalized **28** as a freebase.

Table 1. Crystal data and structure refinement for **28**.

Identification code	12068	
Empirical formula	C49 H60 Cl N7 O6 S3	
Formula weight	974.67	
Temperature	93(2) K	
Wavelength	1.54187 Å	
Crystal system	Monoclinic	
Space group	P 21/c	
Unit cell dimensions	a = 12.9375(2) Å	a= 90°.
	b = 11.6146(2) Å	b= 94.227(7)°.
	c = 32.205(2) Å	g = 90°.
Volume	4826.0(4) $Å^3$	
Z	4	
Density (calculated)	1.341 Mg/m^3	
Absorption coefficient	2.373 mm^{-1}	
F(000)	2064	
Crystal color	?	
Crystal size	0.150 x 0.050 x 0.030 mm^3	
Theta range for data collection	2.752 to 65.089°	
Index ranges	-14 <= h <= 15, -13 <= k <= 13, -37 <= l <= 37	
Reflections collected	68998	
Independent reflections	8226 [R(int) = 0.1222]	
Completeness to theta = 67.687°	94.3 %	
Refinement method	Full-matrix least-squares on F^2	
Data / restraints / parameters	8226 / 2 / 605	
Goodness-of-fit on F^2	1.086	
Final R indices [I>2sigma(I) = 5884 data]	R1 = 0.0697, R2 = 0.1864	
R indices (all data, ? Å)	R1 = 0.0909, R2 = 0.2067	
Extinction coefficient	0.00144(18)	
Largest diff. peak and hole	0.655 and -0.540 $Å^{-3}$	

9. References:

(1) Yates, M. H.; Kallman, N. J.; Ley, C. P.; Wei, J. N. *Org Process Res Dev* 2009, *13*, 255.

(2) Ma, L. A.; Xie, C. F.; Ma, Y. H.; Liu, J. A.; Xiang, M. L.; Ye, X.; Zheng, H.; Chen, Z. Z.; Xu, Q. Y.; Chen, T.; Chen, J. Y.; Yang, J. C.; Qiu, N.; Wang, G. C.; Liang, X. L.; Peng, A. H.; Yang, S. Y.; Wei, Y. Q.; Chen, L. J. *J Med Chem* 2011, *54*, 2060.

(3) Cuenca, F.; Moore, M. J. B.; Johnson, K.; Guyen, B.; De Cian, A.; Neidle, S. *Bioorg Med Chem Lett* 2009, *19*, 5109.

(4) Brown, E. G.; Nuss, J. M. *Tetrahedron Lett* 1997, *38*, 8457.

(5) Moura-Letts, G.; DiBlasi, C. M.; Bauer, R. A.; Tan, D. S. *P Natl Acad Sci USA* 2011, *108*, 6745.

10. Catalog of ^{1}H, ^{13}C and Analytical HPLC Traces: Solution Phase

Due to the broad peaks observed in both ^{1}H and ^{13}C NMR spectra of numerous oligomeric peptidines, we further demonstration of purity of these compounds by running analytical HPLC. All analytical HPLCs were run from 0-100% over 40 minutes using water/acetonitrile with 0.1% TFA with the exception of compound **36**, where FA was used instead of TFA.

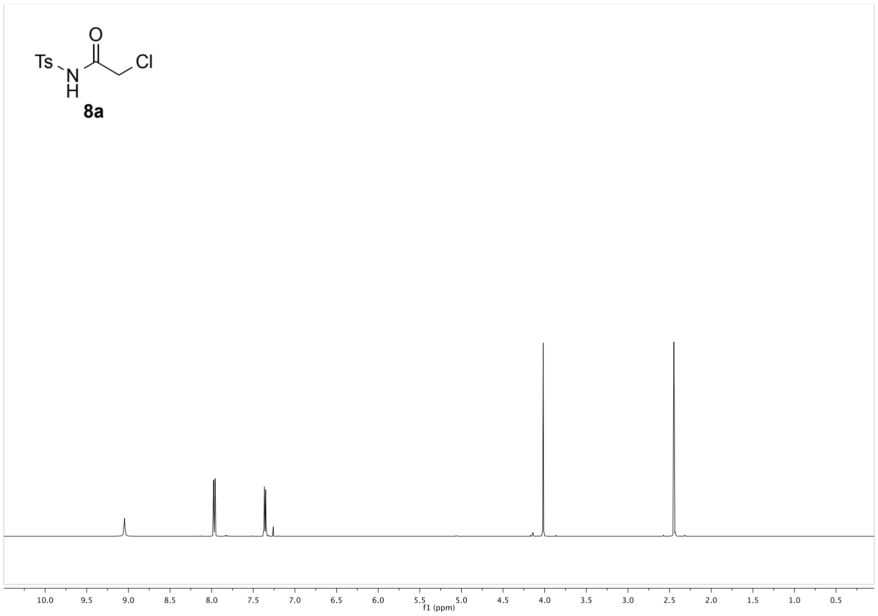

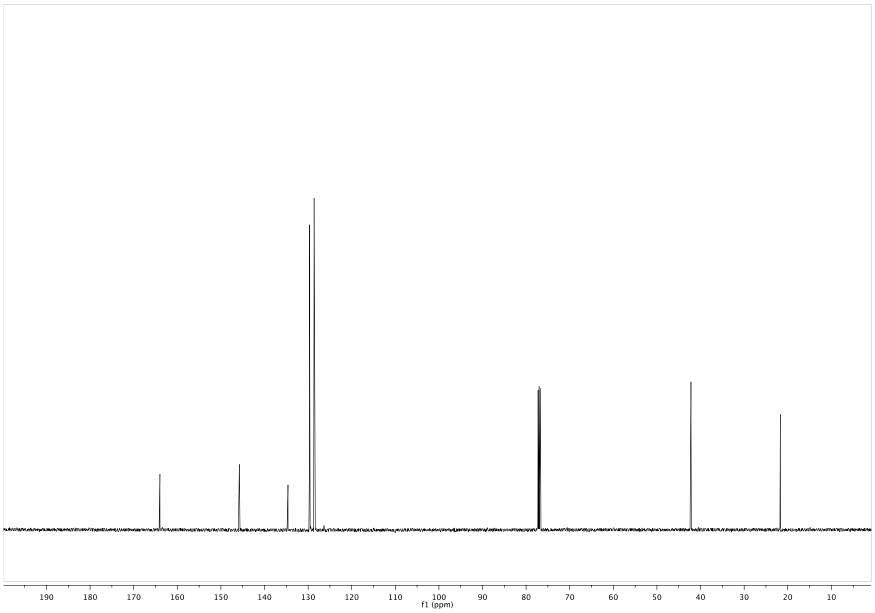

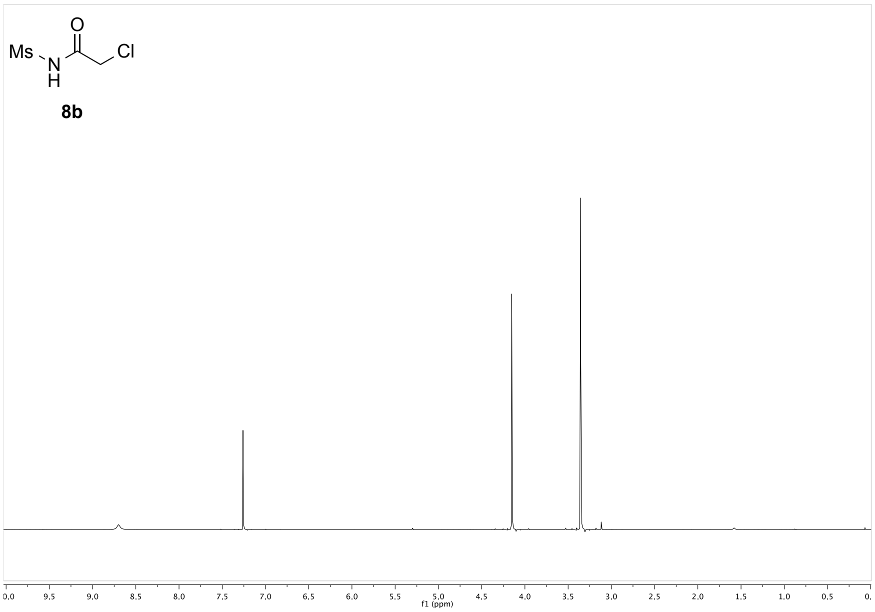
Ms
N
H
O
Cl
8b
f1 (ppm)

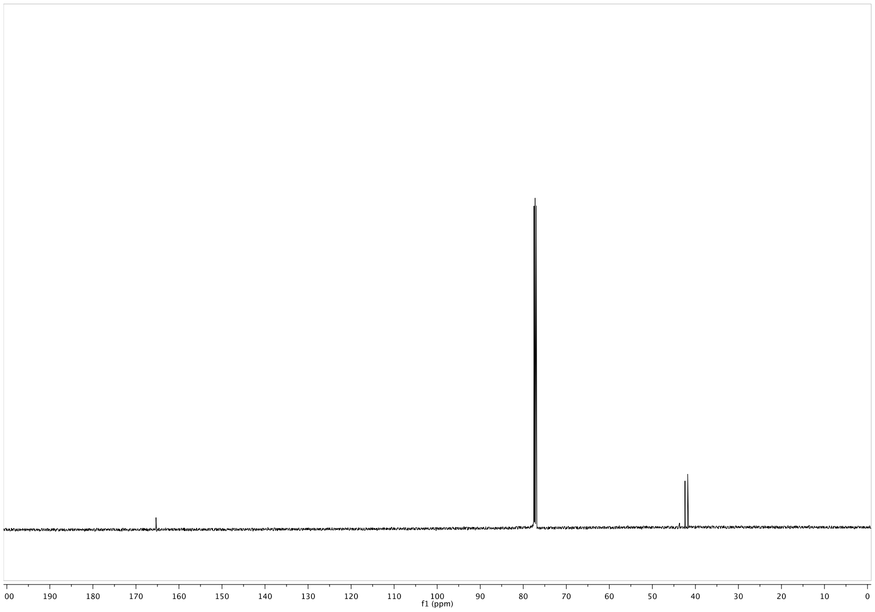
f1 (ppm)

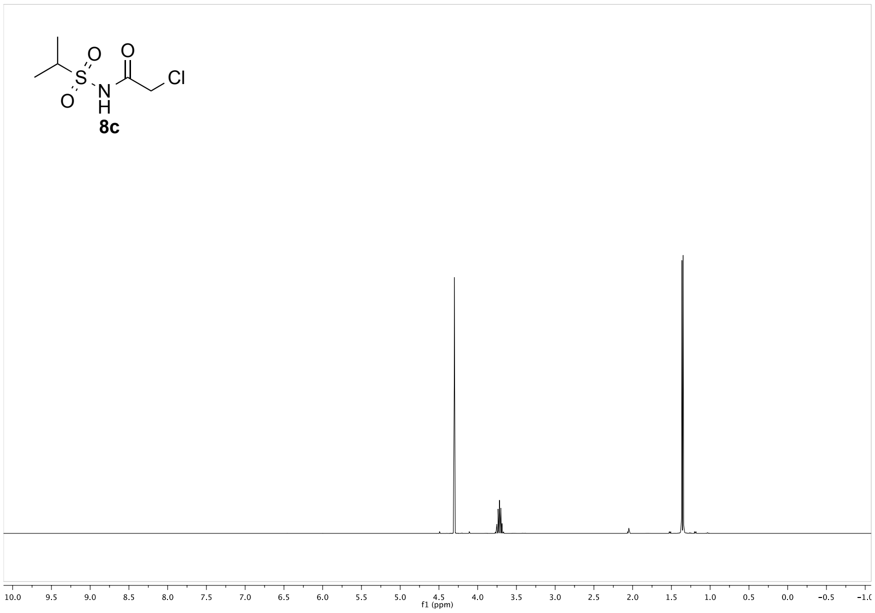

O
S
O
N
H
O
Cl
8c
f1 (ppm)

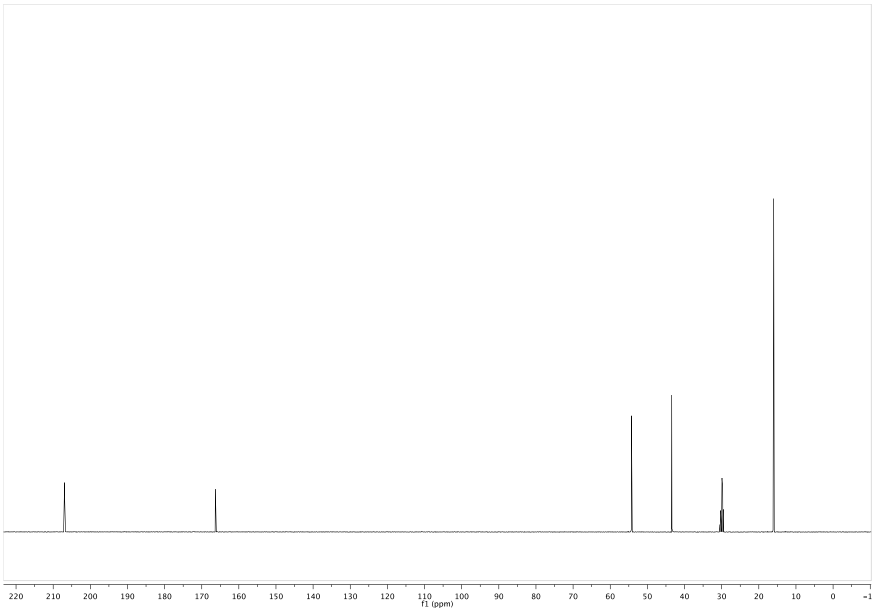

f1 (ppm)

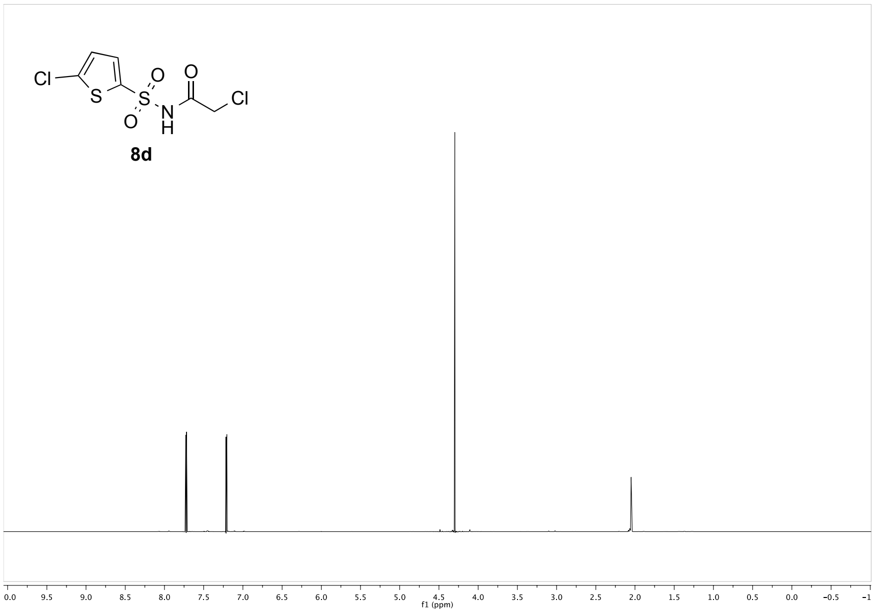
Cl
S
O
O
O
N
H
Cl
8d
f1 (ppm)

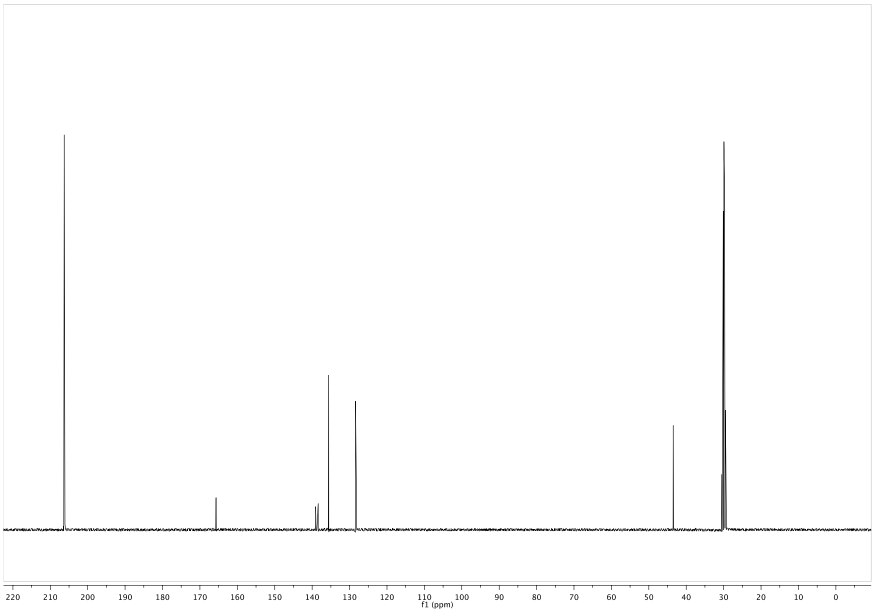
f1 (ppm)

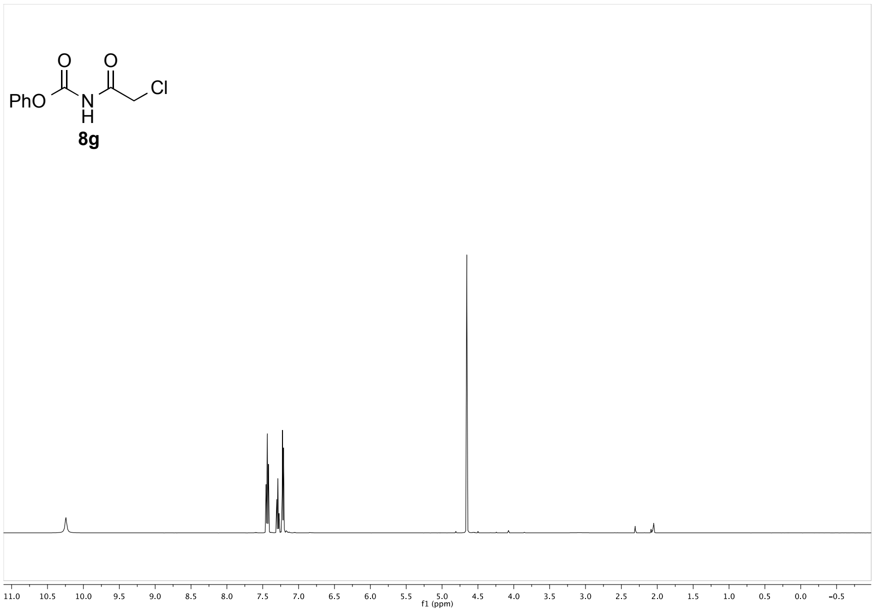
PhO
N
H
Cl
8g
f1 (ppm)

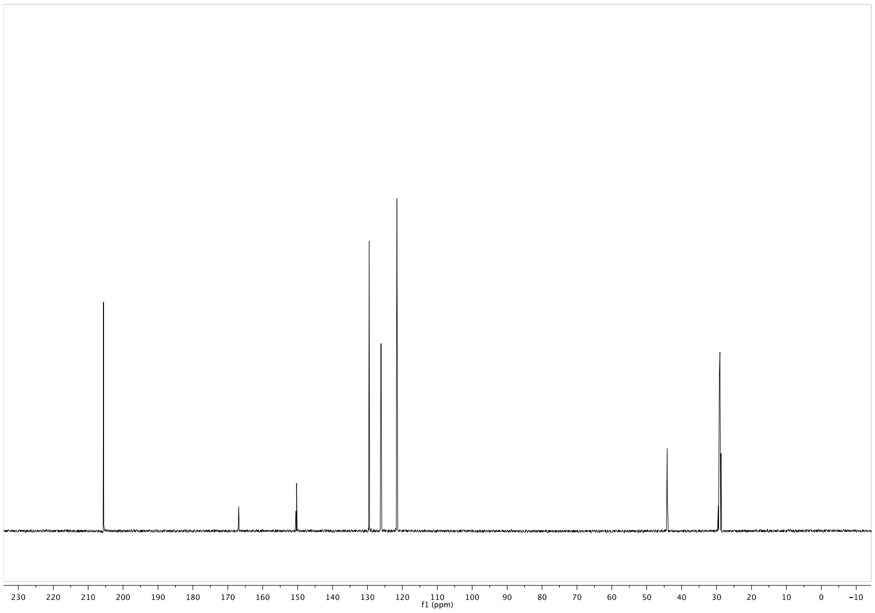
f1 (ppm)

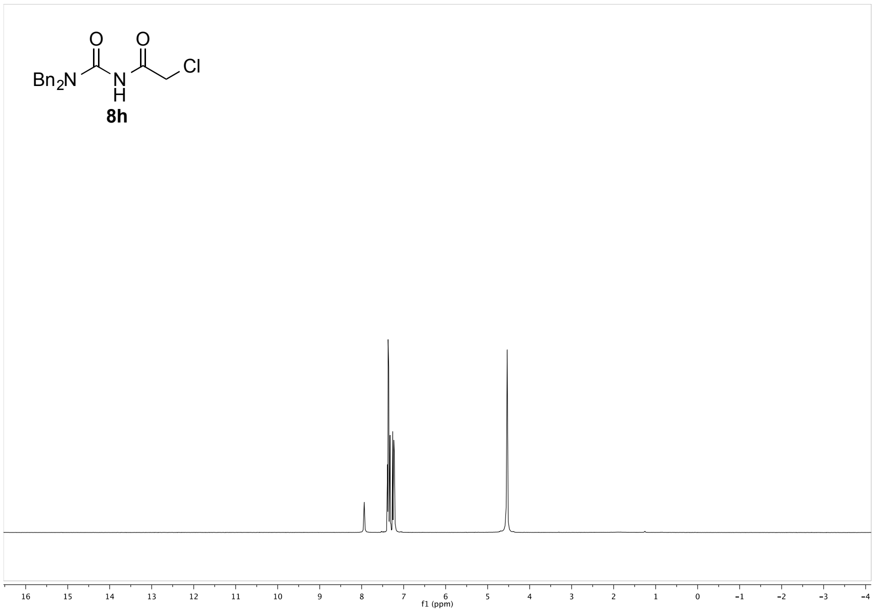
Bn2N
O
O
N
H
Cl
8h
f1 (ppm)

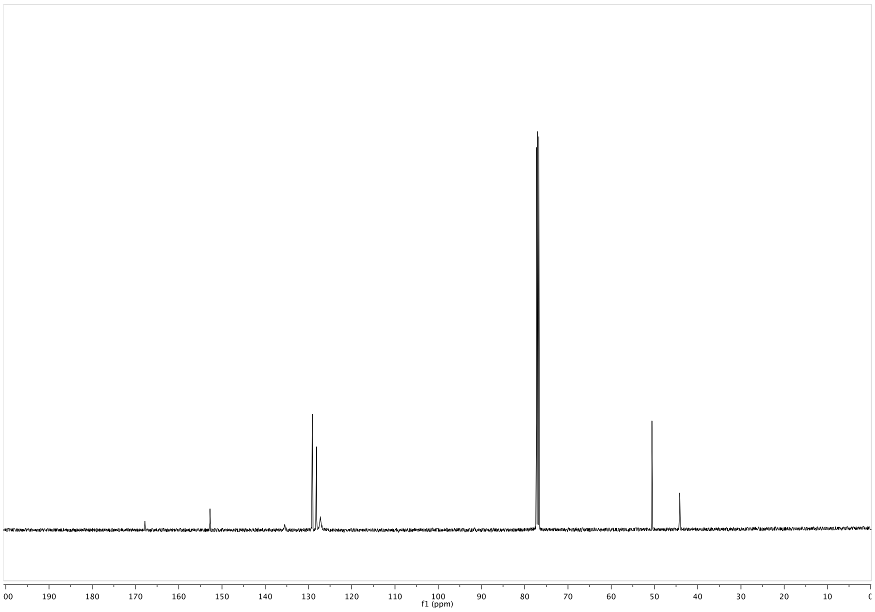
f1 (ppm)

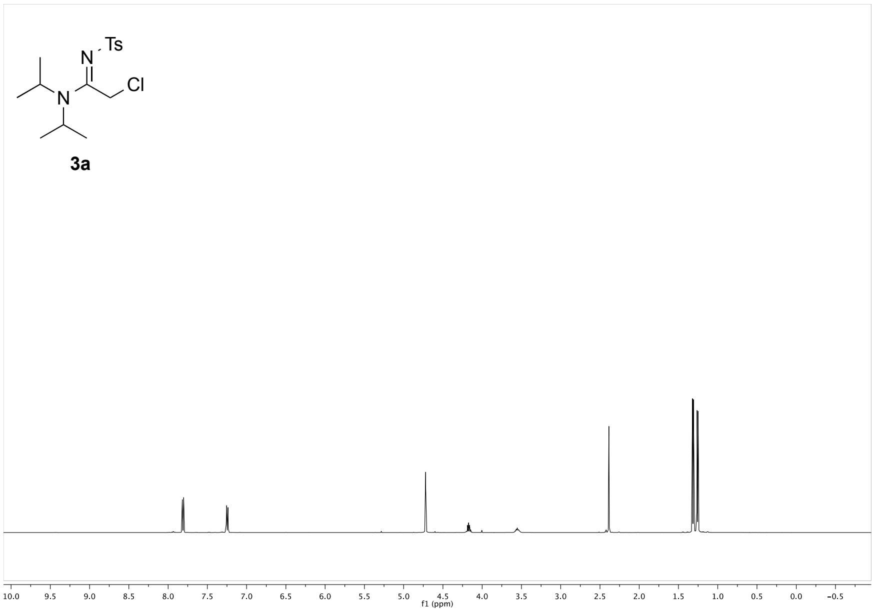
N
Ts
N
Cl
3a
10.0 9.5 9.0 8.5 8.0 7.5 7.0 6.5 6.0 5.5 5.0 4.5 4.0 3.5 3.0 2.5 2.0 1.5 1.0 0.5 0.0 -0.5
f1 (ppm)

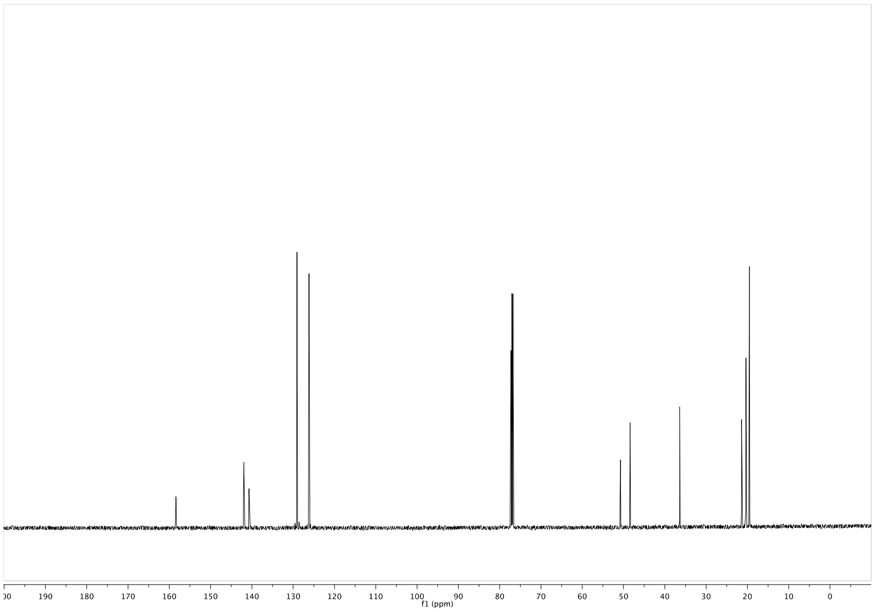
190 180 170 160 150 140 130 120 110 100 90 80 70 60 50 40 30 20 10 0
f1 (ppm)

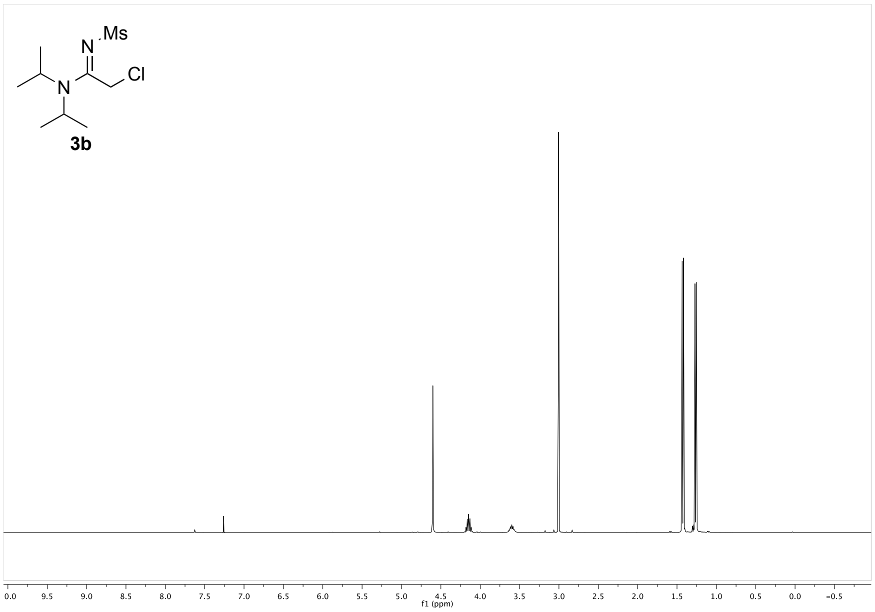

N
Ms
N
Cl
3b
10.0 9.5 9.0 8.5 8.0 7.5 7.0 6.5 6.0 5.5 5.0 4.5 4.0 3.5 3.0 2.5 2.0 1.5 1.0 0.5 0.0 -0.5
f1 (ppm)

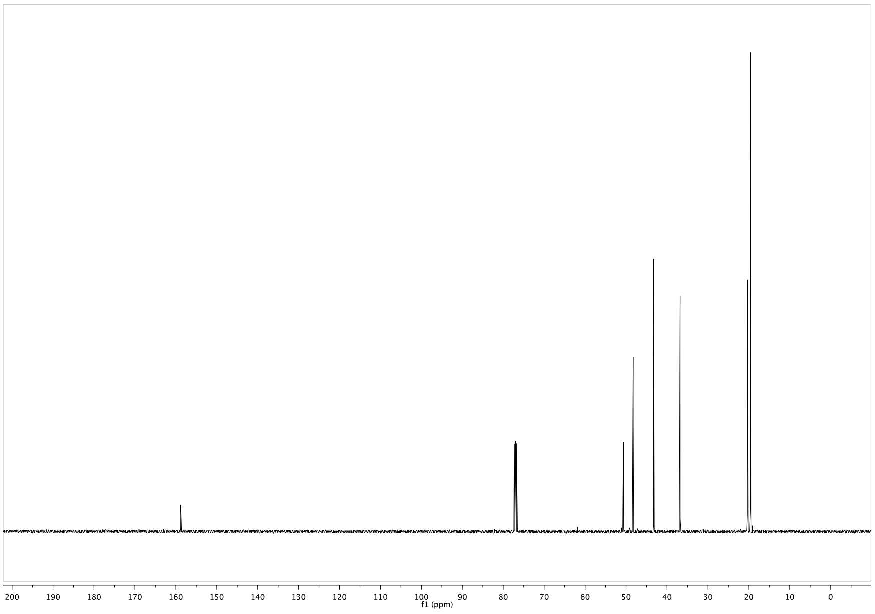

200 190 180 170 160 150 140 130 120 110 100 90 80 70 60 50 40 30 20 10 0
f1 (ppm)

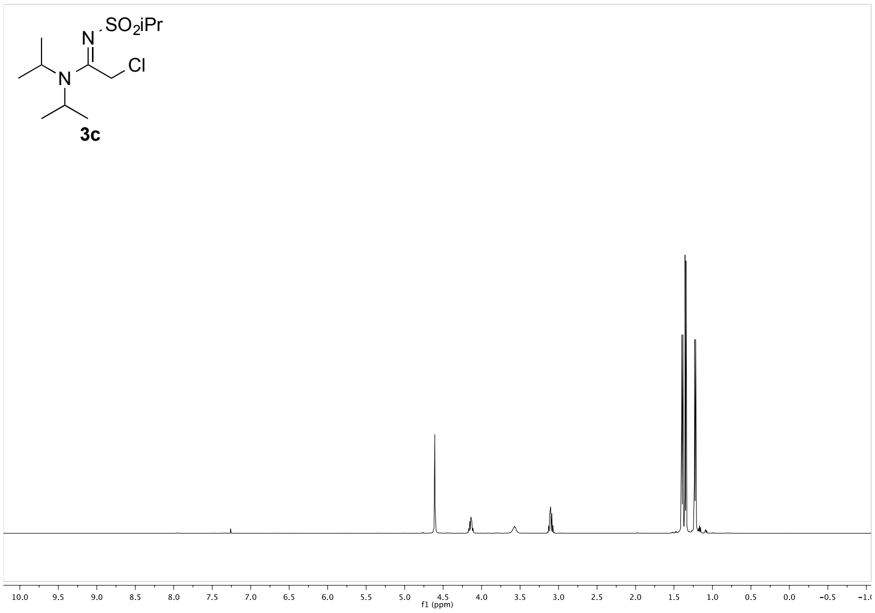

SO2iPr
N
N
Cl
3c
f1 (ppm)

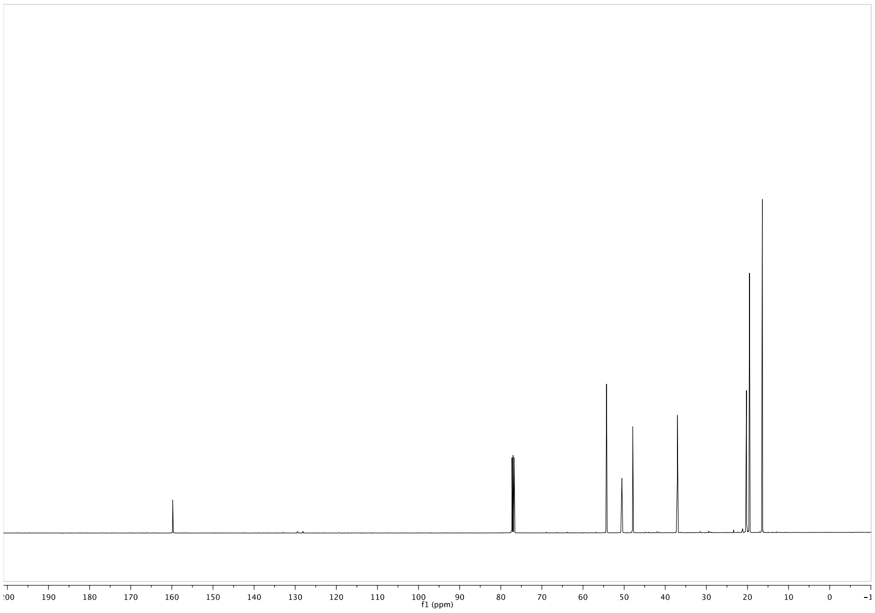

f1 (ppm)

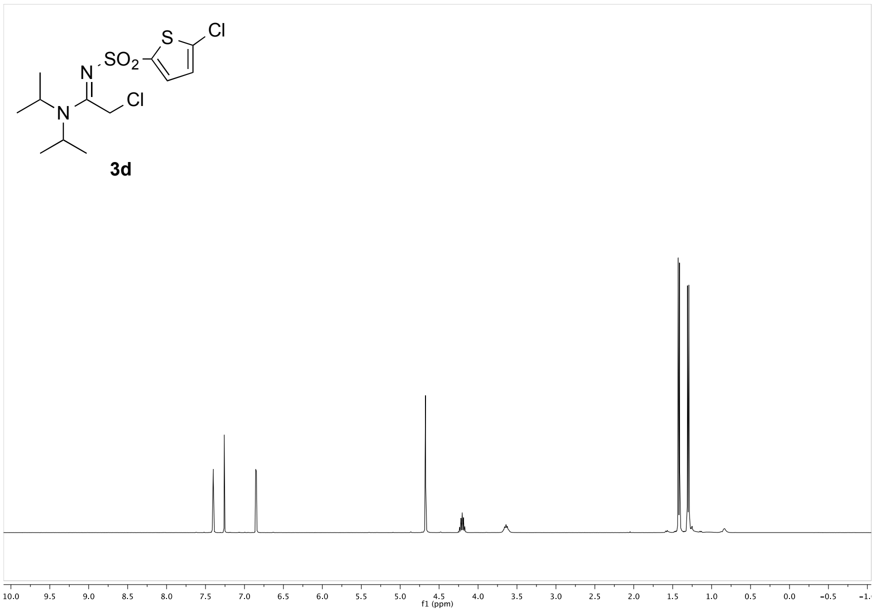

S
Cl
N
SO2
N
Cl
3d
f1 (ppm)

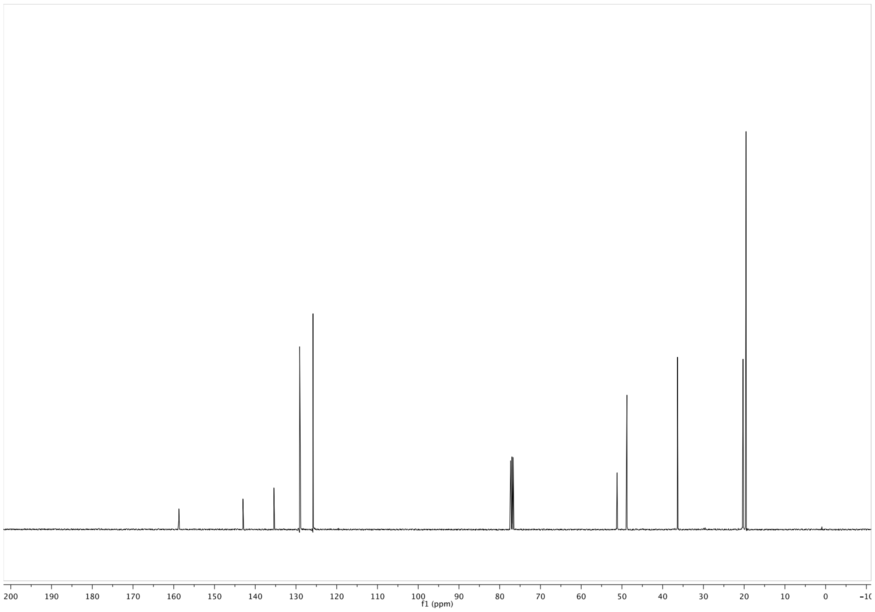

f1 (ppm)

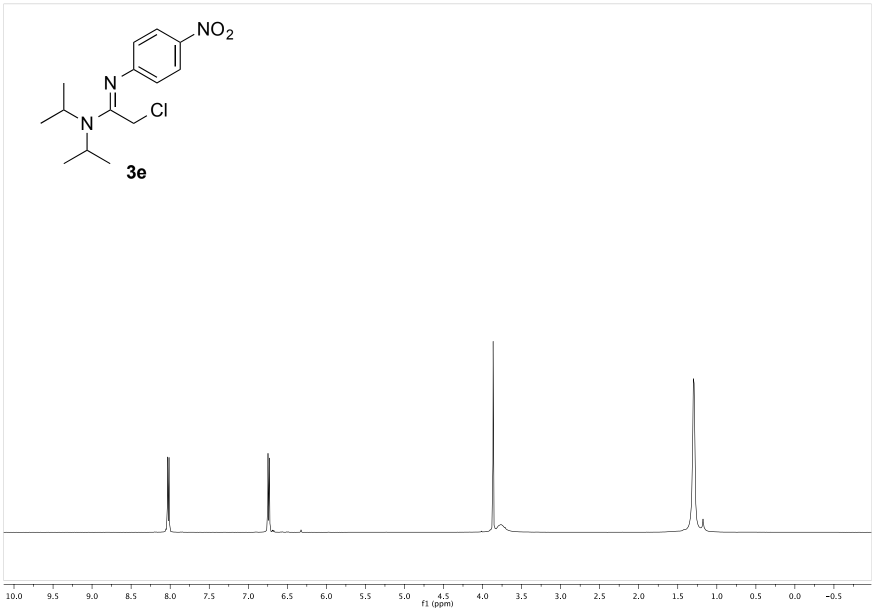
NO2
N
N
Cl
3e
f1 (ppm)

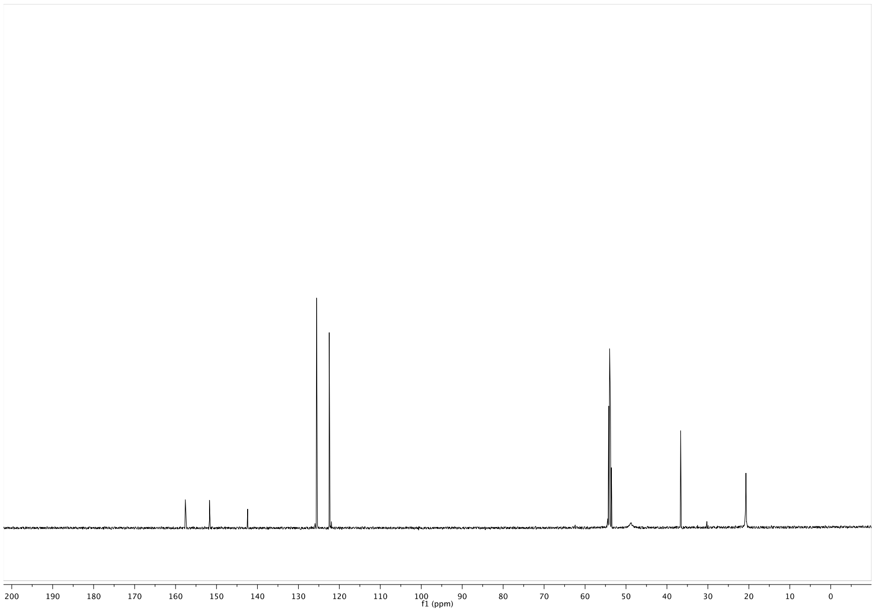
f1 (ppm)

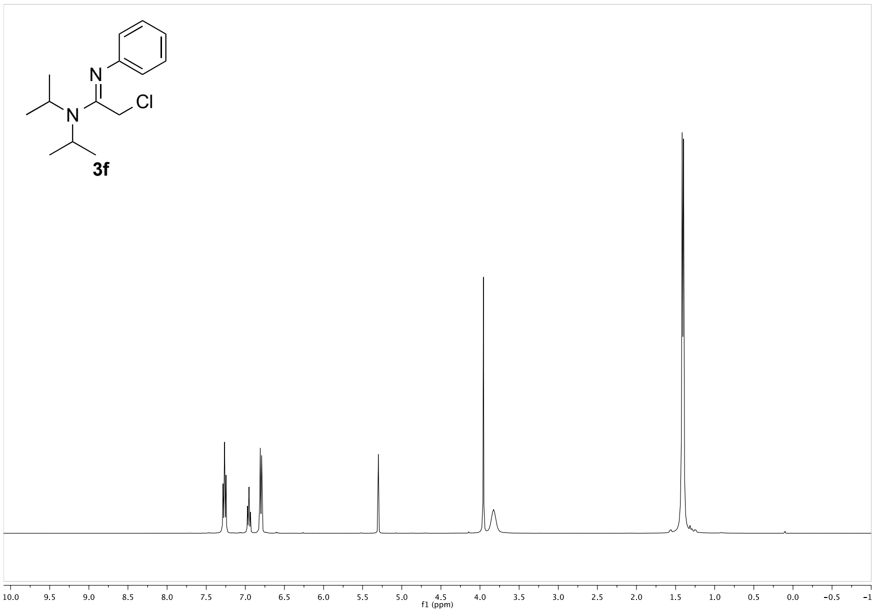
N
N
Cl
3f
10.0 9.5 9.0 8.5 8.0 7.5 7.0 6.5 6.0 5.5 5.0 4.5 4.0 3.5 3.0 2.5 2.0 1.5 1.0 0.5 0.0 -0.5 -1
f1 (ppm)

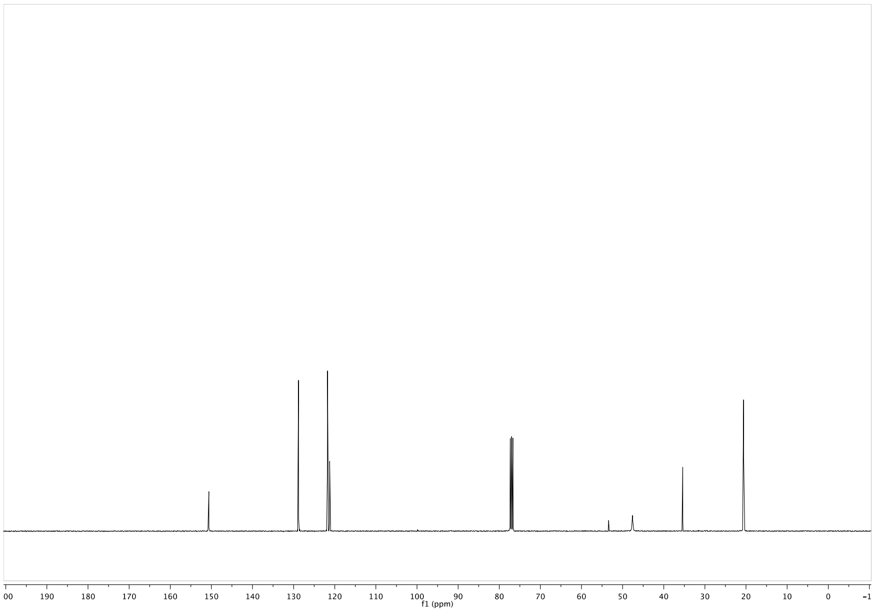
00 190 180 170 160 150 140 130 120 110 100 90 80 70 60 50 40 30 20 10 0 -1
f1 (ppm)

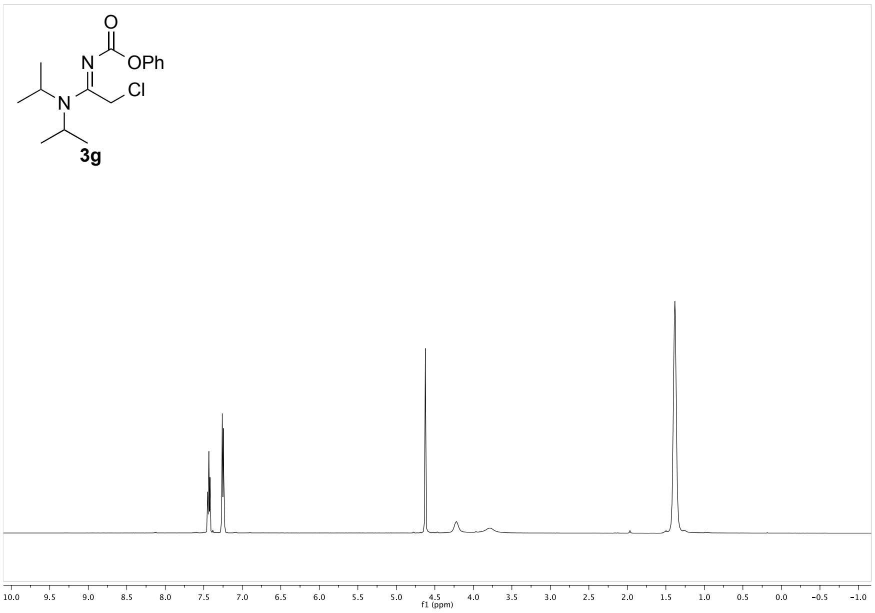
O
N
OPh
N
Cl
3g
f1 (ppm)

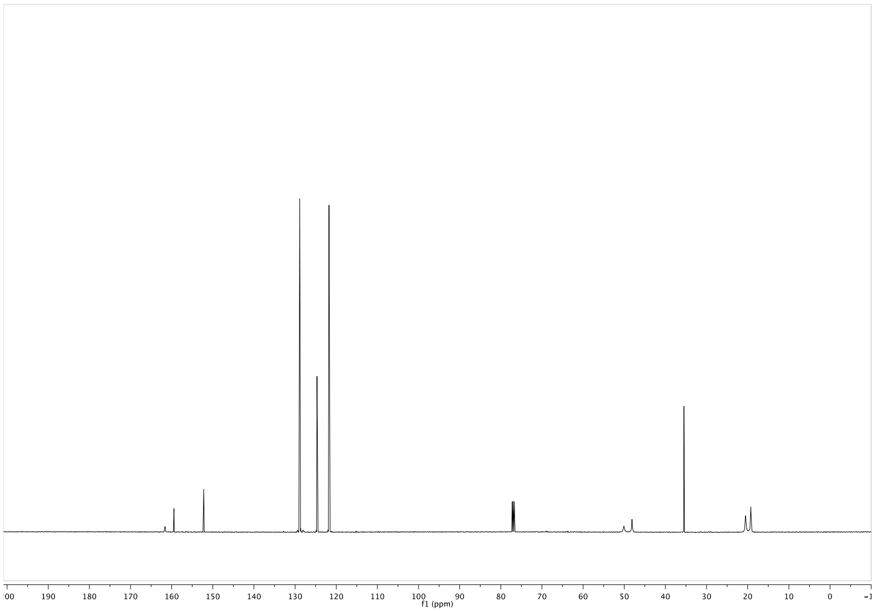
f1 (ppm)

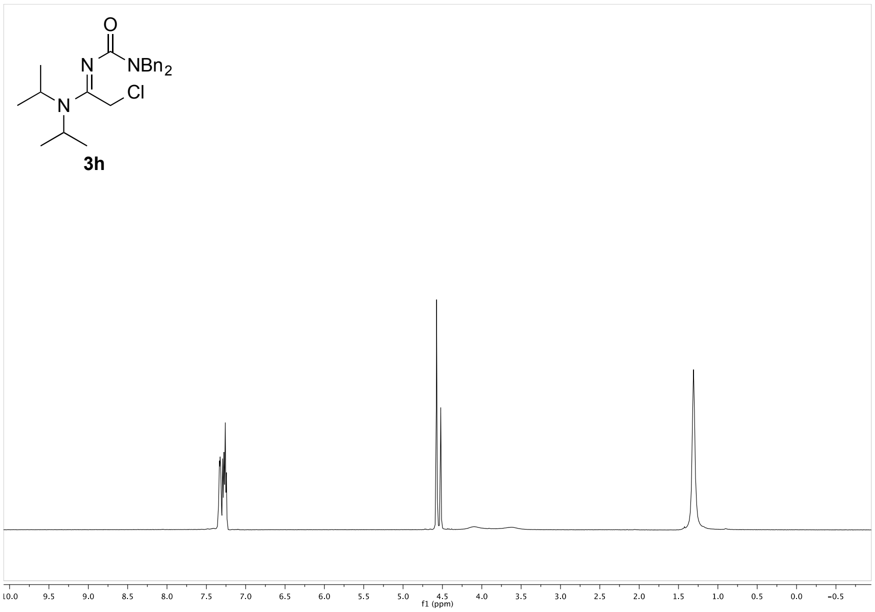
O
N
NBn2
N
Cl
3h
f1 (ppm)

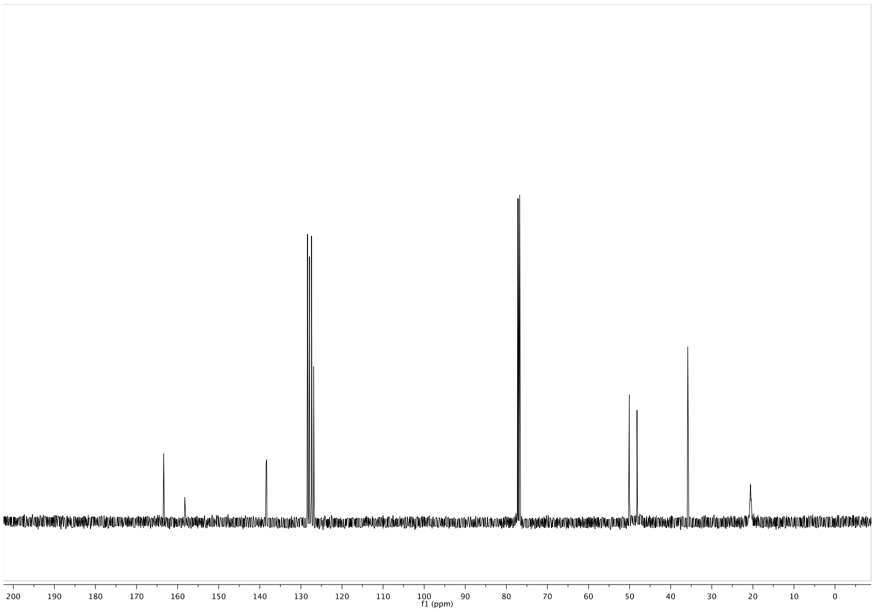
f1 (ppm)

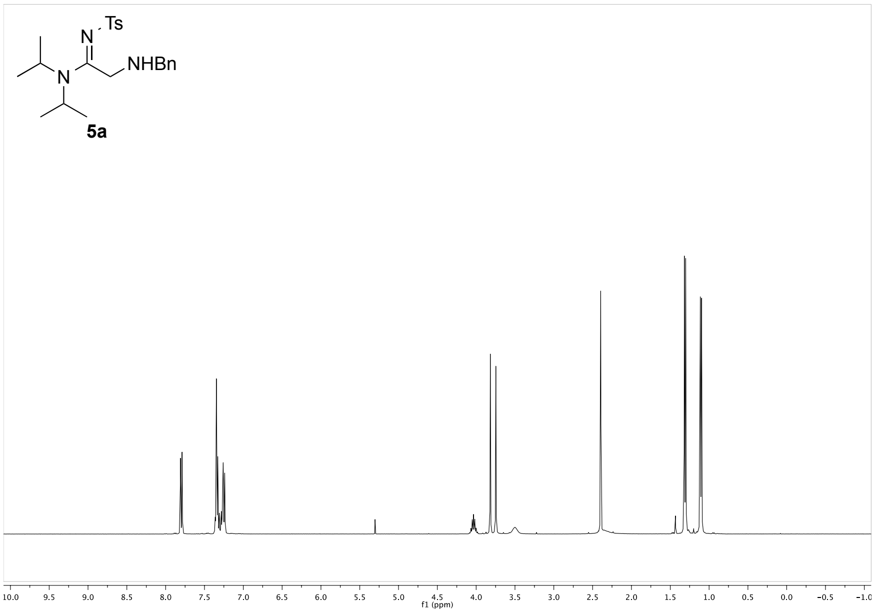
Ts
N
N
NHBn
5a
f1 (ppm)

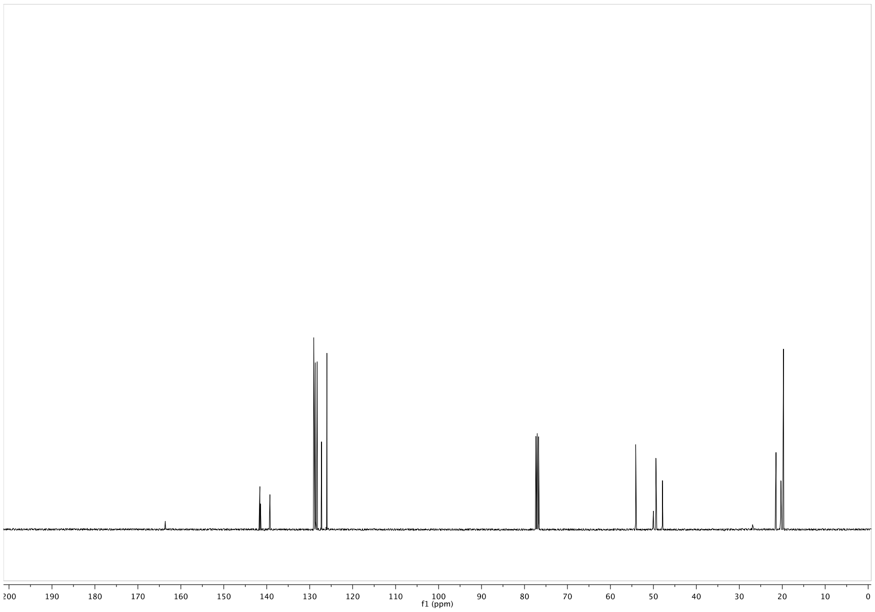
f1 (ppm)

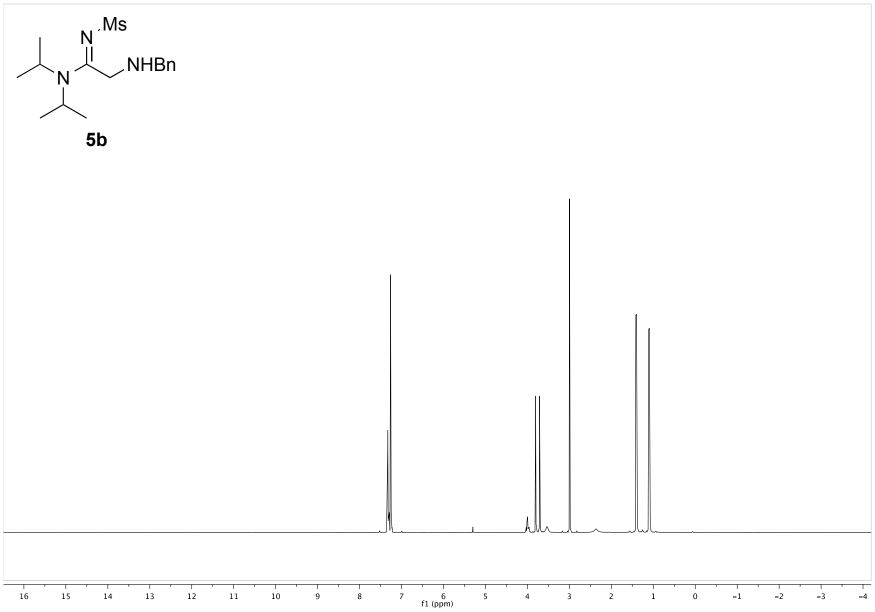
Ms
N
N
NHBn
5b
16 15 14 13 12 11 10 9 8 7 6 5 4 3 2 1 0 −1 −2 −3 −4
f1 (ppm)

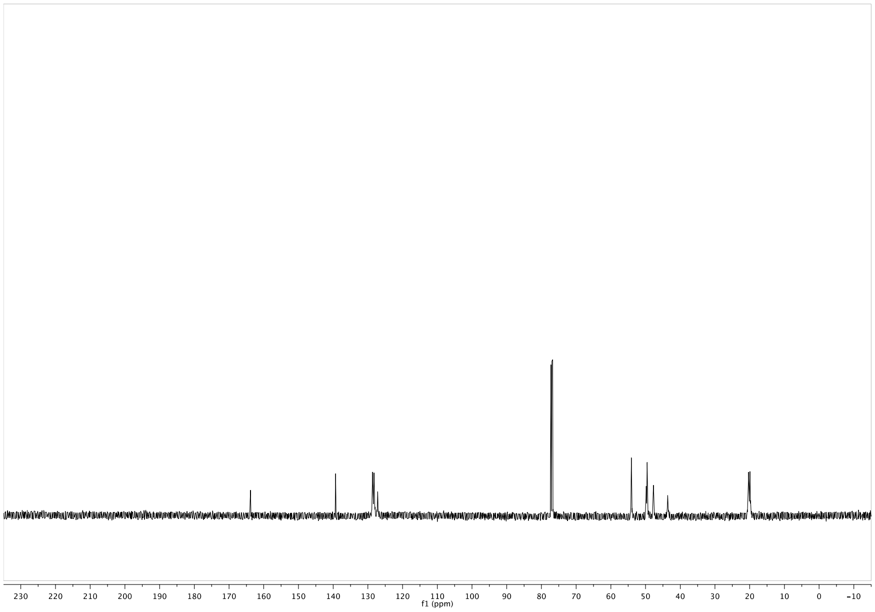
230 220 210 200 190 180 170 160 150 140 130 120 110 100 90 80 70 60 50 40 30 20 10 0 −10
f1 (ppm)

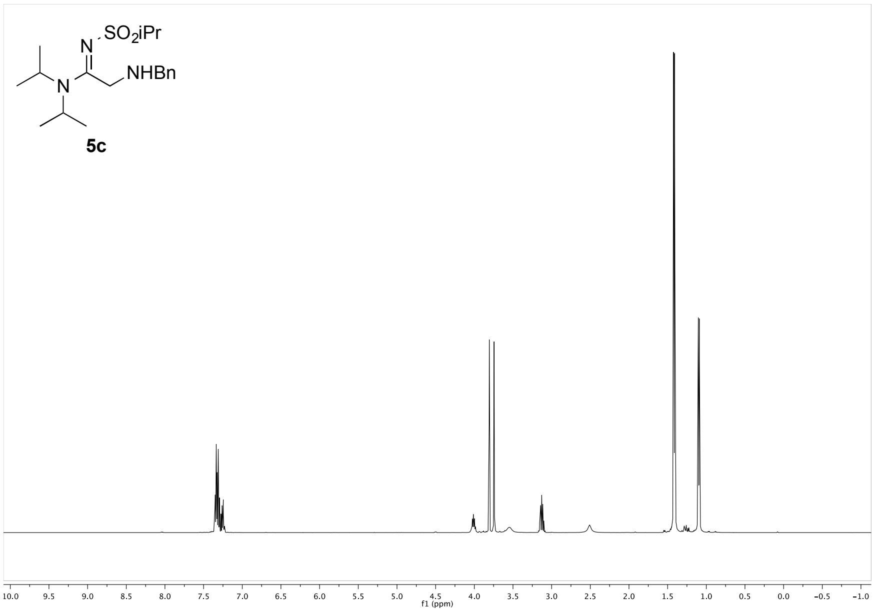

N
SO2iPr
NHBn
N
5c
10.0 9.5 9.0 8.5 8.0 7.5 7.0 6.5 6.0 5.5 5.0 4.5 4.0 3.5 3.0 2.5 2.0 1.5 1.0 0.5 0.0 -0.5 -1.0
f1 (ppm)

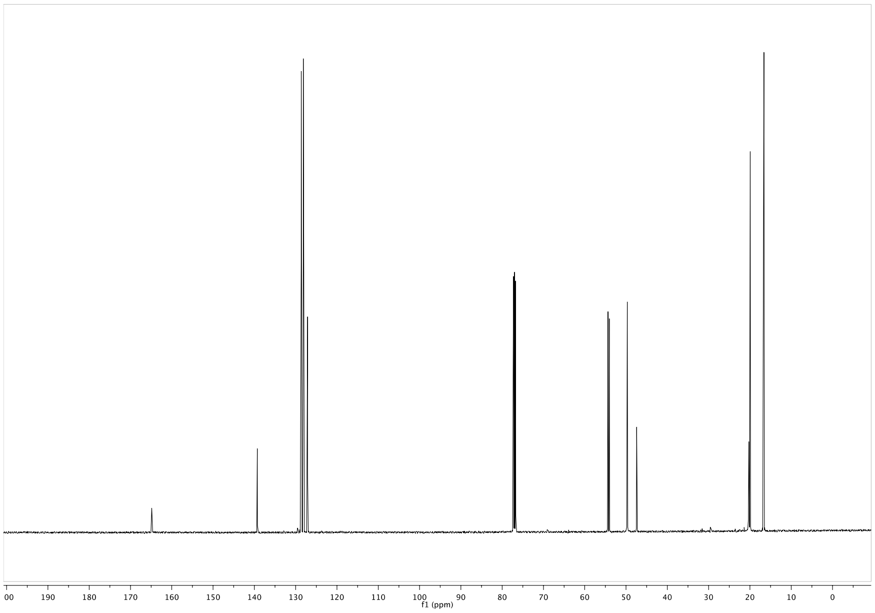

00 190 180 170 160 150 140 130 120 110 100 90 80 70 60 50 40 30 20 10 0
f1 (ppm)

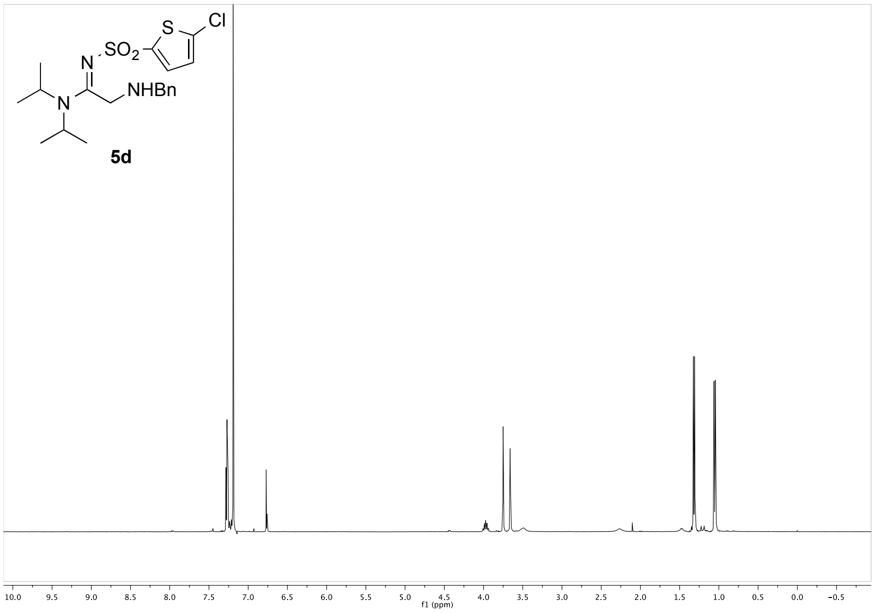
N
SO2
S
Cl
N
NHBn
5d
10.0
9.5
9.0
8.5
8.0
7.5
7.0
6.5
6.0
5.5
5.0
4.5
4.0
3.5
3.0
2.5
2.0
1.5
1.0
0.5
0.0
-0.5
f1 (ppm)

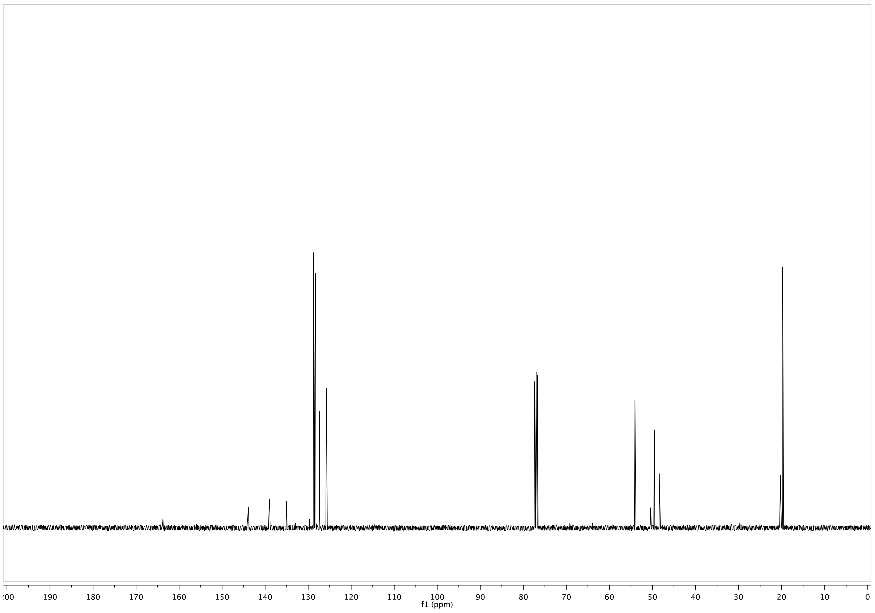
190
180
170
160
150
140
130
120
110
100
90
80
70
60
50
40
30
20
10
0
f1 (ppm)

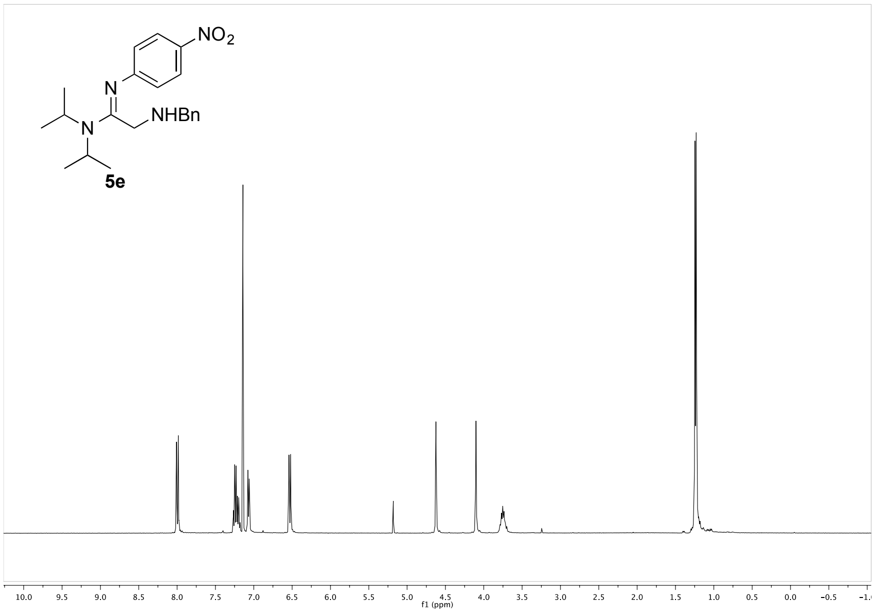
NO2
N
N
NHBn
5e
f1 (ppm)

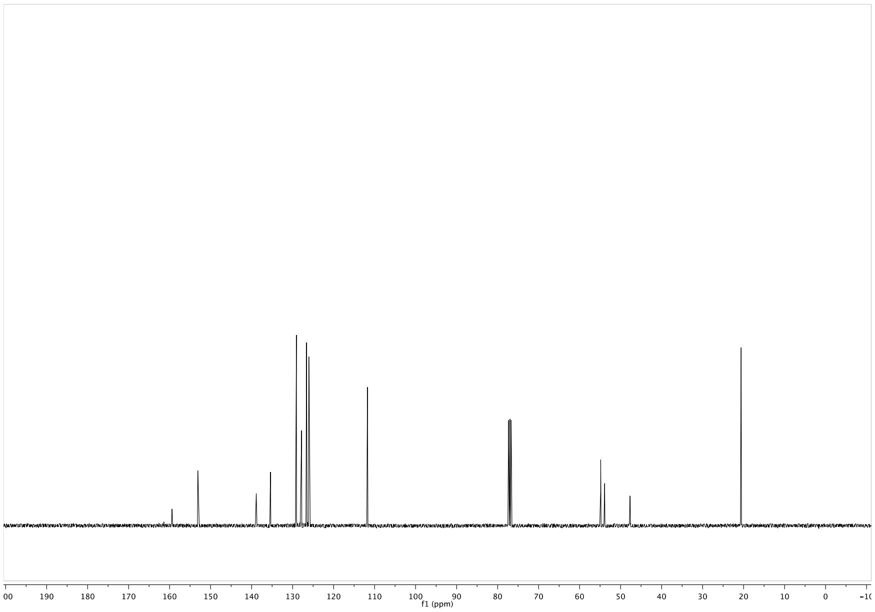
f1 (ppm)

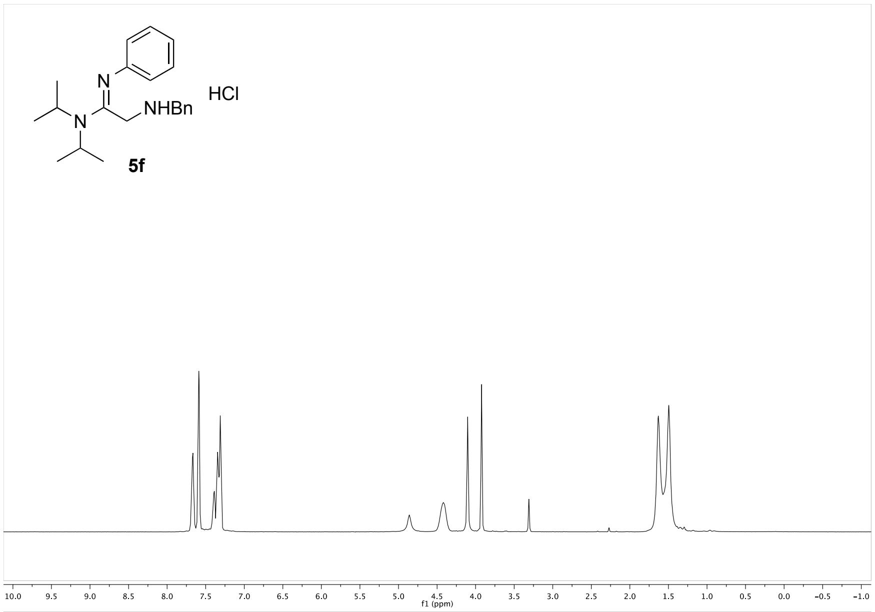
N
N
NHBn
HCl
5f
f1 (ppm)

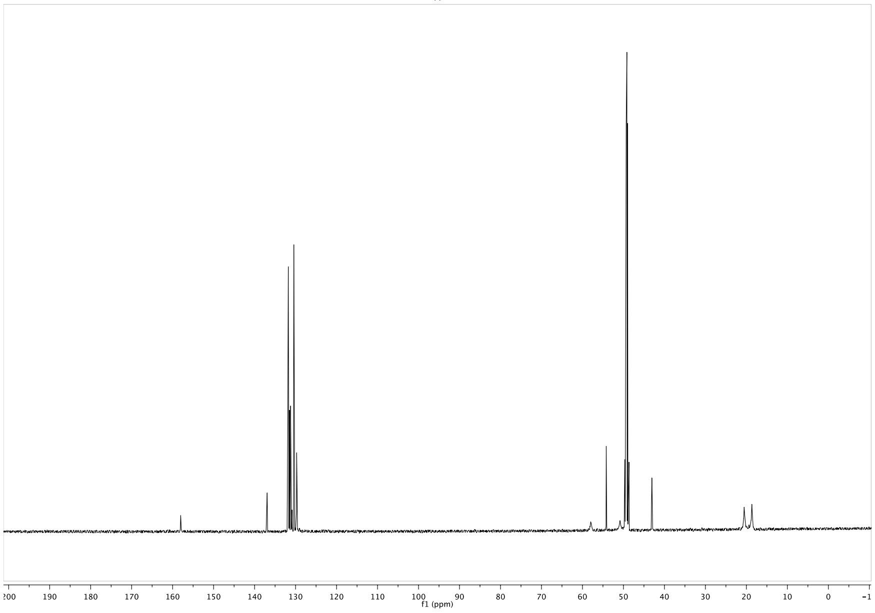
f1 (ppm)

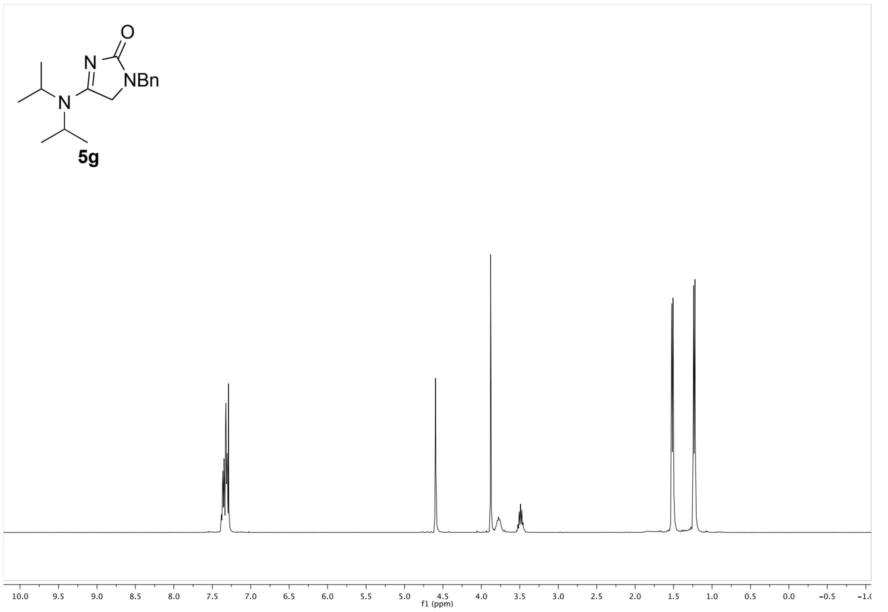
O
N
NBn
N
5g
10.0 9.5 9.0 8.5 8.0 7.5 7.0 6.5 6.0 5.5 5.0 4.5 4.0 3.5 3.0 2.5 2.0 1.5 1.0 0.5 0.0 -0.5
f1 (ppm)

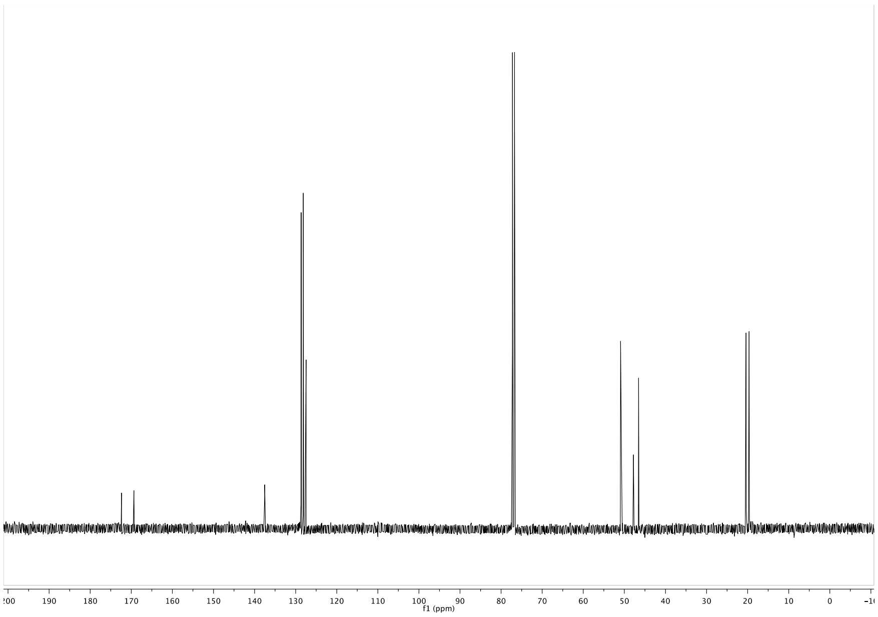
190 180 170 160 150 140 130 120 110 100 90 80 70 60 50 40 30 20 10 0
f1 (ppm)

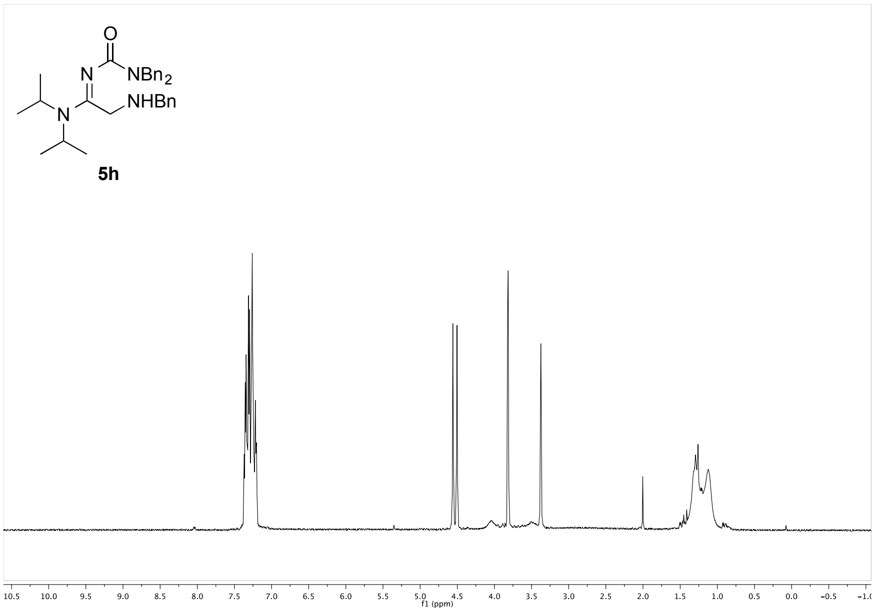
NBn2
NHBn
5h
f1 (ppm)

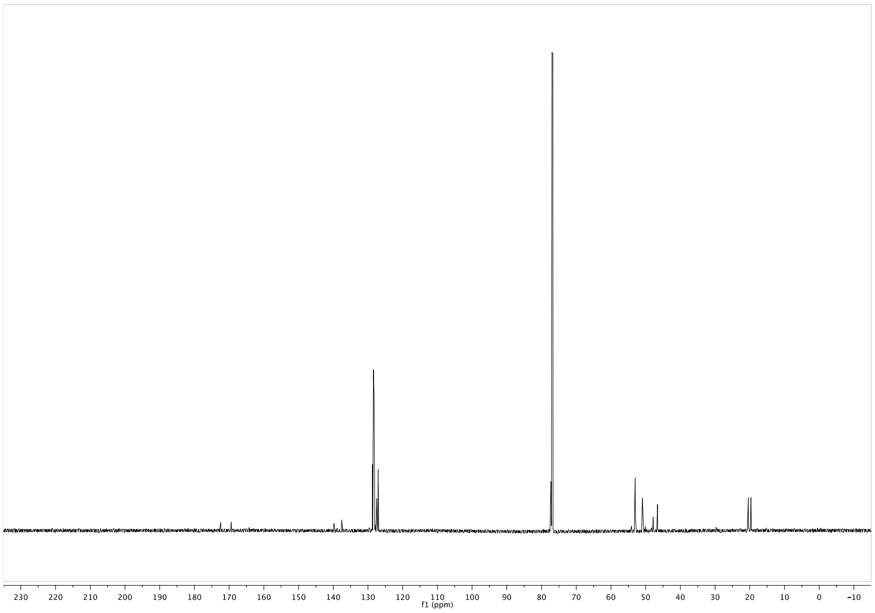
f1 (ppm)

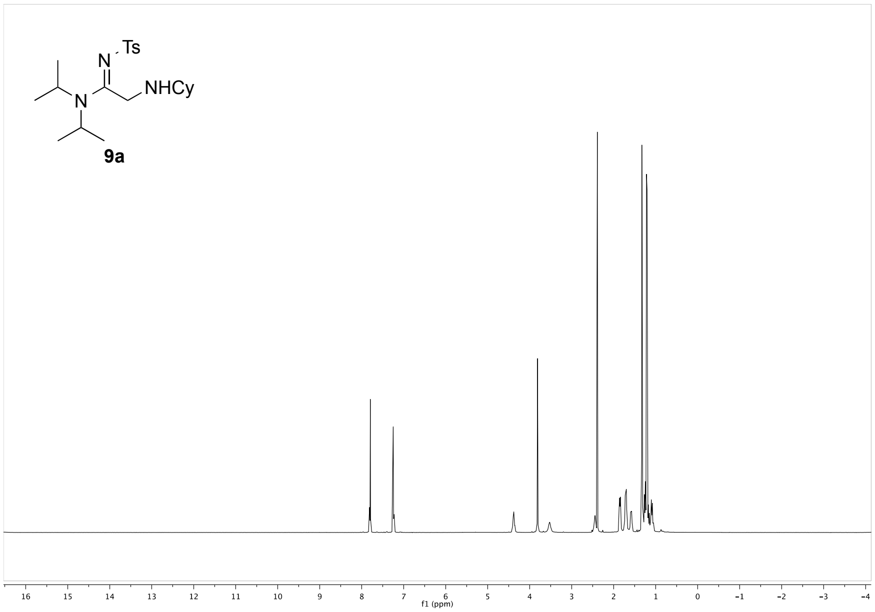
N
Ts
N
NHCy
9a
16 15 14 13 12 11 10 9 8 7 6 5 4 3 2 1 0 −1 −2 −3 −4
f1 (ppm)

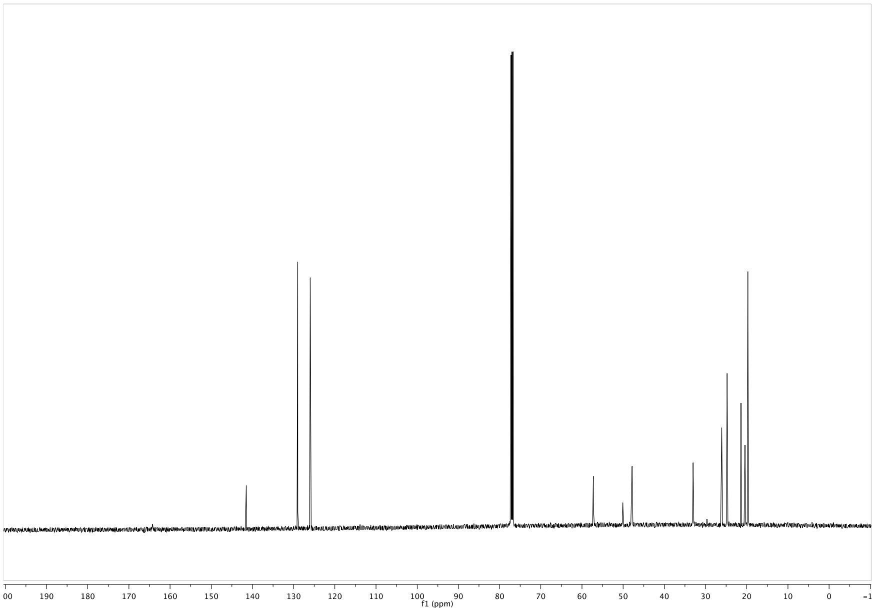
190 180 170 160 150 140 130 120 110 100 90 80 70 60 50 40 30 20 10 0
f1 (ppm)

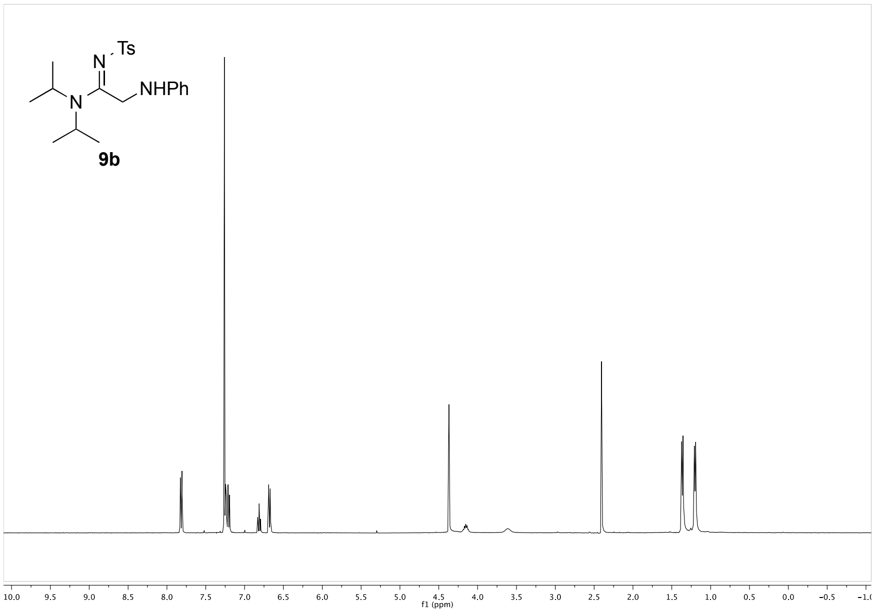
N
Ts
N
NHPh
9b
f1 (ppm)

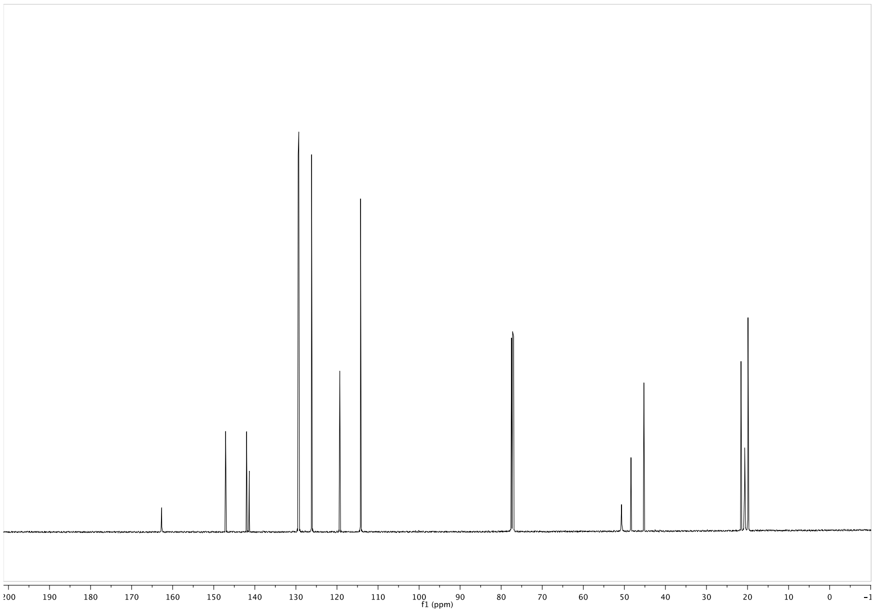
f1 (ppm)

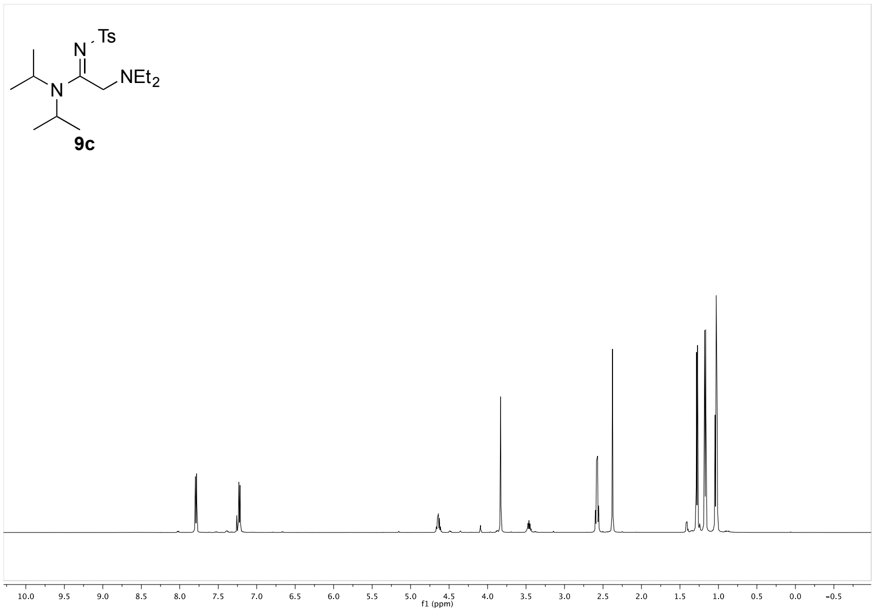
Ts
N
NEt2
N
9c
10.0 9.5 9.0 8.5 8.0 7.5 7.0 6.5 6.0 5.5 5.0 4.5 4.0 3.5 3.0 2.5 2.0 1.5 1.0 0.5 0.0 -0.5
f1 (ppm)

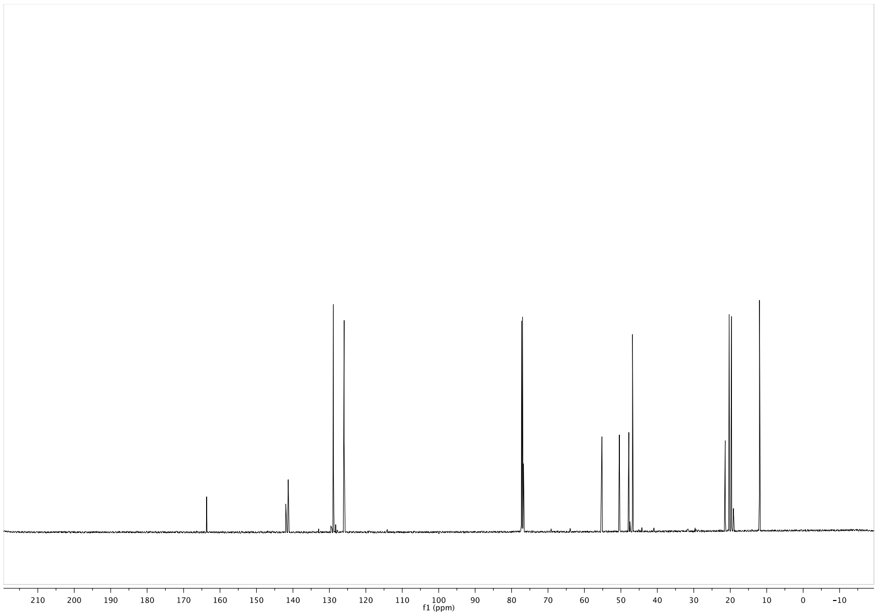
210 200 190 180 170 160 150 140 130 120 110 100 90 80 70 60 50 40 30 20 10 0 -10
f1 (ppm)

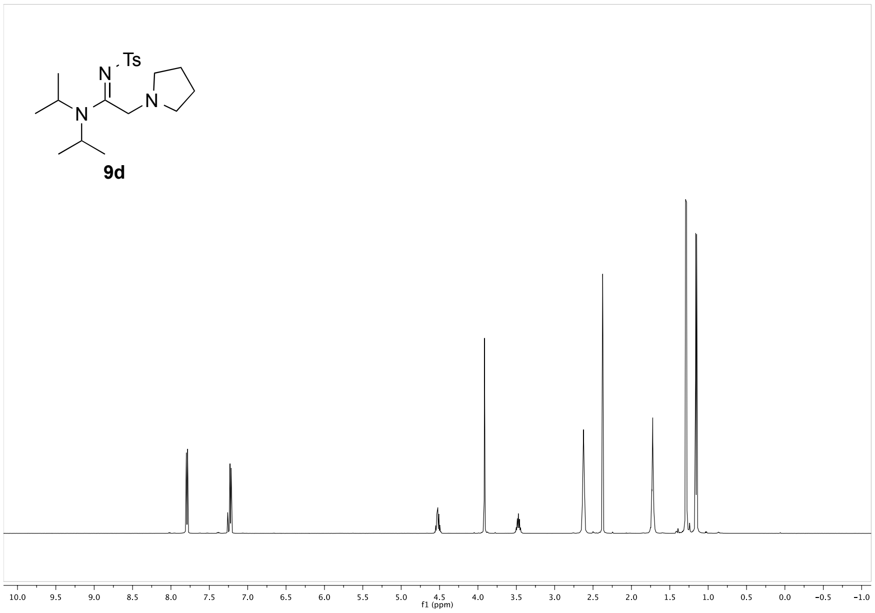
N
Ts
N
N
9d
10.0 9.5 9.0 8.5 8.0 7.5 7.0 6.5 6.0 5.5 5.0 4.5 4.0 3.5 3.0 2.5 2.0 1.5 1.0 0.5 0.0 −0.5 −1.0
f1 (ppm)

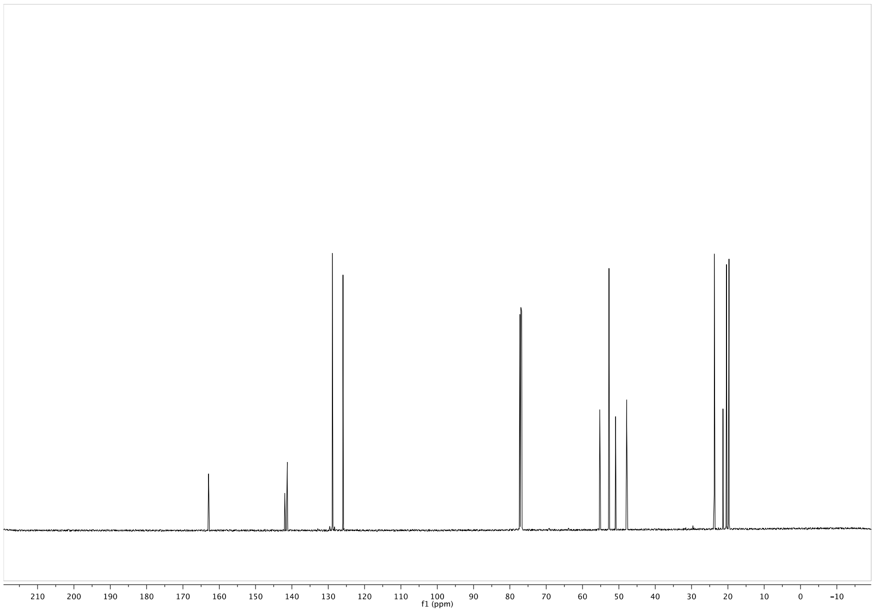
210 200 190 180 170 160 150 140 130 120 110 100 90 80 70 60 50 40 30 20 10 0 −10
f1 (ppm)

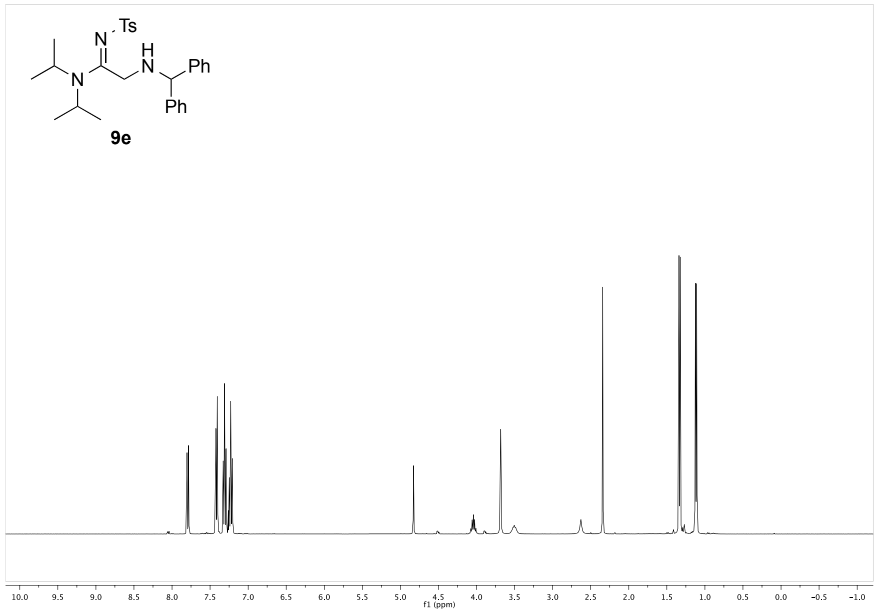
Ts
N
H
N
Ph
N
Ph
9e
f1 (ppm)

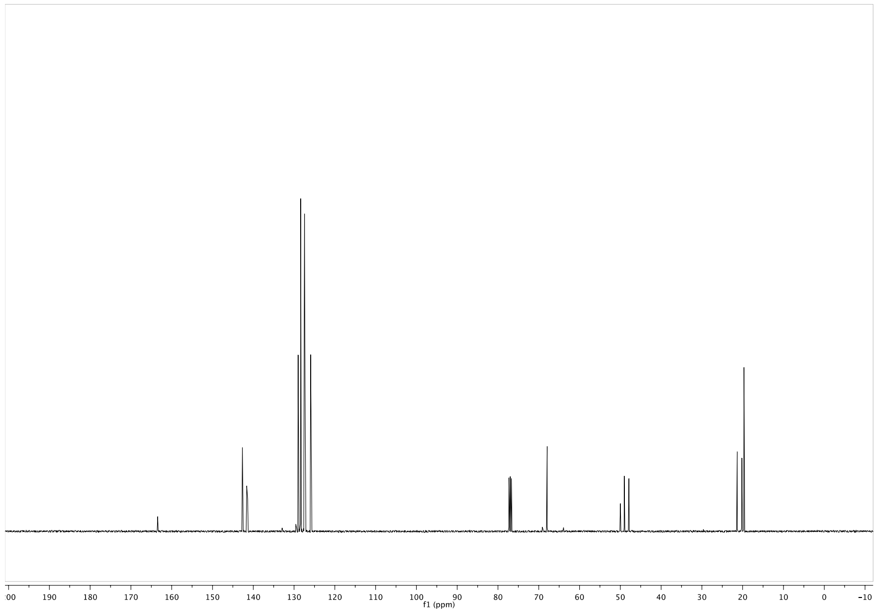
f1 (ppm)

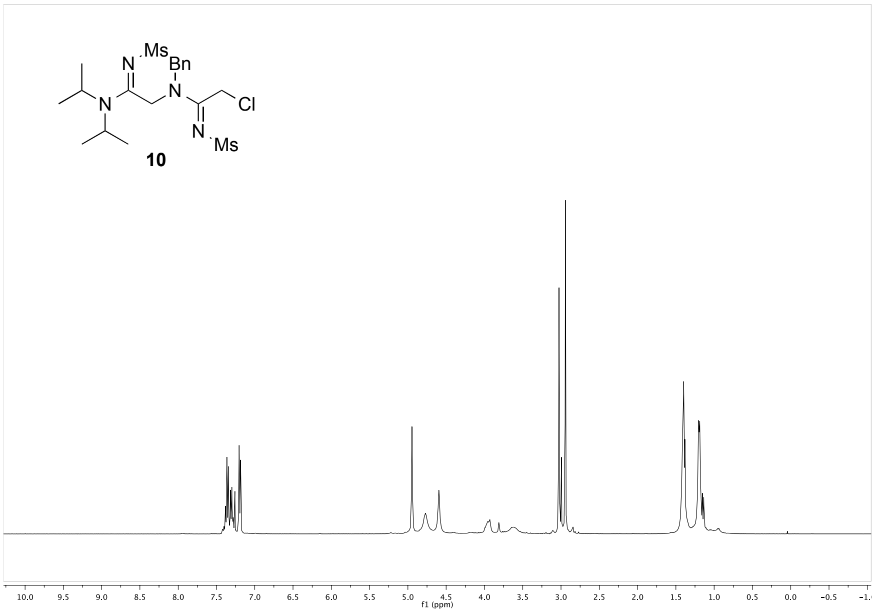
10
f1 (ppm)

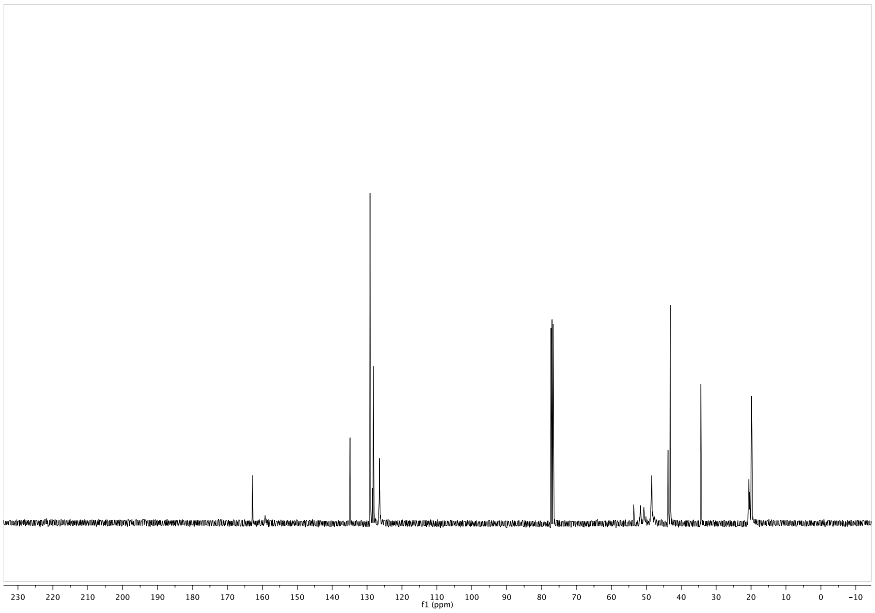
f1 (ppm)

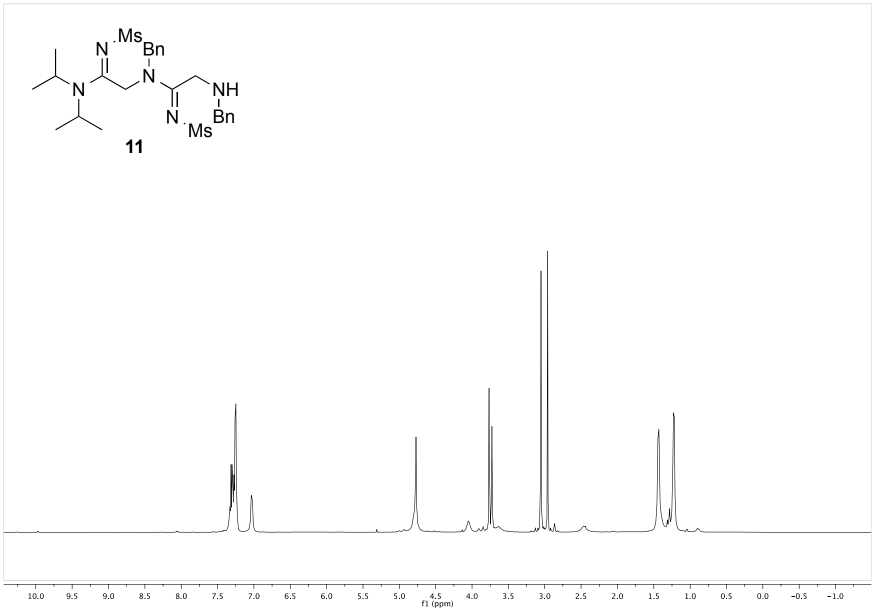
Ms
Bn
N
N
N
NH
Bn
N
Ms
11
f1 (ppm)

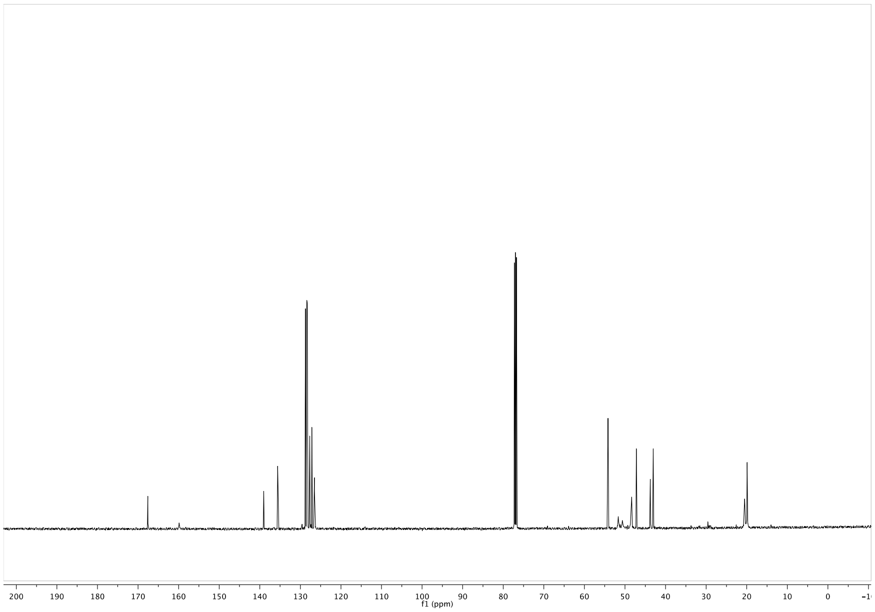
f1 (ppm)

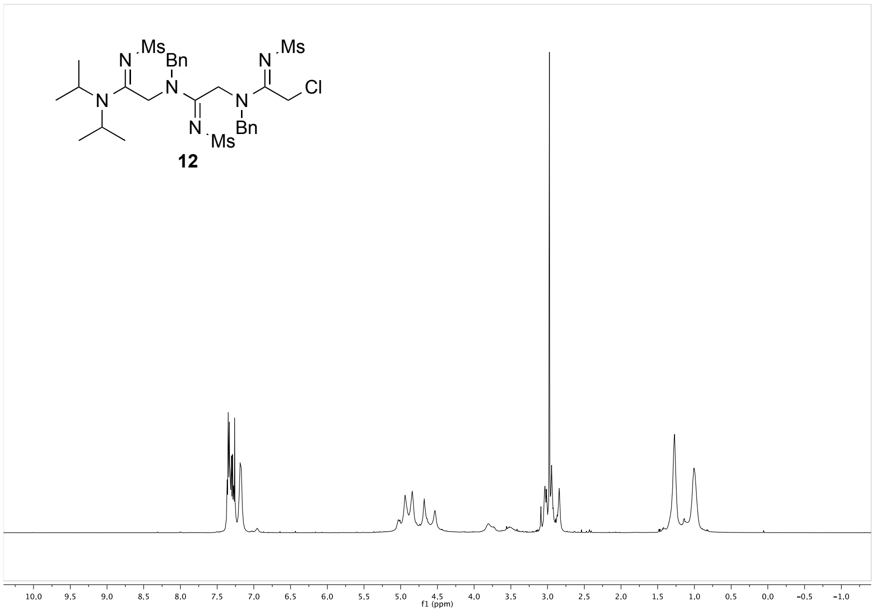
Ms
Bn
Cl
12
f1 (ppm)

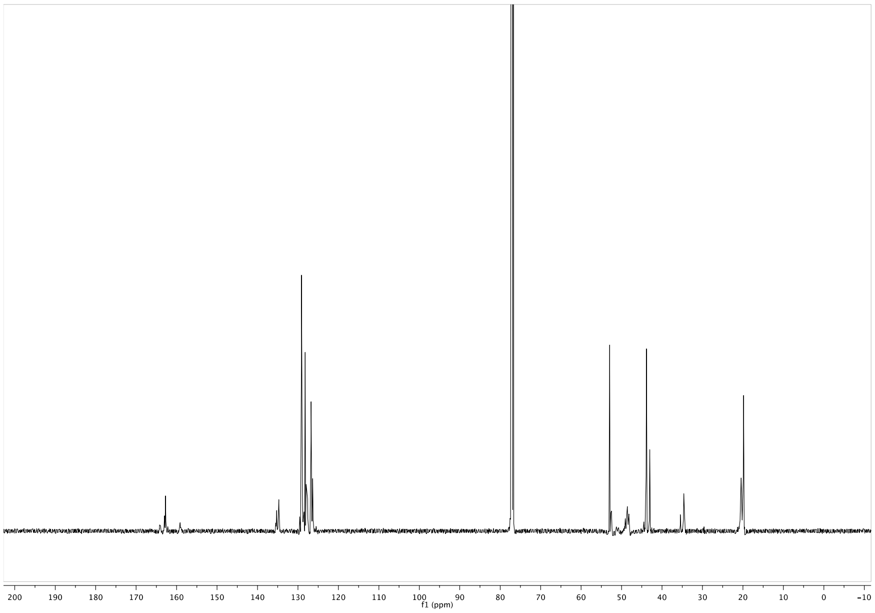
f1 (ppm)

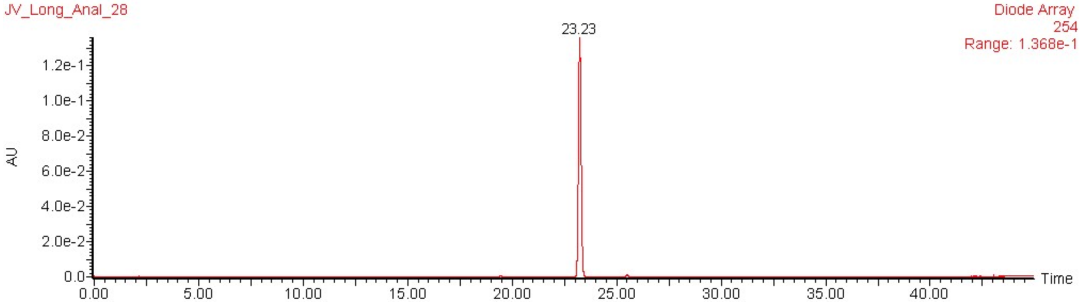
JV_Long_Anal_28
Diode Array
254
Range: 1.368e-1
23.23
AU
Time

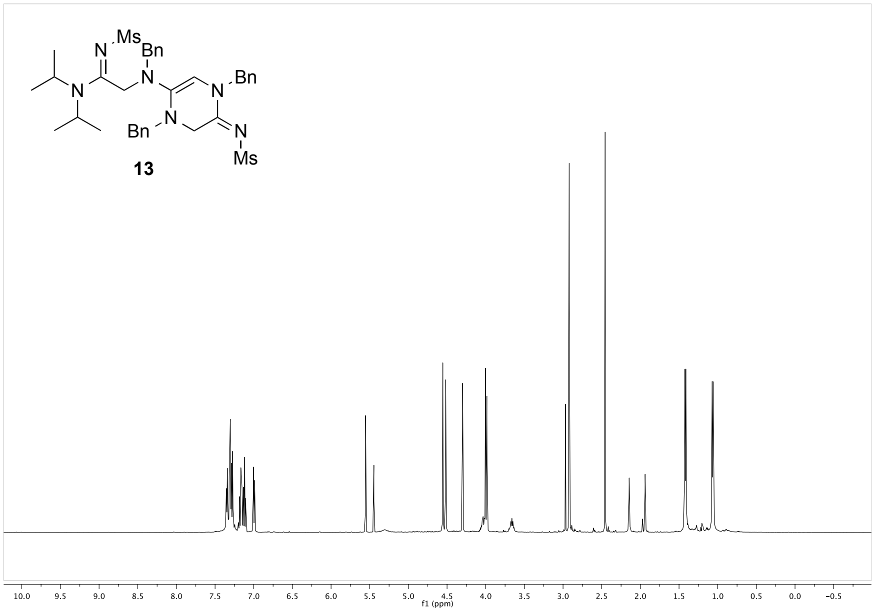
N
Ms
Bn
N
N
Bn
N
Bn
N
N
Ms
13
10.0 9.5 9.0 8.5 8.0 7.5 7.0 6.5 6.0 5.5 5.0 4.5 4.0 3.5 3.0 2.5 2.0 1.5 1.0 0.5 0.0 -0.5
f1 (ppm)

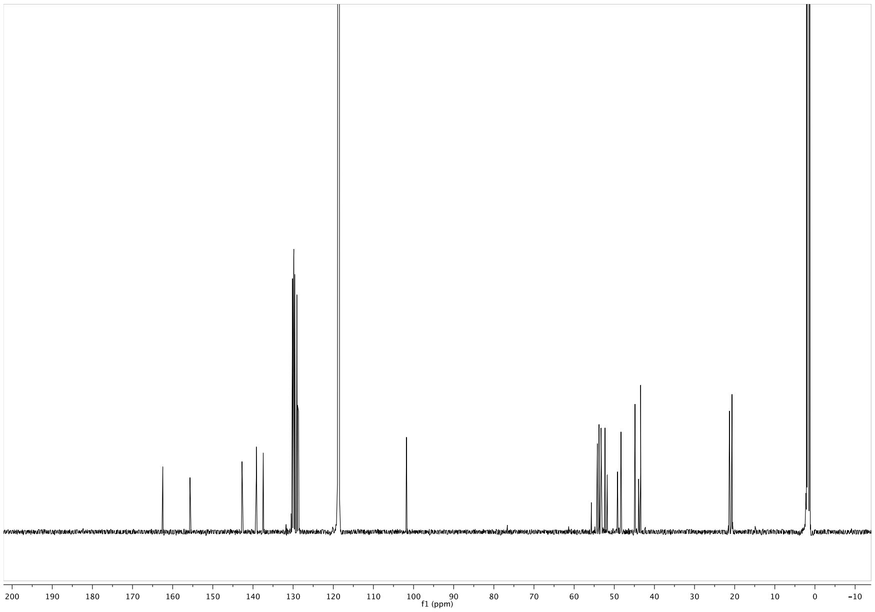
200 190 180 170 160 150 140 130 120 110 100 90 80 70 60 50 40 30 20 10 0 -10
f1 (ppm)

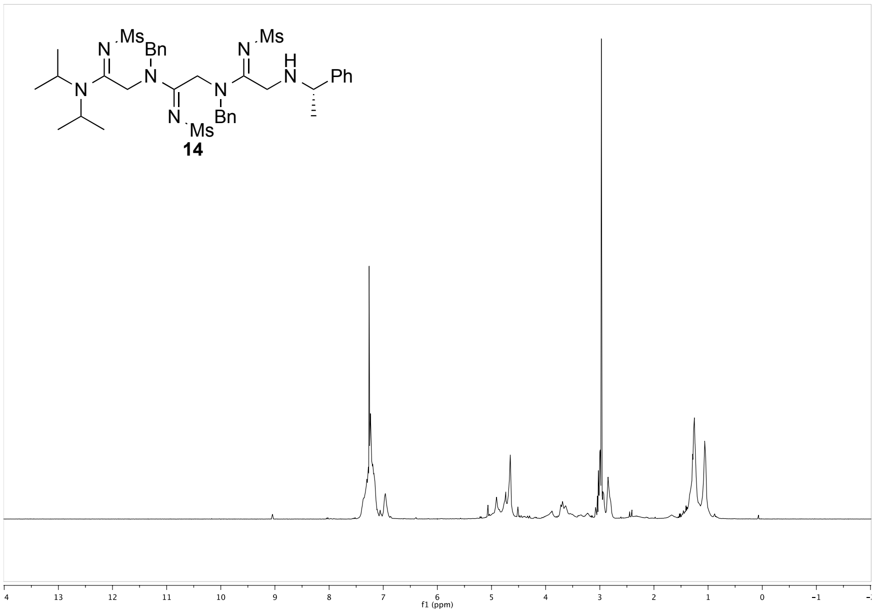
14
f1 (ppm)

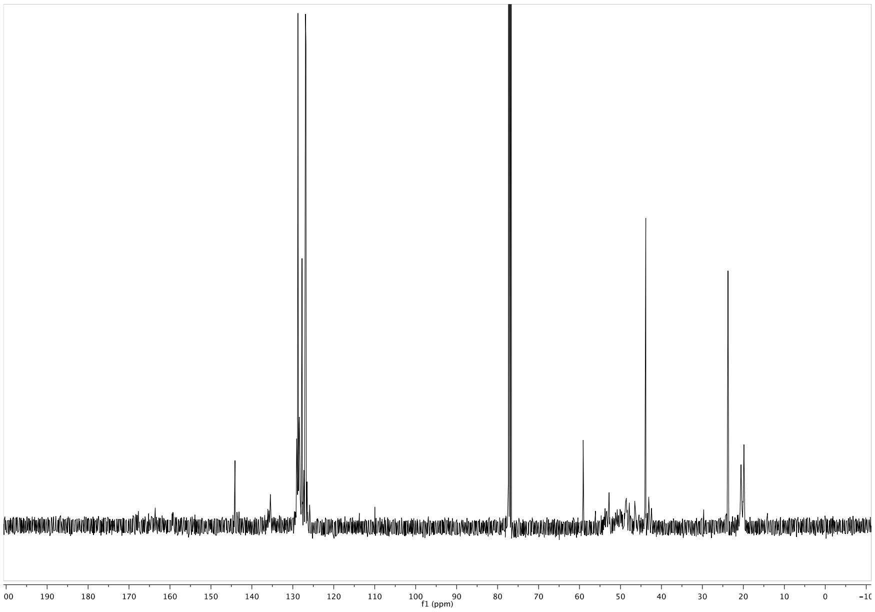
f1 (ppm)

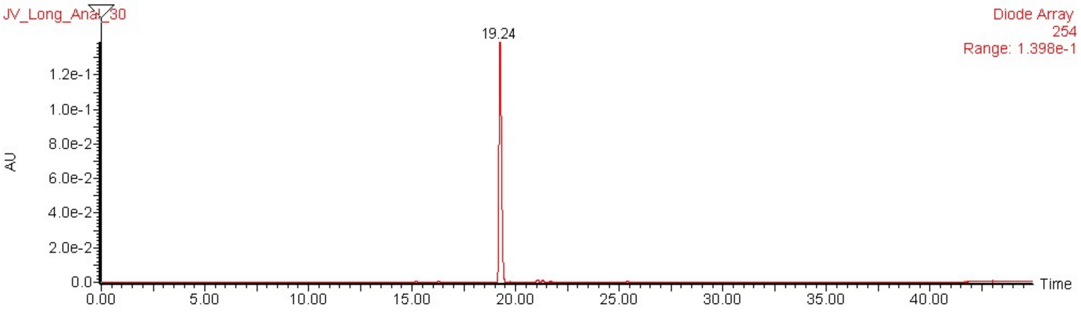
JV_Long_Anal_30
19.24
Diode Array
254
Range: 1.398e-1
AU
Time

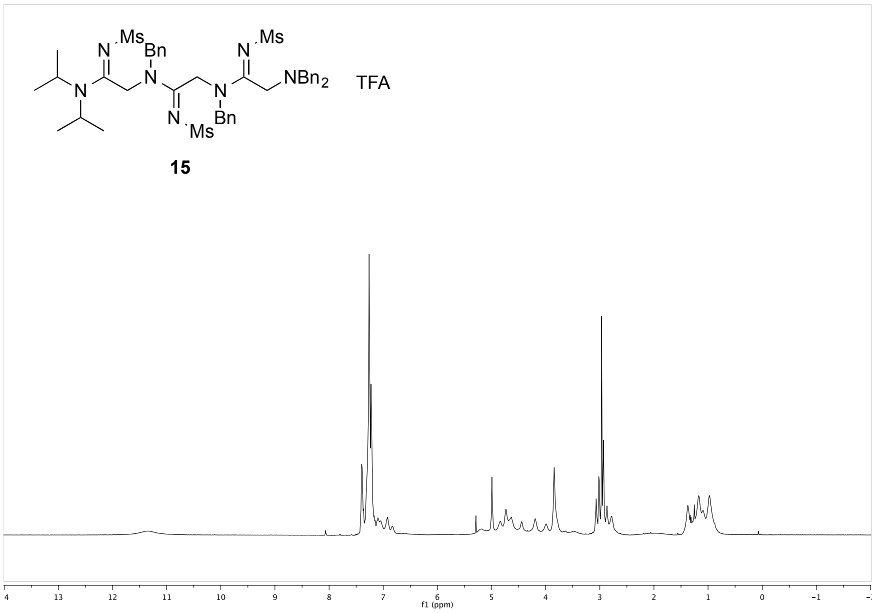
Ms
Bn
Ms
NBn2
TFA
Ms
Bn
15
f1 (ppm)

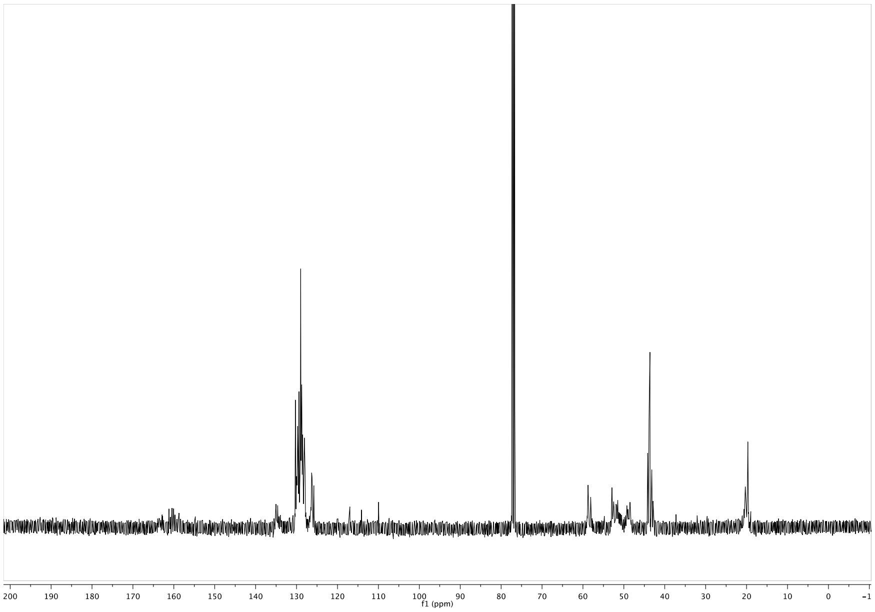
f1 (ppm)

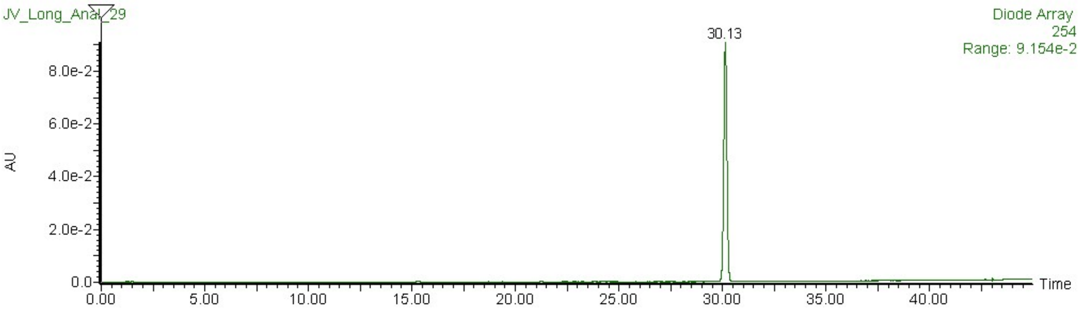
JV_Long_Anal_29
Diode Array
254
Range: 9.154e-2
30.13
AU
Time

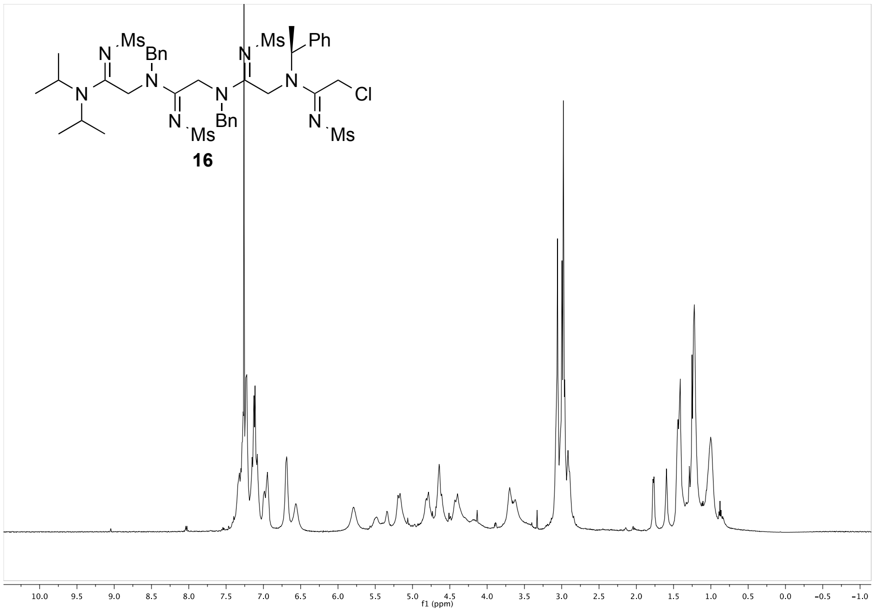
Ms
Bn
N
Ms
Ph
Cl
Ms
Bn
Ms
16
f1 (ppm)

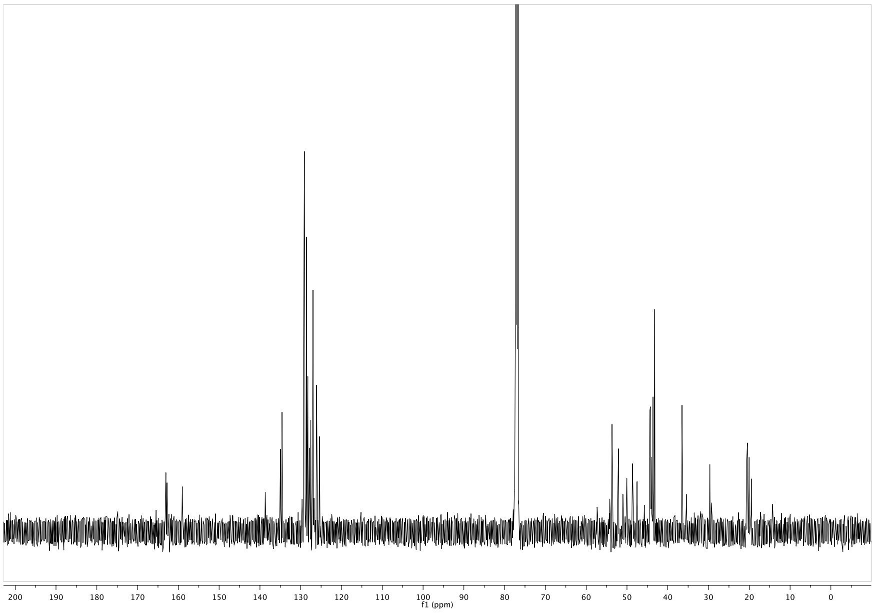
f1 (ppm)

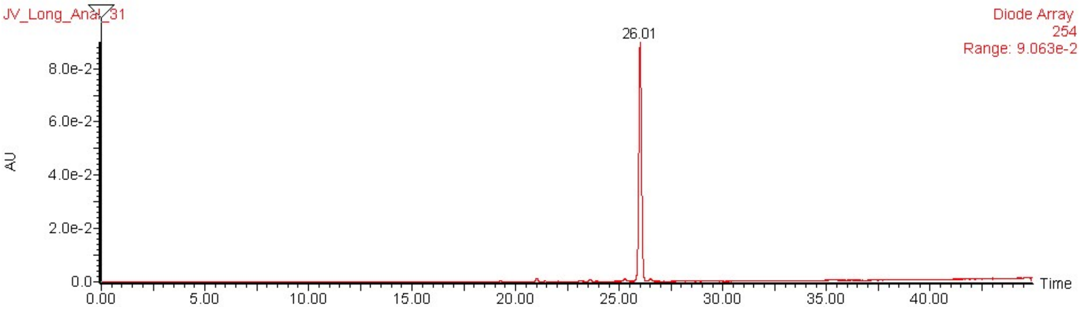
JV_Long_Anal_31
26.01
Diode Array
254
Range: 9.063e-2
AU
Time

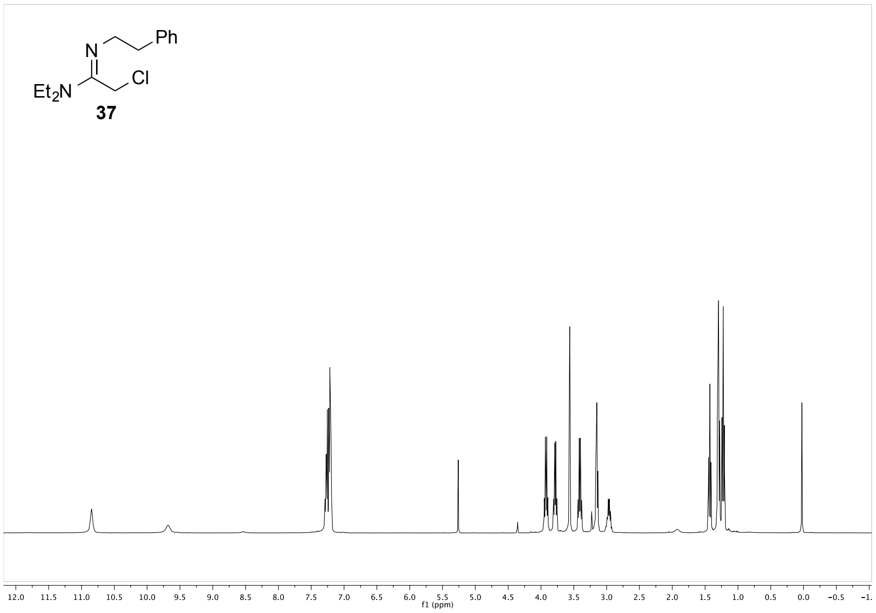
Ph
N
Cl
Et2N
37
f1 (ppm)

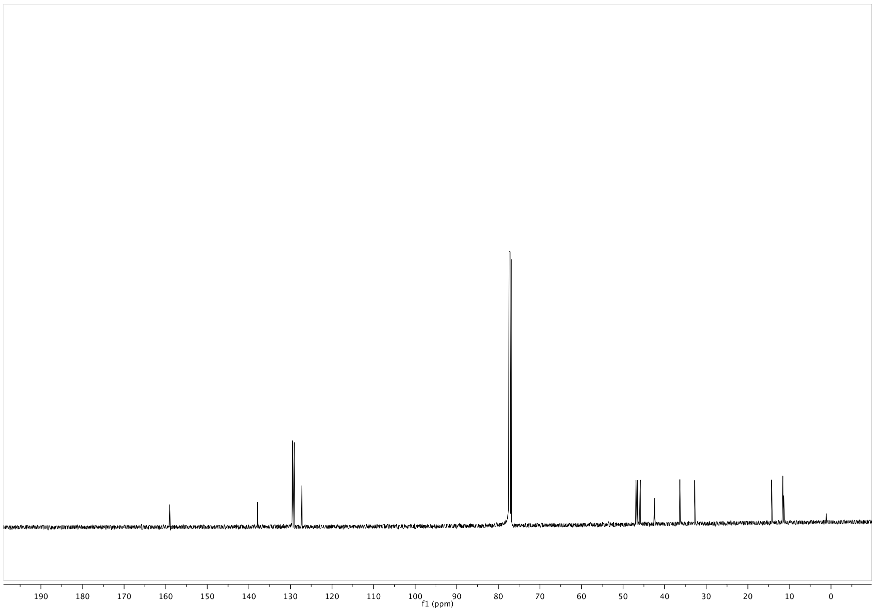
f1 (ppm)

Solid Phase

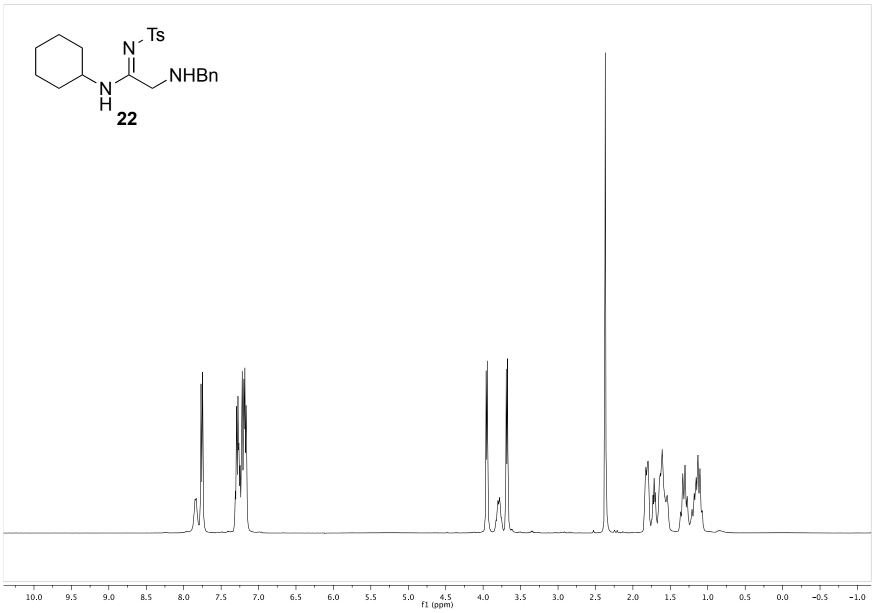

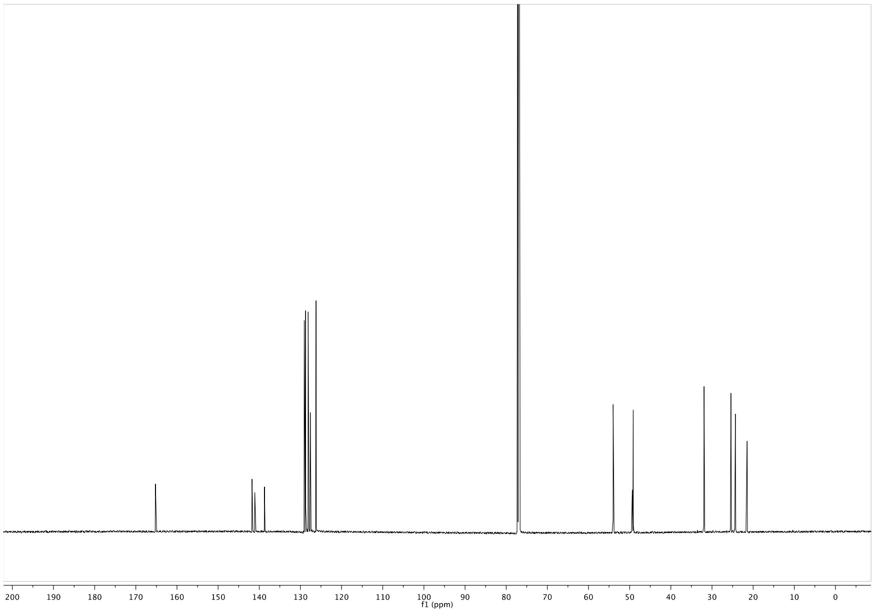

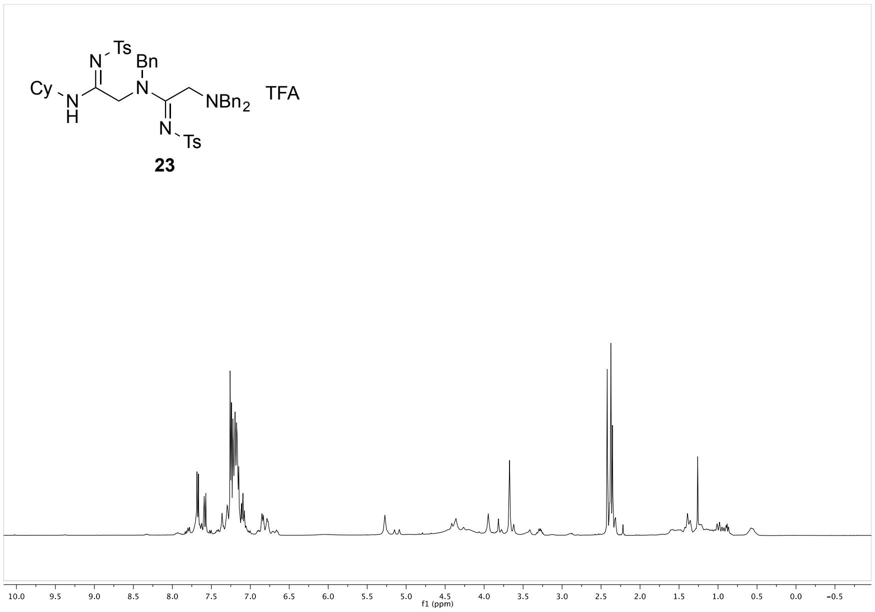
Ts
Bn
Cy
N
H
NBn2
TFA
23
10.0 9.5 9.0 8.5 8.0 7.5 7.0 6.5 6.0 5.5 5.0 4.5 4.0 3.5 3.0 2.5 2.0 1.5 1.0 0.5 0.0 -0.5
f1 (ppm)

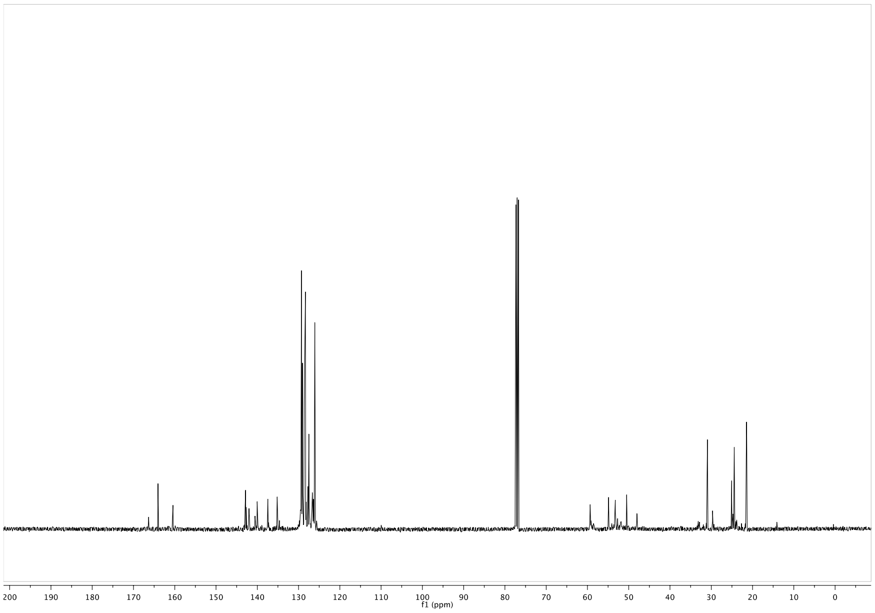
200 190 180 170 160 150 140 130 120 110 100 90 80 70 60 50 40 30 20 10 0
f1 (ppm)

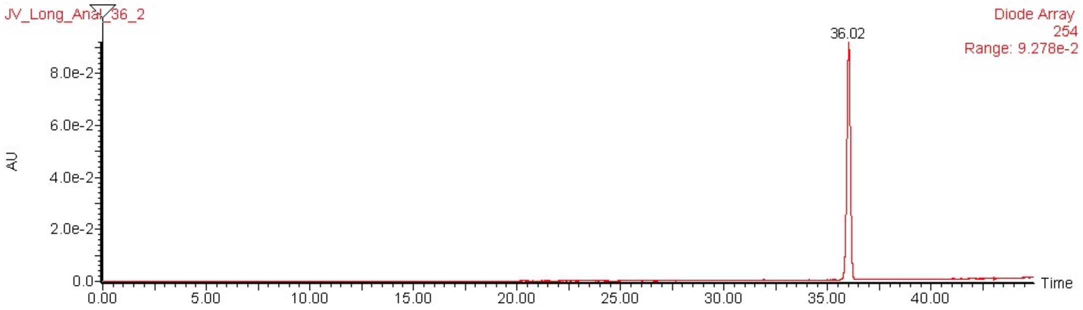
JV_Long_Anal_36_2
Diode Array
254
Range: 9.278e-2
36.02
AU
8.0e-2
6.0e-2
4.0e-2
2.0e-2
0.0
0.00 5.00 10.00 15.00 20.00 25.00 30.00 35.00 40.00
Time

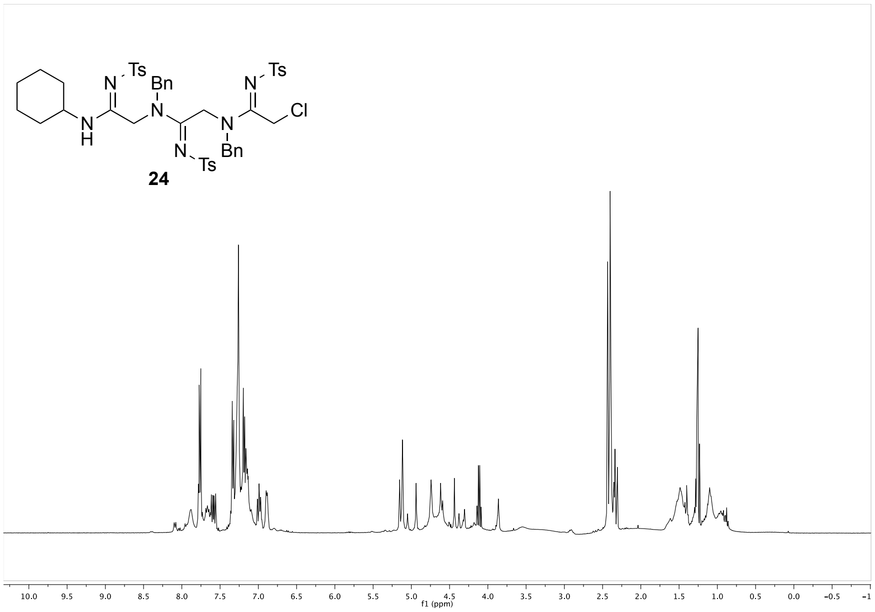
Ts
Bn
N
H
Cl
Ts
Bn
Ts
24
f1 (ppm)

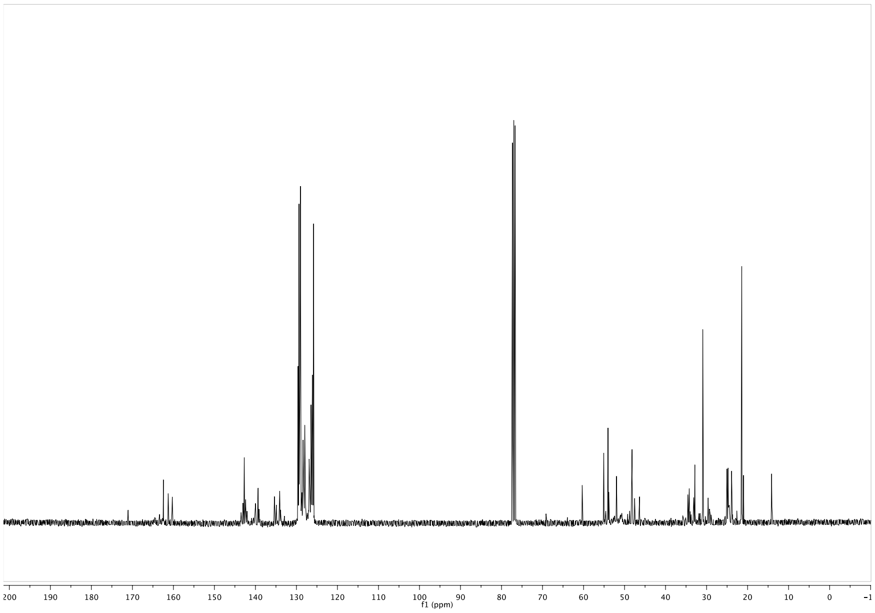
f1 (ppm)

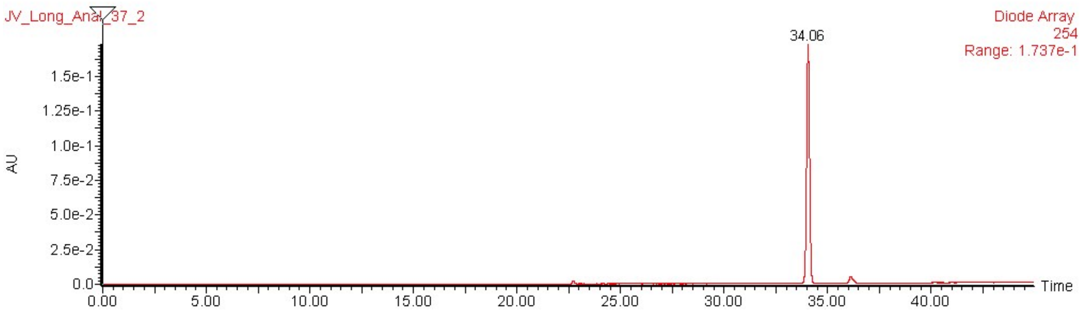
JV_Long_Anal_37_2
Diode Array
254
Range: 1.737e-1
34.06
AU
Time

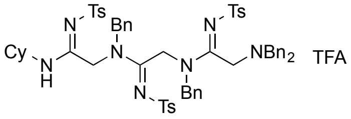
Ts
Bn
Cy
H
N
NBn2
TFA

25

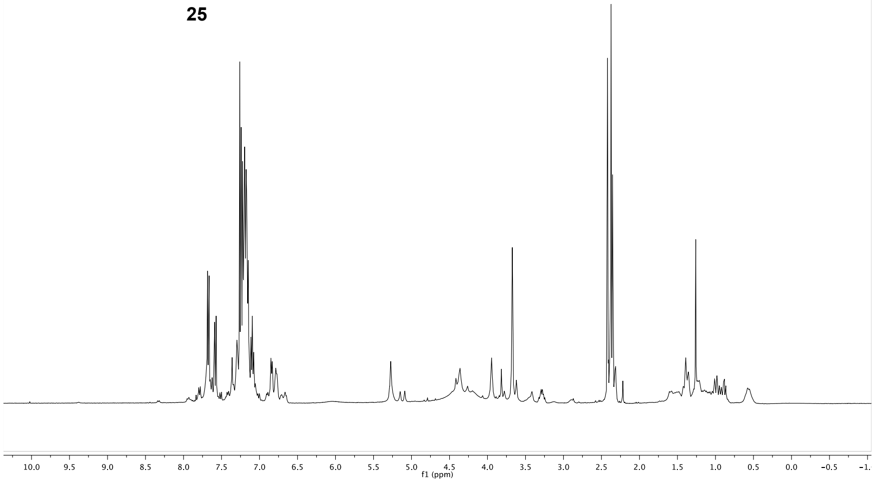
f1 (ppm)

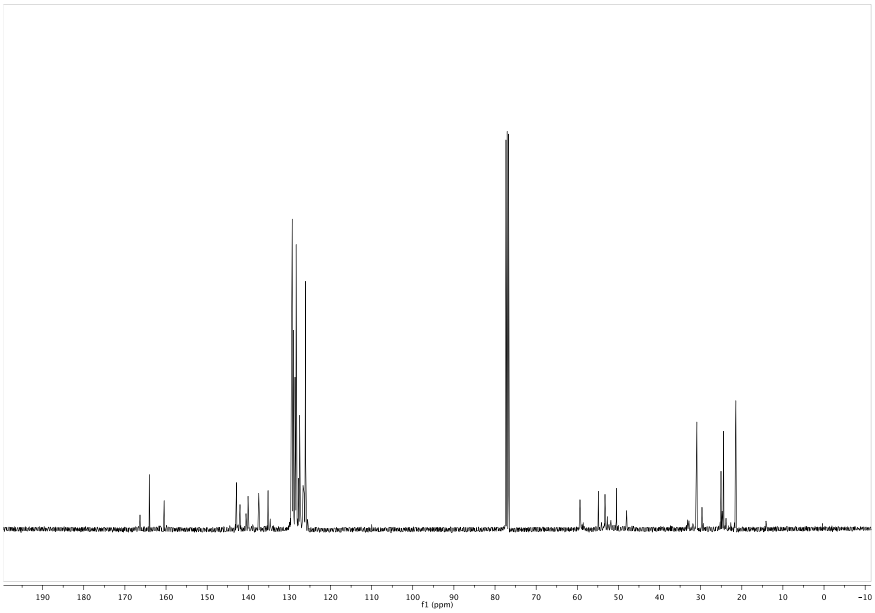
f1 (ppm)

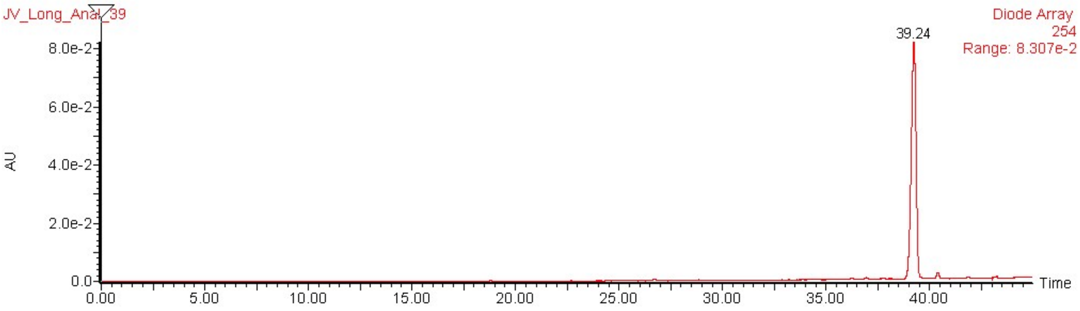
JV_Long_Anal_39
Diode Array
254
Range: 8.307e-2
39.24
AU
Time

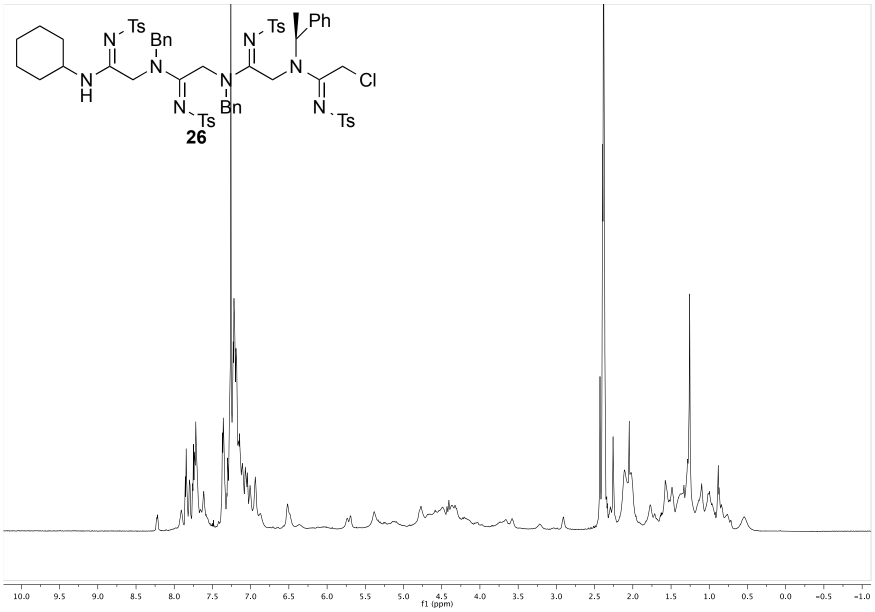
Ts
Bn
Ph
Cl
Ts
Bn
Ts
Ts
26
f1 (ppm)

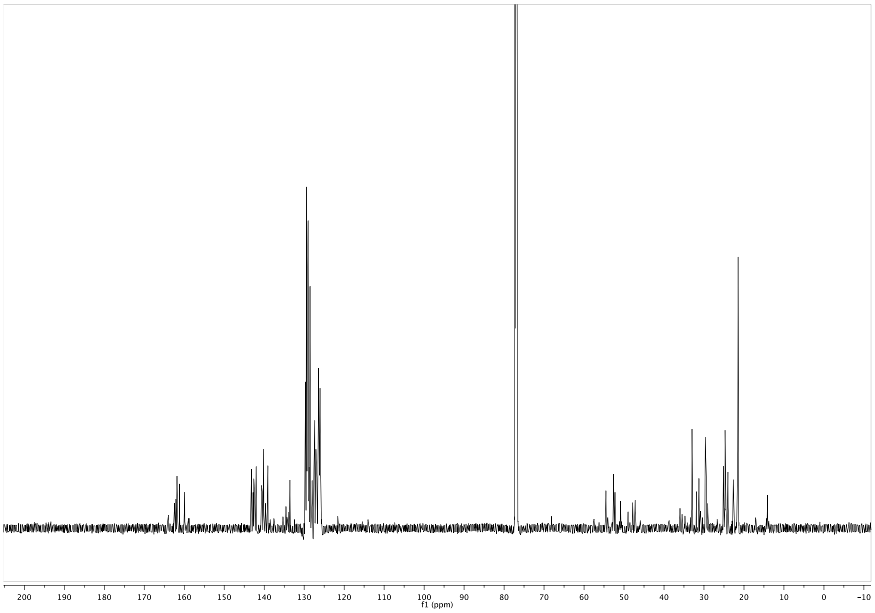
f1 (ppm)

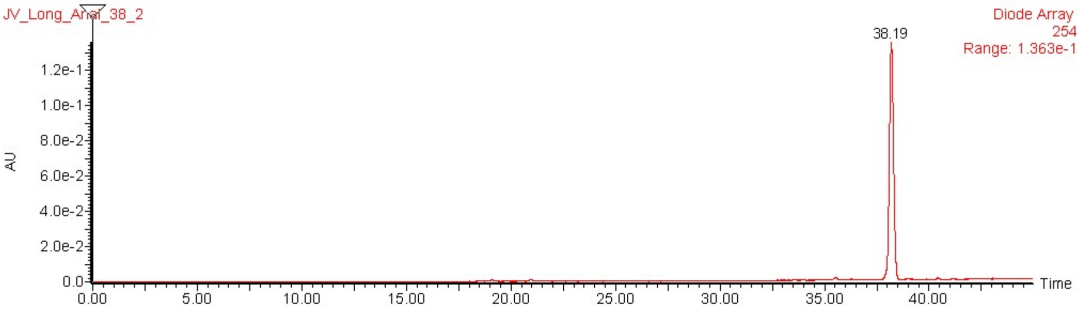
JV_Long_Ami_38_2
Diode Array
254
Range: 1.363e-1
38.19
AU
Time

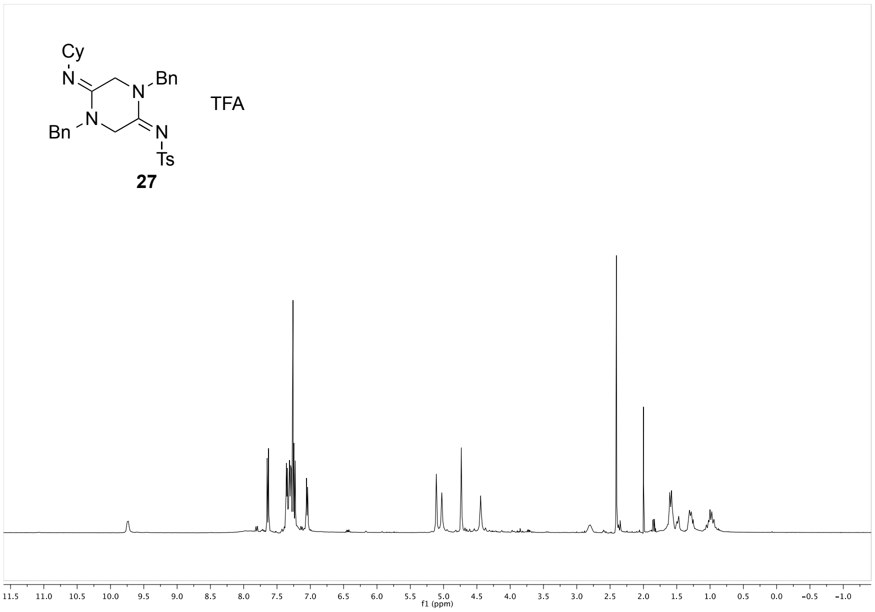
Cy
N
N
Bn
Bn
N
N
Ts
27
TFA
f1 (ppm)

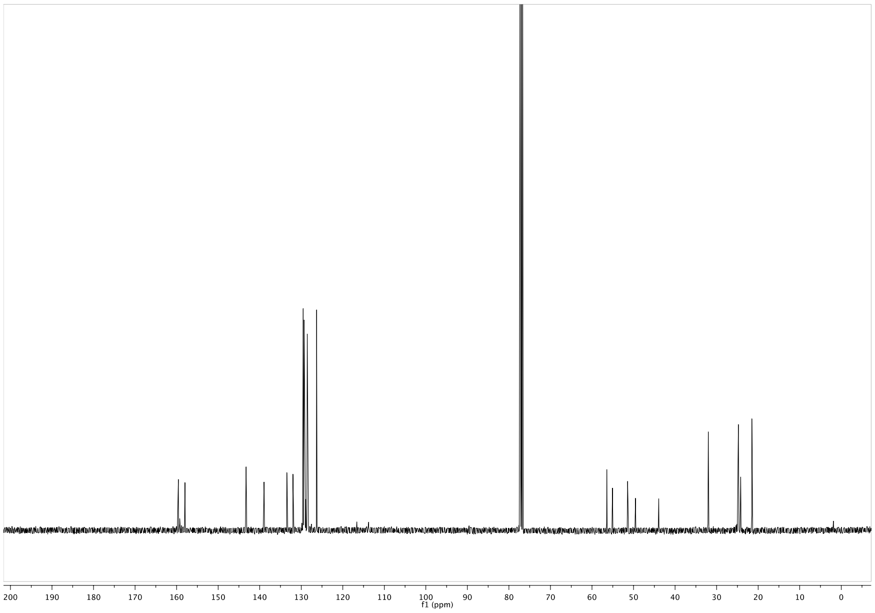
f1 (ppm)

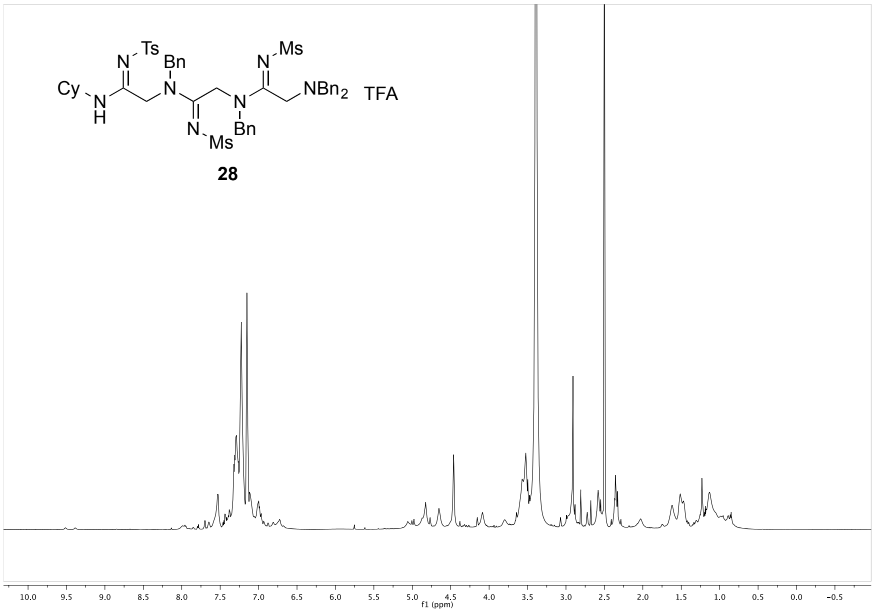
Ts
Bn
Ms
Cy
NBn2
TFA
Ms
Bn
28
f1 (ppm)

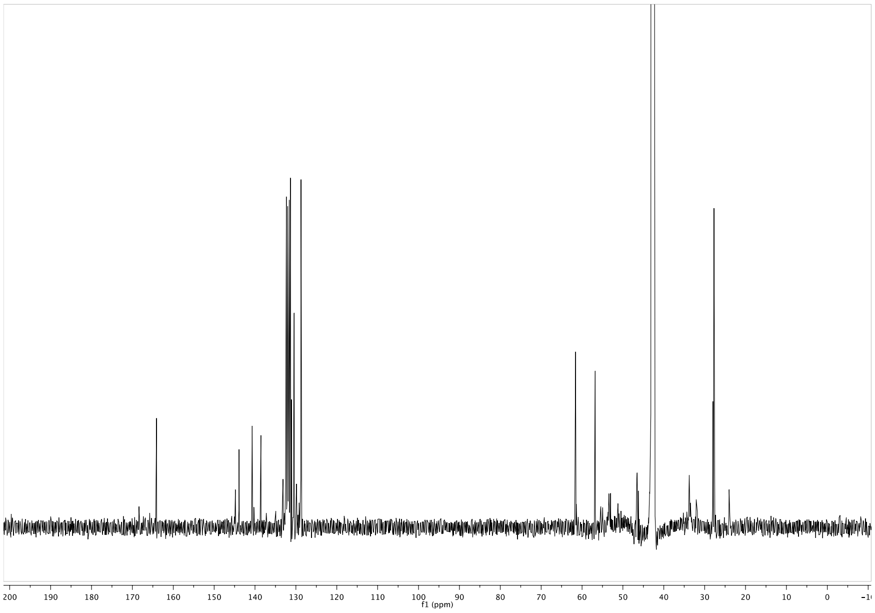
f1 (ppm)

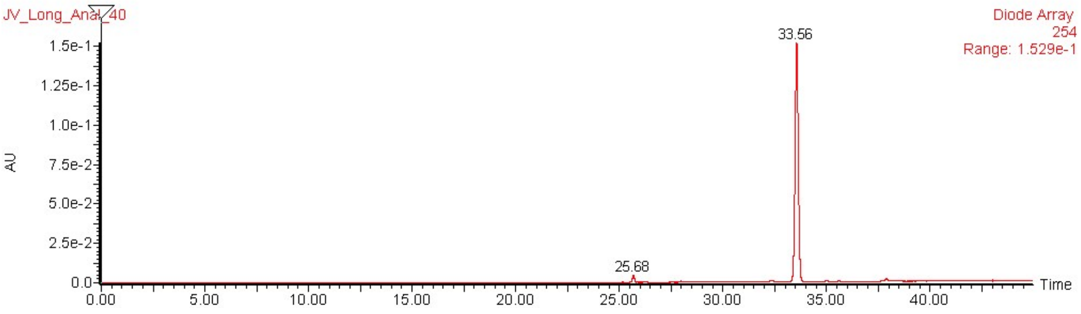
JV_Long_Anal_40
Diode Array
254
Range: 1.529e-1
33.56
25.68
AU
Time

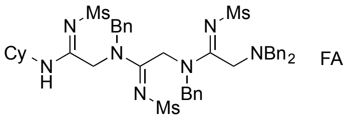
Ms
N
Bn
Cy
N
H
N
N
Ms
Bn
N
Ms
N
NBn2
FA

29

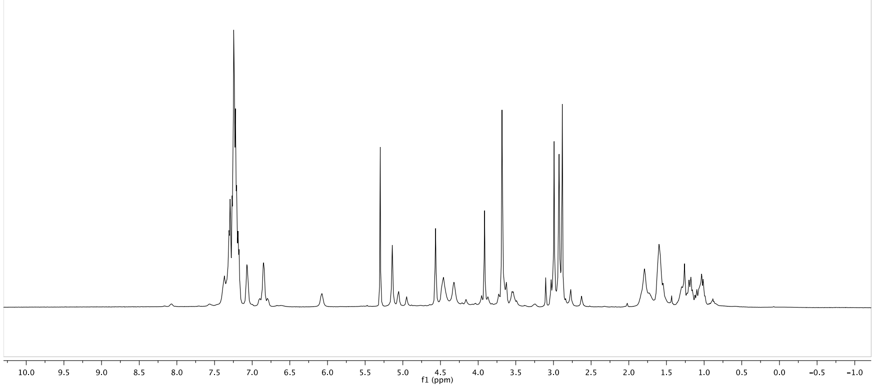
f1 (ppm)

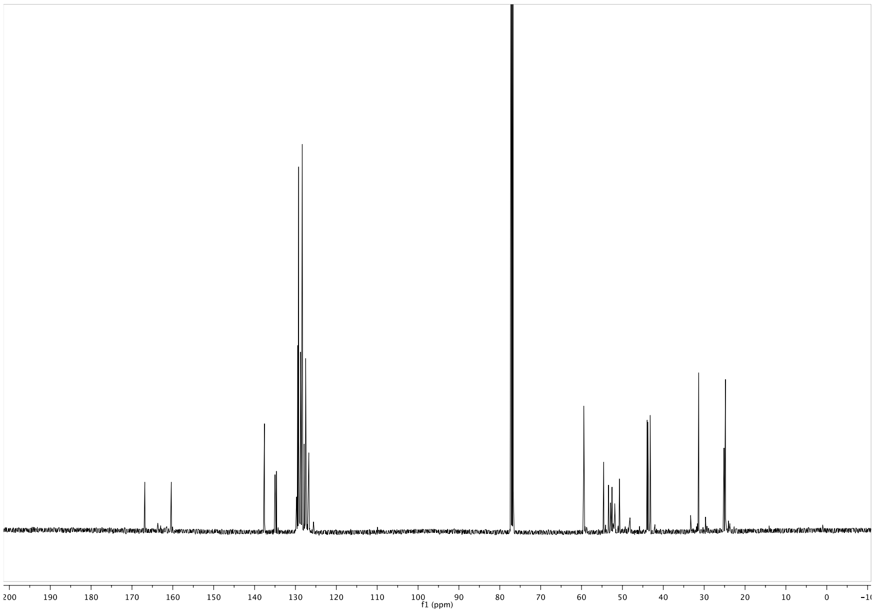
f1 (ppm)

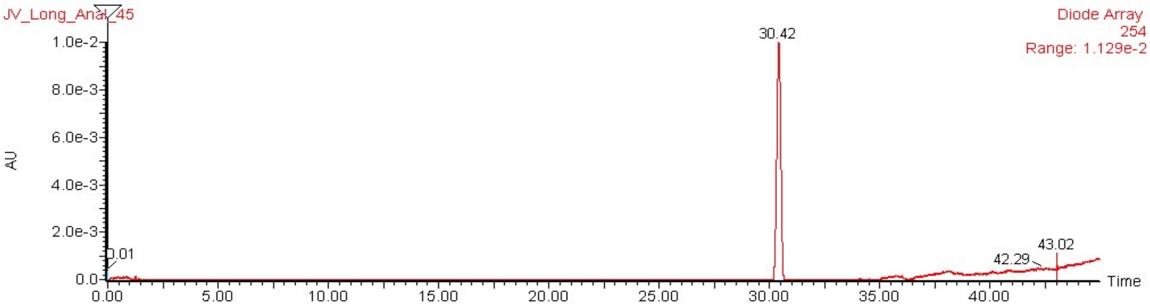
JV_Long_Anal_45
Diode Array
254
Range: 1.129e-2
30.42
0.01
42.29
43.02
AU
Time

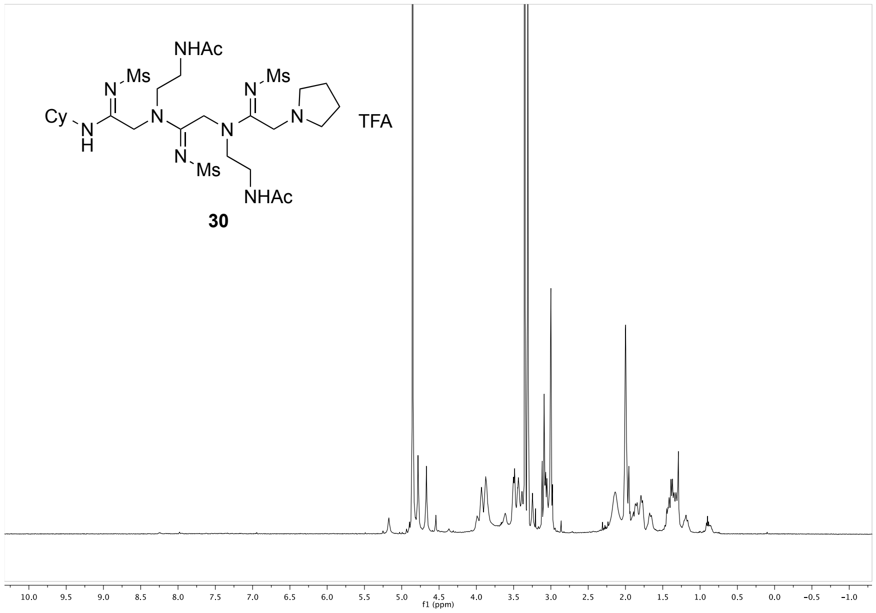
NHAc
Ms
Cy
N
H
TFA
NHAc
30
f1 (ppm)

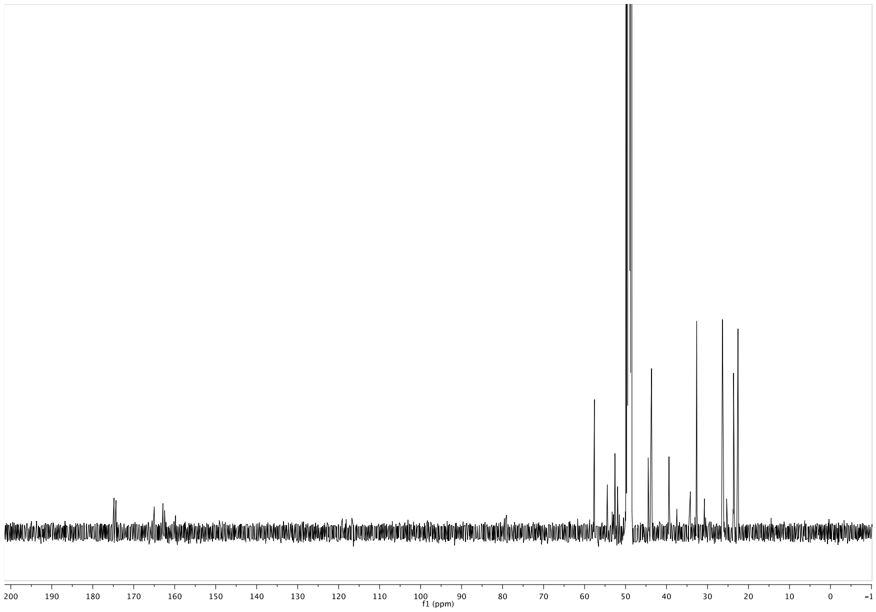
f1 (ppm)

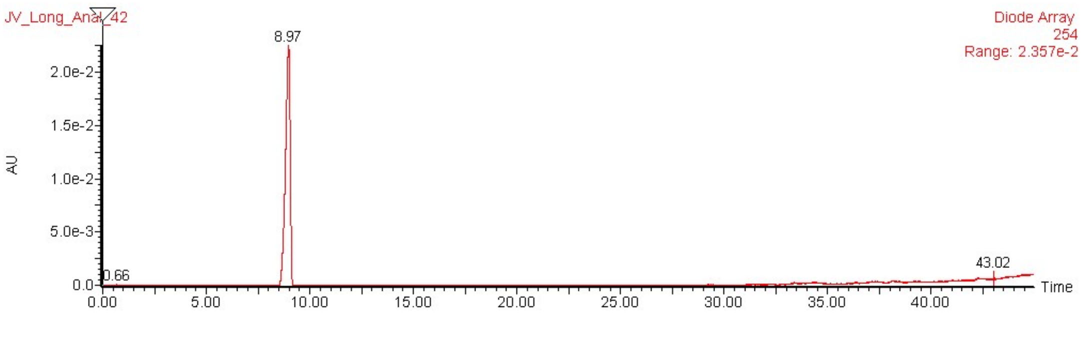
JV_Long_Anal_42
Diode Array
254
Range: 2.357e-2
8.97
0.66
43.02
AU
Time

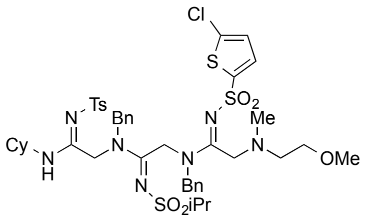
Cl
S
SO2
N
Me
N
OMe
Ts
Bn
Cy
N
H
Bn
SO2iPr

31

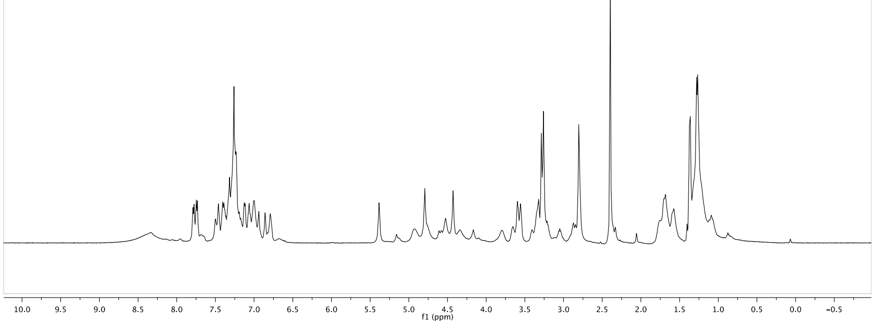
f1 (ppm)

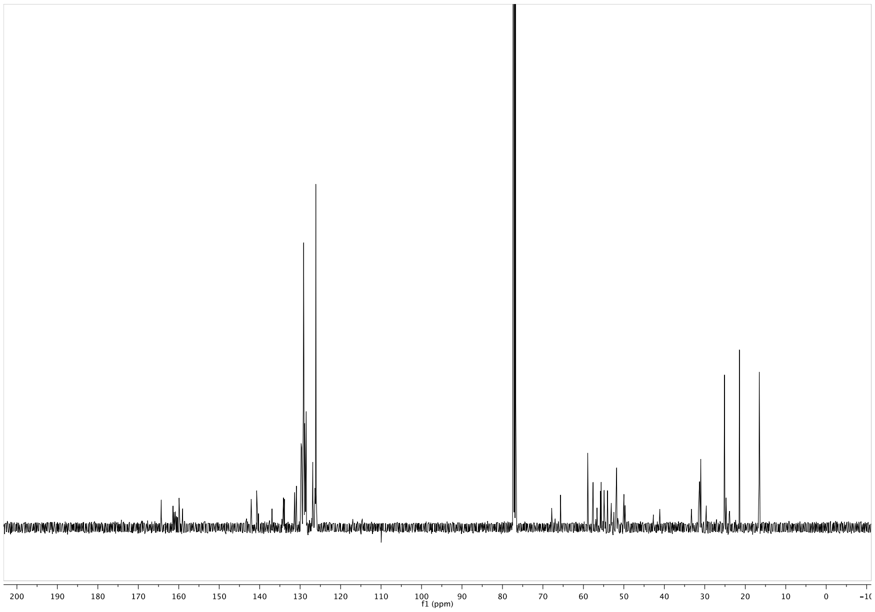
f1 (ppm)

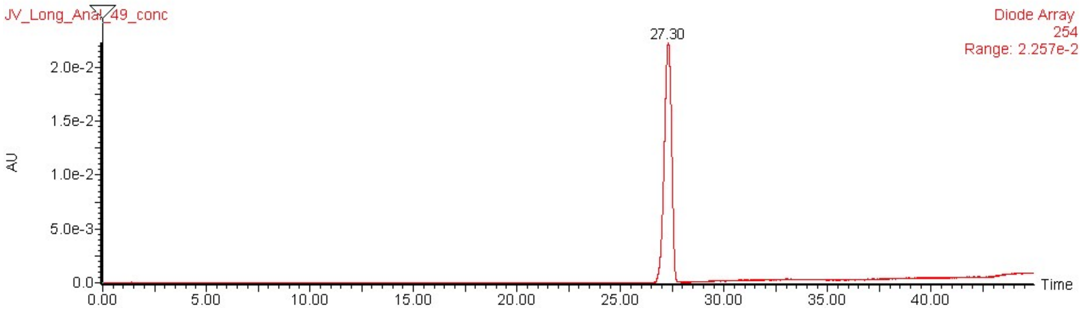
JV_Long_Anal_49_conc
27.30
Diode Array
254
Range: 2.257e-2
AU
Time

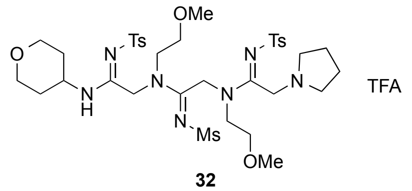

OMe
N Ts
O
N
H
N
N Ms
N Ts
N
N
OMe
TFA

32

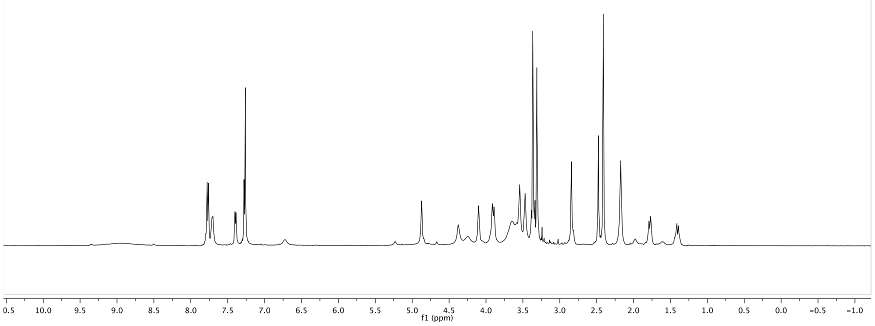

f1 (ppm)

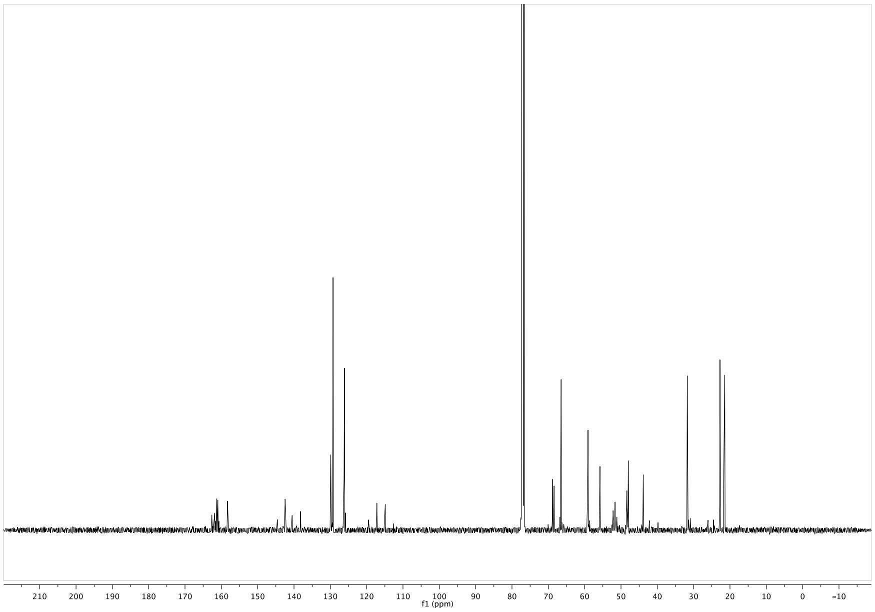

f1 (ppm)

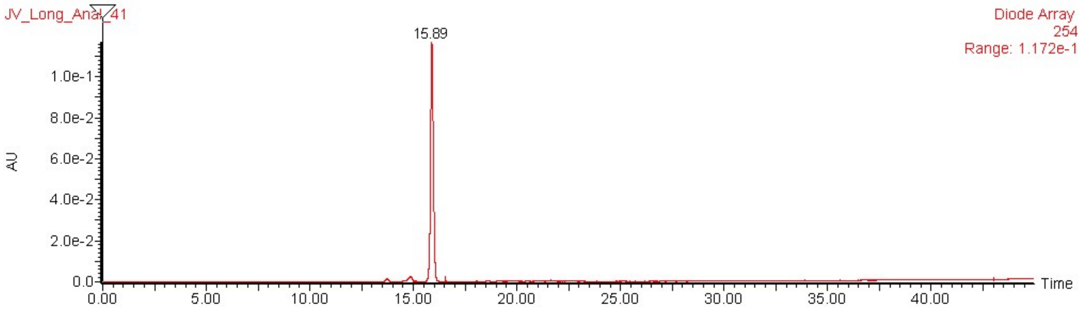

JV_Long_Anal_41
Diode Array
254
Range: 1.172e-1
15.89
AU
Time

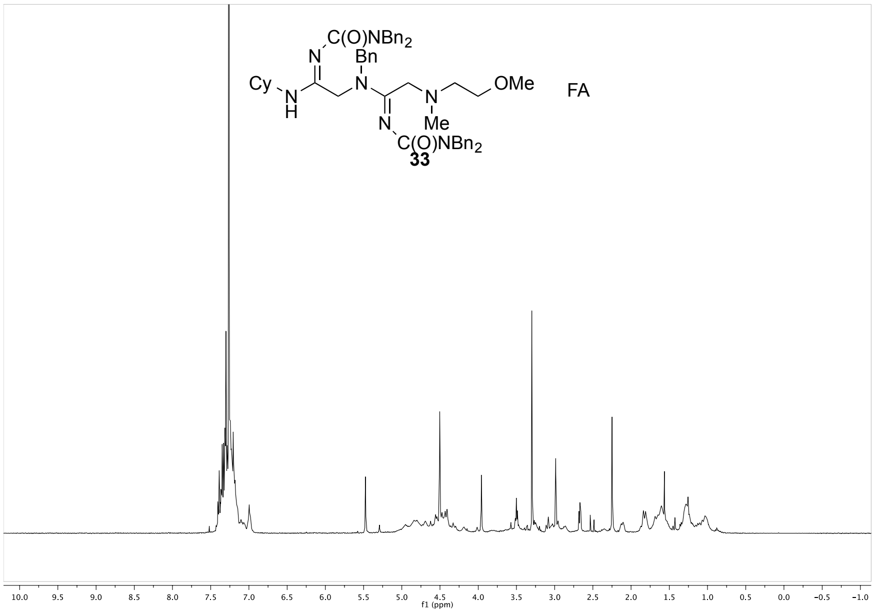
C(O)NBn2
Bn
Cy
N
H
OMe
FA
Me
C(O)NBn2
33
f1 (ppm)

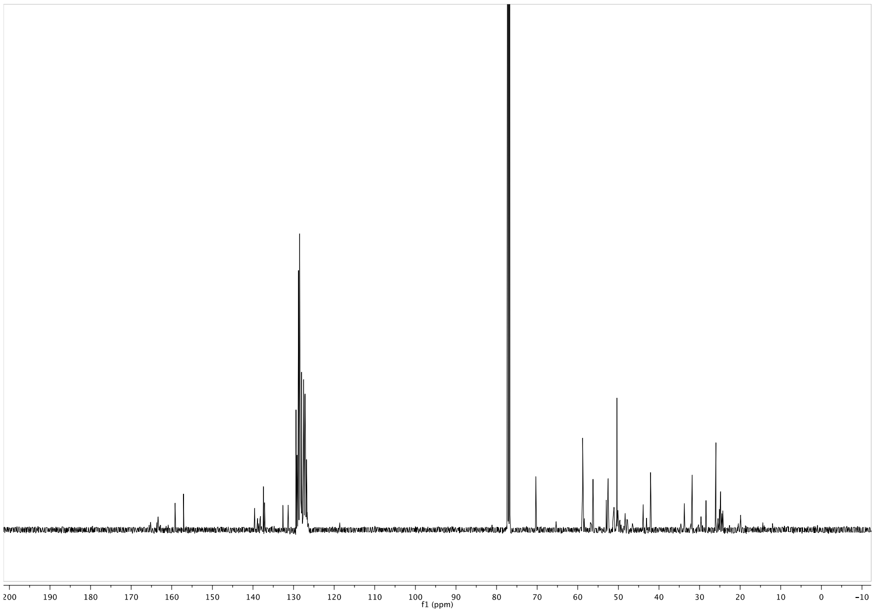
f1 (ppm)

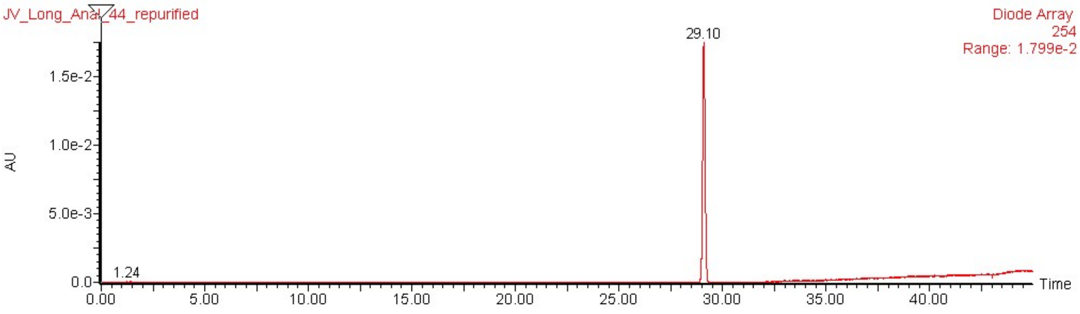
Diode Array
254
Range: 1.799e-2
29.10
1.24
AU
Time

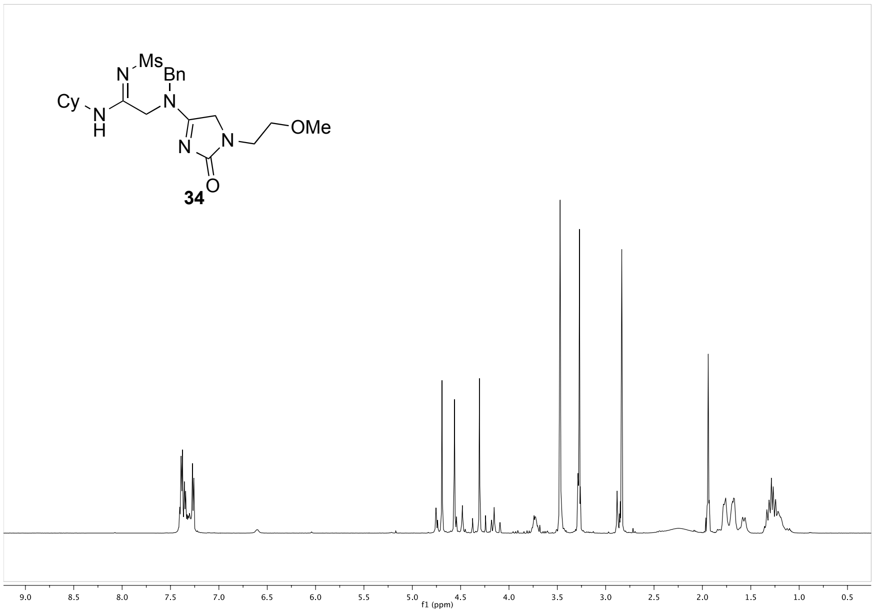
Ms
Bn
N
N
Cy
N
H
OMe
N
N
O
34
9.0 8.5 8.0 7.5 7.0 6.5 6.0 5.5 5.0 4.5 4.0 3.5 3.0 2.5 2.0 1.5 1.0 0.5
f1 (ppm)

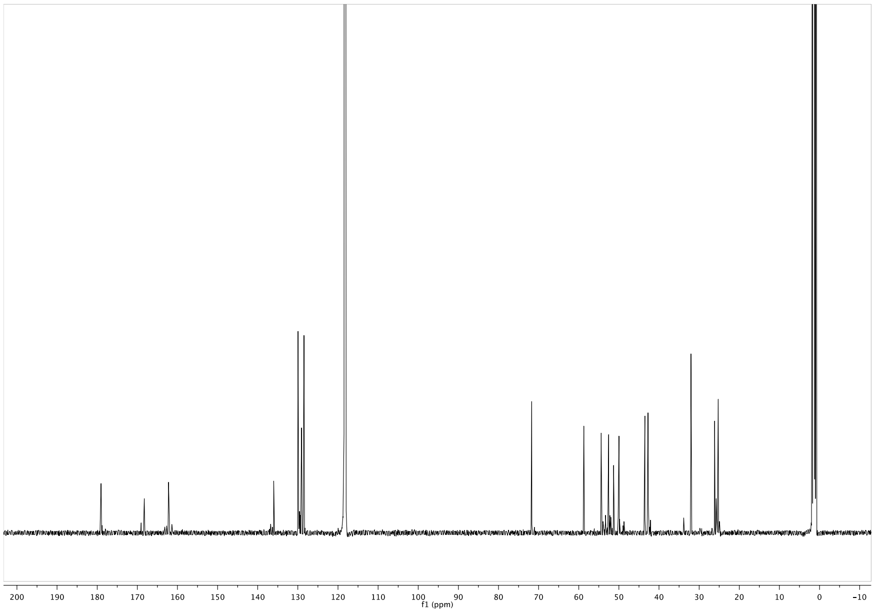
200 190 180 170 160 150 140 130 120 110 100 90 80 70 60 50 40 30 20 10 0 -10
f1 (ppm)

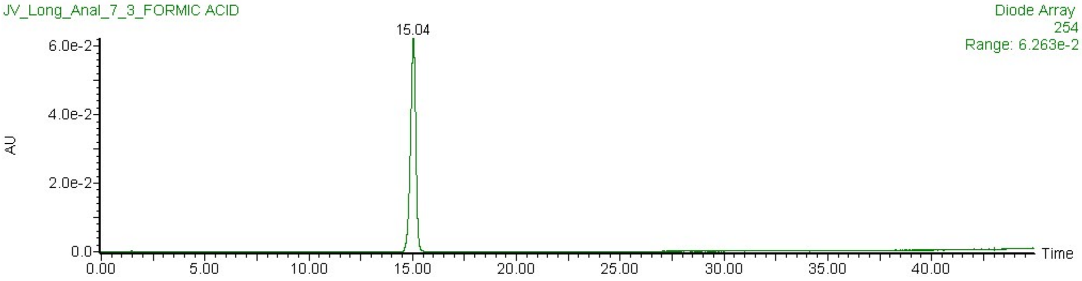
JV_Long_Anal_7_3_FORMIC ACID
Diode Array
254
Range: 6.263e-2
15.04
6.0e-2
4.0e-2
2.0e-2
0.0
AU
0.00 5.00 10.00 15.00 20.00 25.00 30.00 35.00 40.00
Time

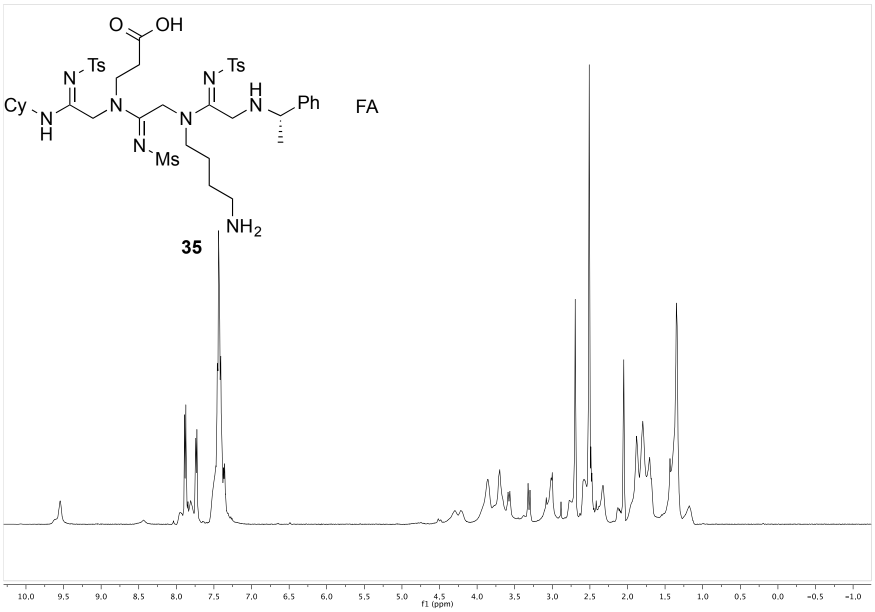
O
OH
N
Ts
Cy
N
H
Ms
Ph
FA
NH2
35
f1 (ppm)

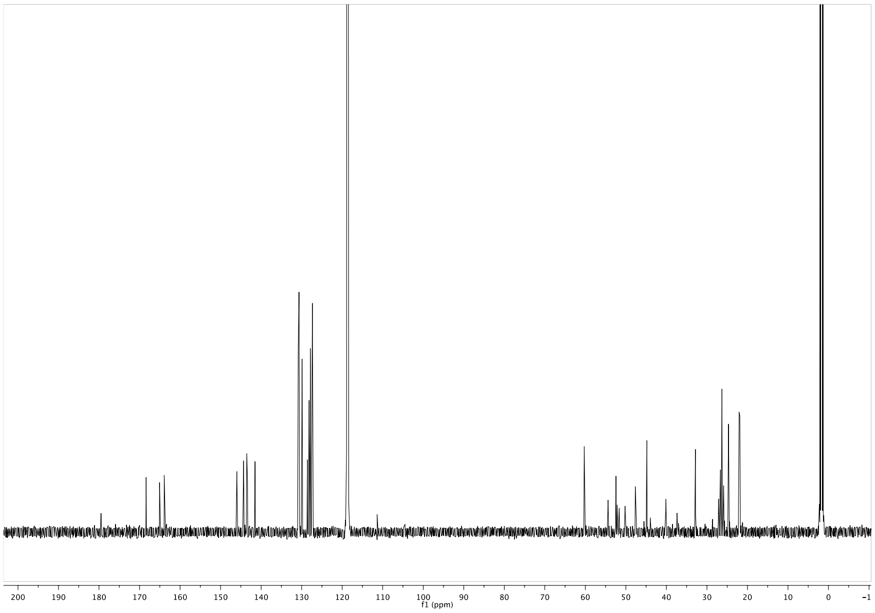
f1 (ppm)

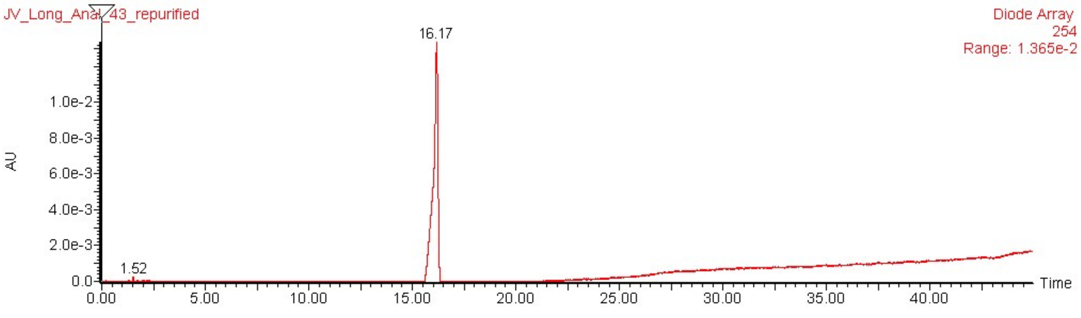
JV_Long_Anal_43_repurified
Diode Array
254
Range: 1.365e-2
16.17
1.52
AU
Time

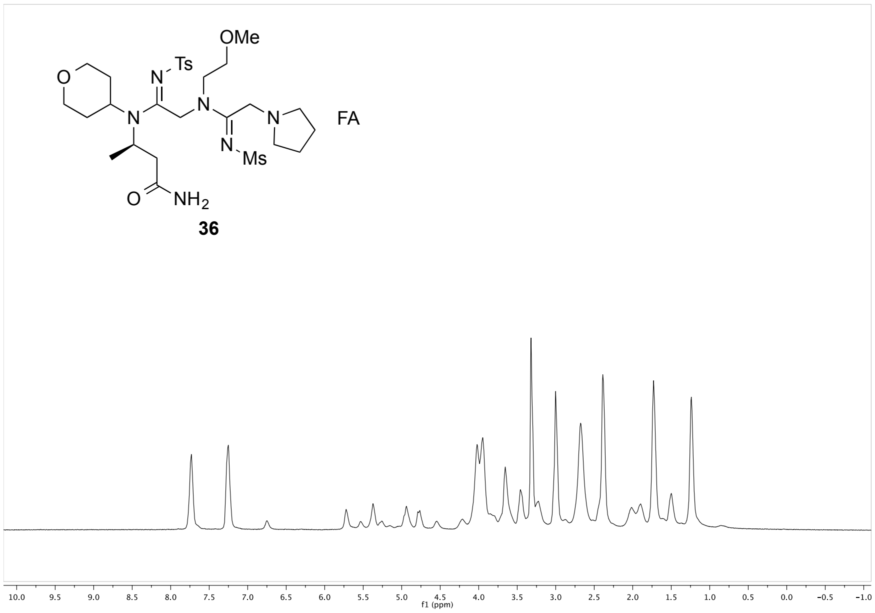

OMe
N
Ts
O
N
N
N
FA
N
Ms
O
NH2
36
f1 (ppm)

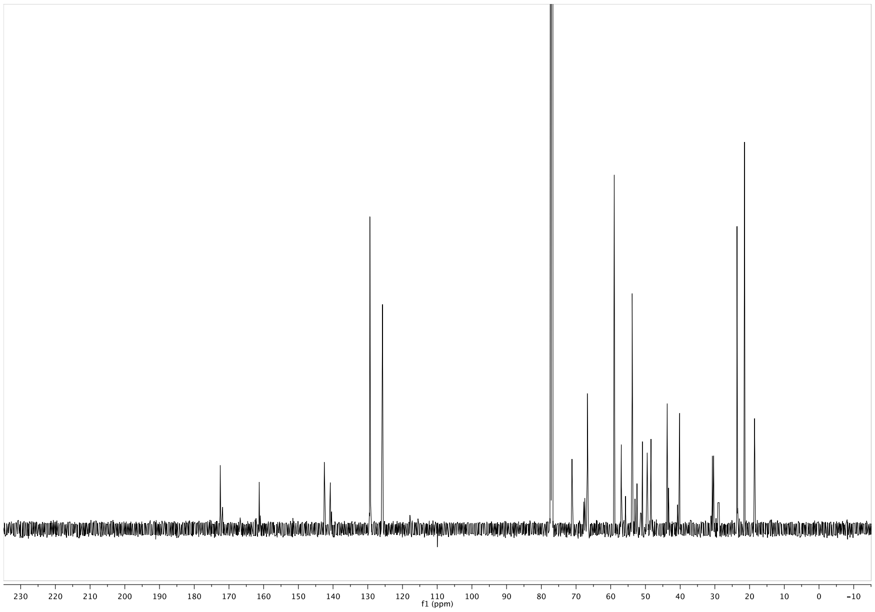

f1 (ppm)

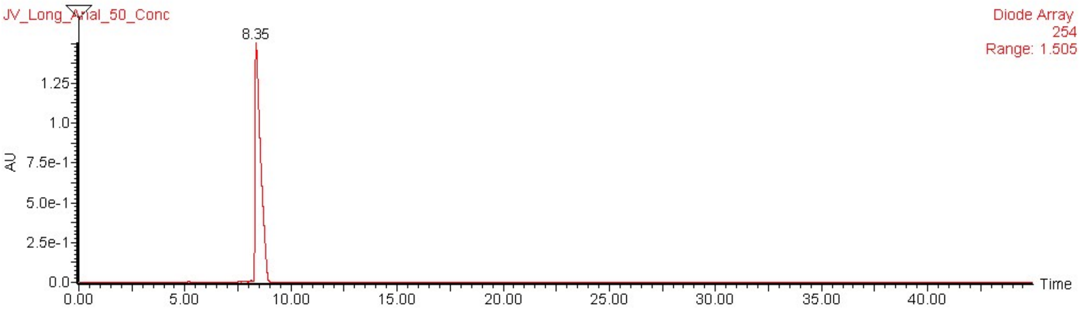

JV_Long_Anal_50_Conc
8.35
Diode Array
254
Range: 1.505
AU
Time

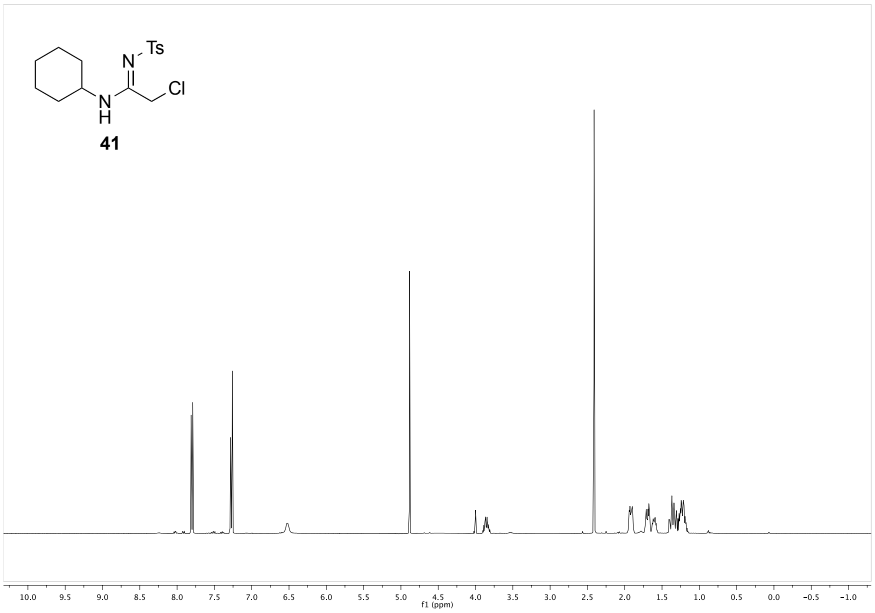
Ts
N
N
H
Cl
41
10.0
9.5
9.0
8.5
8.0
7.5
7.0
6.5
6.0
5.5
5.0
4.5
4.0
3.5
3.0
2.5
2.0
1.5
1.0
0.5
0.0
−0.5
−1.0
f1 (ppm)

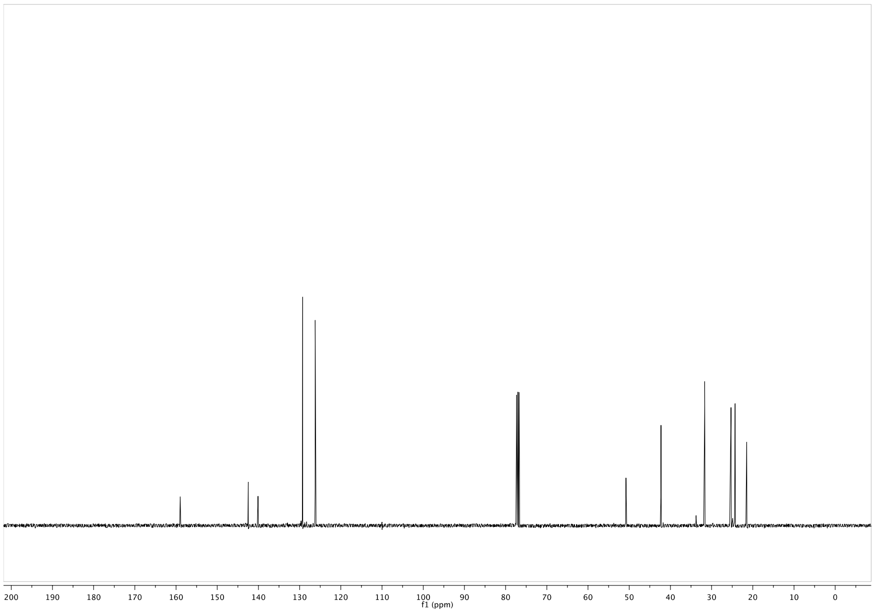
200
190
180
170
160
150
140
130
120
110
100
90
80
70
60
50
40
30
20
10
0
f1 (ppm)
